# DDQ catalyzed oxidative lactonization of indole-3-butyric acids†

**DOI:** 10.1039/d4ra05265j

**Published:** 2024-08-02

**Authors:** Christos Nixarlidis, John D. Chisholm

**Affiliations:** a Department of Chemistry, Syracuse University 1-014 Center for Science and Technology Syracuse NY 13244 USA jdchisho@syr.edu

## Abstract

Benzylic C–H bonds next to electron rich aromatic rings are susceptible to 2,3-dichloro-5,6-dicyano-1,4-benzoquinone (DDQ) promoted functionalization. In this work benzylic carbocations formed in this manner are trapped by a pendant carboxylic acid to form a lactone. Indole-3-butyric acids are especially good substrates for the reaction. The lactonization functions well with catalytic amounts of DDQ combined with MnO_2_ as the stoichiometric oxidant. This cyclization proceeds with a number of indole-3-butyric acids, and yields were generally good as long as the indole was electron rich and free of bulky substituents near the reacting benzylic carbon. Indole-3-butyric acids functionalized with electron withdrawing groups tend to give more moderate yields. Other aromatic substrates also participate as long as the aromatic ring is functionalized with electron donating groups.

## Abstract 2

Indole-3-butyric acids can be oxidized to lactones *via* 2,3-dichloro-5,6-dicyano-1,4-benzoquinone (DDQ) catalysis with MnO_2_ as the stoichiometric oxidant.

## Graphical abstract

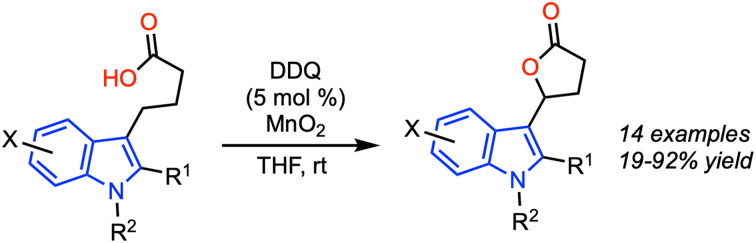

## Introduction

**Fig. 1:**
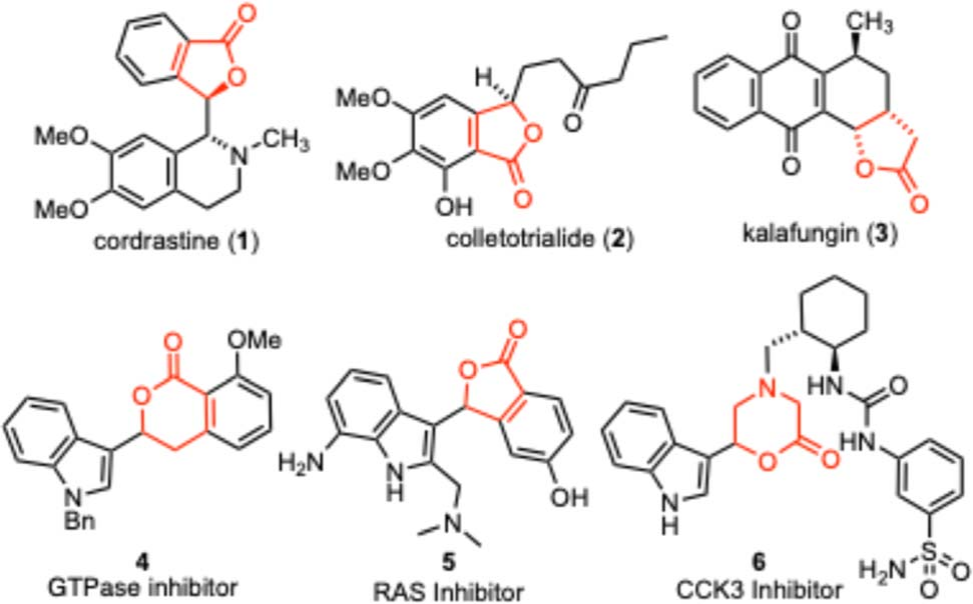
Examples of aryl substituted lactones and related structures.

Aryl substituted lactones are common functionality found in many useful structures. These systems have been isolated from natural sources and are often accessed synthetically to provide medicinally relevant compounds.^1,2^ Examples of natural products include the plant alkaloid cordrastine (1),^3^ antioxidants like colletotrialide 2 ^4^ and antimicrobials like kalafungin (3).^5^ The patent literature also documents a number of lactones decorated with indoles, including the GTPase inhibitor 4 ^6^ and the RAS inhibitor 5.^7^ Some cyclic ester containing heterocycles have also been reported, such as the CCK3 inhibitor 6 (Fig. 1).^8^

**Scheme 1:**
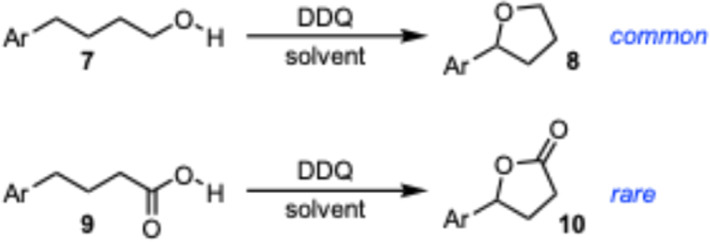
Synthesis of tetrahyrdofurans and lactones *via* cyclization with DDQ.

Given the prevalence of aryl substituted lactones, researchers have been active in defining methods to access these structures.^9,10^ In many cases the indole substituted butyric acids are easily accessible, therefore it may be possible to access bioactive molecules like 4–6 from the indole butyric acids by a benzylic C–H activation where the resulting cation is trapped by the pendant carboxylic acid. Similar cyclizations have been described that convert alcohols to cyclic ethers utilizing DDQ as a stoichiometric oxidant (Scheme 1).^11–22^ In contrast, only a single report describes the intramolecular cyclization of a carboxylic acid with a pendant electron rich benzene ring with DDQ,^23^ marking the transformation as significantly more rare.

We therefore began a study of the formation of indole-3-lactones *via* catalytic oxidative lactonization of indole-3-butyric acids using DDQ as the oxidant. In addition, we hoped that only a catalytic amount of DDQ would be required for this transformation. Over the last few years many reactions have been shown to be mediated by DDQ catalysis using a variety of reoxidants.^24–26^ Particularly interesting to us was the work of Liu and Floreancig, who described the use of catalytic DDQ using manganese dioxide as the stoichiometric oxidant.^27^ They successfully employed these conditions in a number of DDQ mediated transformations, including the intramolecular cyclization of a vinyl acetate to a pyran. Similar conditions were disclosed for the formation of diarylmethyl esters from carboxylic acids and diaryl methanes.^28^ Adaptation of these conditions to lactone formation could provide a useful organocatalytic C–H activation route to access complex lactones like 4–6 rapidly and efficiently. An investigation was therefore undertaken to explore the cyclization of indole butyric acids to lactones utilizing catalytic DDQ.

## Results and discussion

Initial studies on a DDQ catalyzed lactonization focused on indole-3-butyric acid 11 as the substrate (Table 1). The lactone product 12 was obtained in 76% yield when indole-3-butyric acid 9 was dissolved in THF (0.2 M) and stirred after the addition of 10 mol% DDQ and 5 equiv. of MnO_2_ at 25 °C for 18 hours (Entry 9). Other tested solvents such as DMF, DMAc, CH_3_CN, 1,4-dioxane, DCE, and DCM either gave a very low to moderate yield (Entries 1–5) or no reaction (Entry 6) due to the low solubility of the starting material in the solvent. With THF being the solvent of choice, some other variables were investigated in order to further improve the efficiency of this reaction. Decreasing the amount of MnO_2_ as well as the amount of DDQ led to an increase in the observed yield (Entries 8 and 9). When the reaction was performed either under reflux conditions or with a lower concentration, a significant decrease of the yield was observed (Entries 10 and 11). A low yield was also observed when 30 mol% of DDQ were used (Entry 12) due to difficulties removing the larger amount of DDQ, which required a second round of chromatography. Lower amounts of DDQ (less than 10 mol%) or MnO_2_ (less than 5 equiv.) led to significantly longer reaction times. The transformation was also performed on a gram scale (Entry 13), and provided the lactone product 12 in a 72% yield.

**Table 1:** Reaction optimization

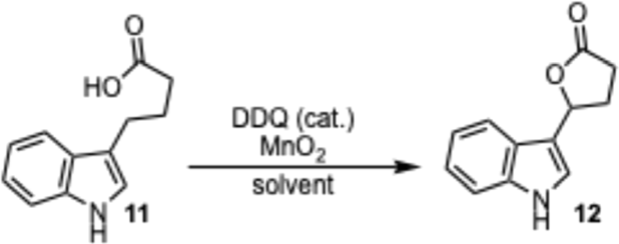					
Entry	mol% DDQ	Equiv. MnO_2_	Solvent	Temp. (°C)	Yield (%)
1	20	10	DMF	25	6
2	20	10	DMAc	25	5
3	20	10	CH_3_CN	25	42
4	20	10	1,4-Dioxane	25	30
5	20	10	DCE	25	54
6	20	10	DCM	25	0^c^
7	20	10	THF	25	63
8	20	5	THF	25	71
9	10	5	THF	25	76
10	10	5	THF	66	8
11^a^	10	5	THF	25	22
12	30	5	THF	25	41
13^b^	10	5	THF	25	72

a All reactions were performed at 0.2 M except Entry 11, which was performed at 0.5 M.

b Reaction was performed on 1 gram scale.

c Starting material was recovered.

The substrate scope of the lactonization of indole butyric acid derivatives was investigated (Table 1). The indole butyric acid substrates were synthesized by known alkylation methods (14,^29^16 ^30^ and 18 ^31^) or by acylation of the indole with ethyl succinyl chloride^32–34^ followed by reduction of the aryl ketone^35^ and hydrolysis of the ester (see the ESI† for full Experimental details). Functionalization of indole-butyric acid derivatives with different groups at the indole nitrogen was first explored (Table 2, Entries 2–5). Placement of a methyl group on the indole nitrogen gave a significant increase in yield (Table 2, Entry 2). This may be due to the electron donating effect of the methyl group increasing the reactivity of the indole to DDQ, or alternatively it may be due to slightly improved solubility of the carboxylic acid substrate. *N*-Allyl and *N*-benzyl substituted indoles were also tested, however the yield for the lactone was more moderate with the *N*-allyl substituted lactone 16. One explanation of the lower yield being observed for the lactone 16 is the possible activation of the C–H bond next to the *N*-allyl position. Similar reactions in the activation of allylic amines and related systems are known for DDQ.^36,37^ The presence of an electron withdrawing group such as a 1-naphthylsulfonyl (SO_2_Np) on the indole nitrogen gave no reaction, indicating that a pendant electron rich aromatic ring is necessary for the reaction to occur. This is consistent with the proposed mechanism of benzylic C–H activation of DDQ, where complexation of the DDQ to an electron rich aromatic system is required before C–H activation can occur.^38–41^

**Table 2:** Substituted indole substrates in the lactonization

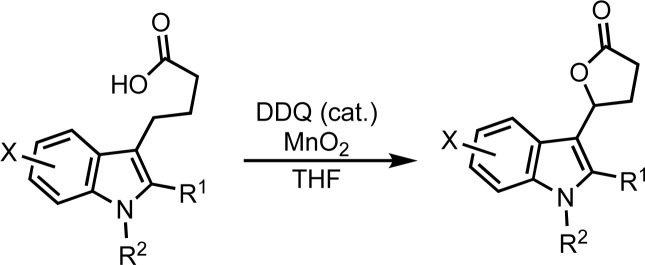						
Entry	Acid	X	R^1^	R^2^	Lactone	Yield (%)
1	11	H	H	H	12	76
2	13	H	H	Me	14	92
3	15	H	H	Allyl	16	37
4	17	H	H	Bn	18	74
5	19	H	H	SO_2_Np^a^	20	0^b^
6	21	H	Me	H	22	0^b^
7	23	5 F	H	H	24	19
8	25	5-Cl	H	H	26	62
9	27	5-Br	H	H	28	79
10	29	5-Me	H	H	30	47
11	31	5-OMe	H	H	32	85
12	33	5-CN	H	H	34	42
13	35	5-NO_2_	H	H	36	33
14	37	4-Cl	H	H	38	40
15	39	6-Cl	H	H	40	46
16	41	7-Cl	H	H	42	42

a SO_2_Np = 1-sulfonylnaphthalene.

b Starting material was recovered.

Substitution on the indole aromatic skeleton was then investigated (Table 2, Entries 6–16). When a methyl substituent was introduced on carbon 2 of the indole (21), no reaction was observed. This was attributed to steric shielding of the benzylic methylene by the methyl group. Better results were observed with substituents at the indole 5 position. Bromide, chloride and methoxy groups at this position all gave cyclization products in good yields. Electron withdrawing substituents were less well tolerated, as fluoride, nitro and cyano groups all gave more moderate yields. The incorporation of a 5-methyl group was also evaluated, but this gave a lower yield than the fluoro compound. This may be due to competitive C–H activation of the methyl group by the DDQ, which can lead to side reactions. Substitution at the 4, 6 and 7 positions were also evaluated with the incorporation of chlorides at these centers. These lactonizations all gave the expected product (38, 40 and 42).

Several mechanistic pathways for the oxidation of benzylic C–H bonds with DDQ have been proposed, and these mechanisms have been investigated by a number of groups.^42–49^ Detailed studies by Linstead, Jackman, and co-workers^50–53^ concluded that most DDQ oxidations occur through a rate-determining hydride transfer, leading to a delocalized carbocation which rapidly loses a proton or is trapped by a nucleophile. This mechanism is supported by *ab initio* calculations on the oxidation of cyclohexa-1,4-diene with DDQ that predicts an ionic mechanism in which an initial hydride transfer occurs.^54^ Computations also support the formation of carbocations when DDQ is employed.^38–41^

**Scheme 2:**
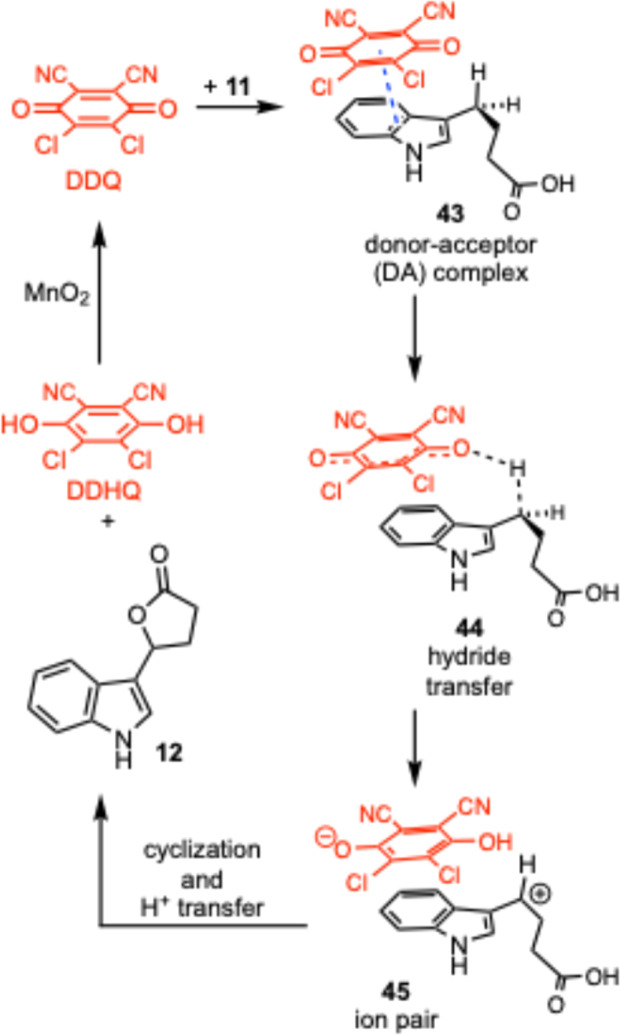
Proposed mechanism of the lactonization.

Based on these reports a proposed mechanism for the lactonization is shown in Scheme 2. Initially the DDQ forms a donor–acceptor complex with the indole (43). This puts one of the carbonyls of the DDQ in close proximity to a benzylic C–H bond, leading to a hydride transfer as shown in 44. This then provides an ion pair (45) with a benzylic carbocation and the reduced DDHQ anion. Cyclization of the carboxylic acid onto the carbocation followed by proton transfer gives the lactone 12 and the dihydroquinone DDHQ. The DDHQ is then reoxidized by the MnO_2_ to reform DDQ, which can re-enter the catalytic cycle.

**Scheme 3:**
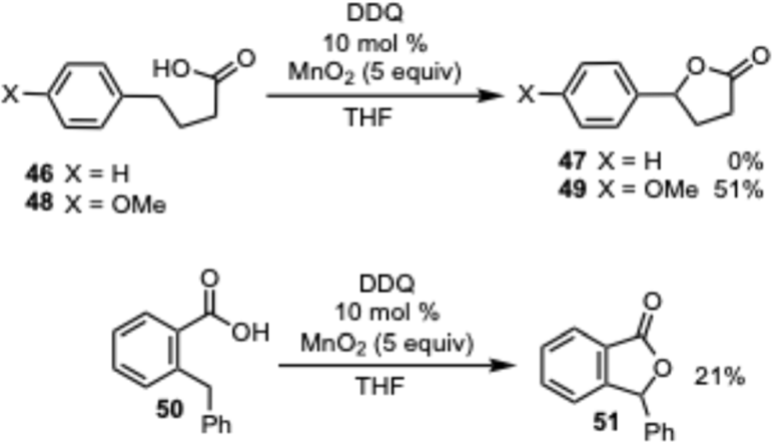
Lactonization of some alternative aromatic acids.

With the success in the formation of indole-3-butyric acids, we moved to test some other substrates in the DDQ promoted lactonization. While no product was observed in the case of 4-phenyl butyric acid 46 (starting 46 was recovered unchanged), a 51% yield of the lactone was obtained with 4-(4-methoxyphenyl)butyric acid 48. This difference is likely due to the difficulty in activating the benzylic C–H bond in a substrate without an electron rich aromatic ring. Attempts to remedy this with the addition of a second aromatic ring were examined by using 2-benzyl benzoic acid 50, which was able to provide some lactone product (21%) despite not having a strong electron donating group attached to one of the aromatic rings. These results indicate that the lactonization can be performed on substrates other than indole provided that the C–H bond to activated is next to an electron rich aromatic ring or activated in a similar manner (Scheme 3).

## Conclusion

We have shown that lactones can be formed through intramolecular cyclization under mild conditions utilizing a catalytic amount of DDQ in combination with a MnO_2_ as the stoichiometric oxidant. This combination provides good yields and has the advantage that the purification of the quinone from the product is much easier with a smaller amount of DDQ. The presence of electron donating groups on the indole ring of the butyric acid substrates leads to faster reactions and higher yields. Alternatively, substrates with electron withdrawing groups give lower yields. When bulky substituents were tested on the indole N or at the C-2 of the indole ring, no reaction was observed due to steric effects. Other aromatic rings besides indole also participate in the lactonization as long as they are decorated with electron donating substituents.

## Experimental

A complete Experimental section, including ^1^H and ^13^C NMR spectra, is provided as ESI.†

## Data availability

The data supporting this article have been included as part of the ESI.†

## Conflicts of interest

There are no conflicts to declare.

## Supplementary Material

RA-014-D4RA05265J-s001
