# Supplementary material for: DDQ catalyzed oxidative lactonization of indole-3-butyric acids
Source: RSC Adv. 2024 Aug 2;14(33):24207–11. doi: 10.1039/d4ra05265j (PMC11294987; doi:10.1039/d4ra05265j)
Supplement: RA-014-D4RA05265J-s001 [file RA-014-D4RA05265J-s001.pdf]

Supplementary Information (SI) for RSC Advances.
This journal is © The Royal Society of Chemistry 2024

Supporting Information

DDQ Catalyzed Oxidative Lactonization of Indole-3-Butyric Acids

*Christos Nixarlidis and John D. Chisholm**

Department of Chemistry, Syracuse University, 1-014 Center for Science and Technology, Syracuse, NY 13244

jdchisho@syr.edu

Contents

Table of Contents	S1
Experimental Procedures and Tabulated Data	S2-S17
NMR Spectra	S18-S73
References	S74

General Experimental Information

All anhydrous reactions were run under a positive pressure of argon. Dichloromethane (DCM) and 1,2-dichloroethane (1,2-DCE) were freshly distilled from calcium hydride before use. Tetrahydrofuran (THF) was distilled from Na/benzophenone before use. Ethyl acetate (EA), hexanes and other solvents were used as received from the manufacturer.

Identity. The chemical shifts are given in parts per million (ppm) on the delta (δ) scale. Coupling constants are reported in hertz (Hz). For spectra recorded in solutions of deuterated chloroform ($CDCl_3$), residual chloroform or TMS was used as the internal reference. Data are reported as follows: (s = singlet; d = doublet; t = triplet; q = quartet; p = pentet; dd = doublet of doublets; dt = doublet of triplets; td = triplet of doublets; tt = triplet of triplets; qd = quartet of doublets; ddd = doublet of doublet of doublets; br s = broad singlet). Where applicable, the number of protons attached to the corresponding carbon atom was determined by DEPT135 NMR. Infrared (IR) spectra were obtained neat using an attenuated total reflectance (ATR) attachment.

Analysis and Purity. Analytical thin layer chromatography (TLC) was performed on precoated glass backed plates (silica gel 60 F254; 0.25 mm thickness). The TLC plates were visualized by UV illumination and by staining. Solvents for chromatography are listed as volume:volume ratios. Column chromatography was conducted on silica gel (40-63 µm). Melting points were recorded using an electrothermal melting point apparatus and are uncorrected. Elemental analyses were performed on an elemental analyzer with a thermal conductivity detector and 2-meter GC column maintained at 50 °C.

Synthesis of Indole Butyric Acids

Indole butyric acid (**11**) was purchased from commercial vendors. 3-(1-Methyl-1H-indol-3-yl) butanoic acid (**13**),[1] 1-allyl-1H-indole-3-butanoic acid (**15**),[2] and 4-(1-benzyl-1H-indol-3-yl)-butyric acid (**17**)[3] were synthesized as previously reported. 4-[1-(Naphthalenesulfonyl)-1H-indol-3-yl]butanoic acid (**19**) was prepared as shown in Scheme S1 below.

Scheme S1

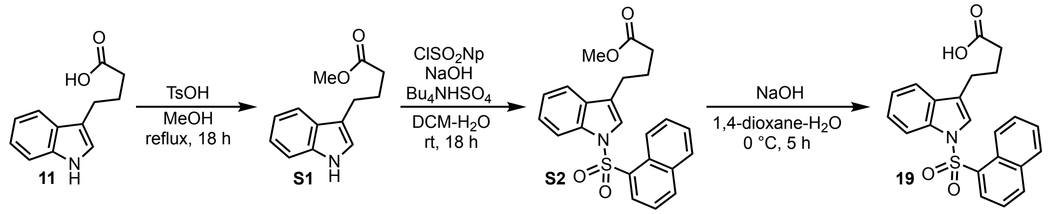

Methyl 4-(1H-indol-3-yl)butanoate (S1).

In a round bottom flask equipped with stir bar, indole-3-butyric acid **11** (1.0 g, 4.9 mmol) and p-toluenesulfonic acid (0.93 g, 4.9 mmol) were dissolved in 6.5 mL of methanol. The reaction mixture was brought to reflux overnight. The mixture was then cooled, and the methanol was evaporated. The residue was dissolved in EA and then washed with $NaHCO_3$ and brine. The organic phase was dried with sodium sulfate, filtered and reduced under vacuum. Compound **S1** was obtained as a yellow oil in 71% yield (0.76 g).

(S1). TLC Rf = 0.68 (hexanes : EA 50%:50%); IR (film, cm^{-1}) ν_{max} 3331, 3043, 2949, 1713, 1459; ^1H NMR (400 MHz, $CDCl_3$) δ 7.97 (s, 1H), 7.63 (d, *J* = 7.9 Hz, 1H), 7.38 (d, *J* = 8.2 Hz, 1H), 7.22 (dt, *J* = 7.1, 0.9 Hz, 1H), 7.14 (dt, *J* = 7.9, 0.9 Hz, 1H), 7.02 (s, 1H), 3.69 (s, 3H), 2.83 (t, *J* = 7.5 Hz, 2H), 2.42 (t, *J* = 7.5 Hz, 2H), 2.08 (pent, *J* = 7.5 Hz, 2H); ^{13}C NMR (100 MHz, $CDCl_3$) δ 174.2, 136.4, 127.4, 122.0, 121.4, 119.2, 118.9, 115.6, 111.1, 51.5, 33.7, 25.3, 24.5. This compound has been reported previously.[4]

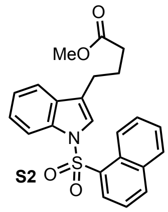

Methyl 4-[1-(naphthalene sulfonyl)-1H-indol-3-yl]butanoate (S2)

In a flame dried round bottom flask equipped with stir bar, 4.14 mmol (0.9 g) of **11**, 7.25 mmol (1.38 g) of 1-napthalene sulfonyl chloride and 0.41 mmol (0.14 g) of Bu_4NHSO_4 were dissolved in 18.0 mL of DCM. The reaction mixture was cooled at 0 ^{0}C and then NaOH (6.21 mmol, 0.25 g) was added in portions. The reaction mixture was stirred at room temperature overnight. Then, the reaction was quenched with water and stirred vigorously for 10 minutes. Then, the solution was transferred to separatory funnel and the aqueous layer was extracted three times with dichloromethane. The combined organic layers were washed with brine, dried with magnesium sulfate and the solvent was removed with rotary evaporator. Purification by silica gel chromatography (100% DCM) afforded **12** as a white solid in 42% yield (0.72 g).

(S2) mp = 95–98 °C, TLC R_f= 0.76 (DCM 100%); IR (film, cm^{-1}) ν_{max} 3109, 2941, 1740, 1506, 1415, 1354; ^{1}H NMR (400 MHz, $CDCl_3$) δ 8.74 (d, *J* = 8.7 Hz, 1H), 8.11 (dd, *J* = 7.4, 1.0 Hz, 1H), 8.05 (d, *J* = 8.2 Hz, 1H), 7.89 (d, *J* = 8.1 Hz, 1H), 7.82 (d, *J* = 8.3 Hz, 1H), 7.68-7.64 (m, 1H), 7.59 (s, 1H), 7.57-7.49 (m, 3H), 7.26-7.20 (m, 2H), 3.71 (s, 3H), 2.76 (t, *J* = 7.3 Hz, 2H), 2.37 (t, *J* = 7.3 Hz, 2H), 2.04 (pent, *J* = 7.3 Hz, 2H); ^{13}C NMR (100 MHz, DMSO-d_6) δ 173.6, 136.5, 134.6, 134.3, 132.8, 130.7, 130.7, 129.9, 129.4, 127.9, 127.5, 125.1, 125.1, 124.4, 123.8, 123.6, 121.5, 120.3, 113.3, 51.7, 33.0, 24.4, 23.7; Anal. calcd for $C_{23}H_{21}NO_4S$: C, 67.79; H, 5.19; N, 3.44. Found: C, 67.61; H, 5.10; N, 3.52.

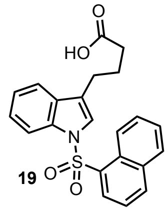

4-[1-(naphthalenesulfonyl)-1H-indol-3-yl]butanoic acid (19)

In a round bottom flask equipped with stir bar, 0.17 mmol (0.07 g) of the ester **12** was dissolved in 1,4 dioxane (0.4 M). Then, 1.0 mL of 1M NaOH were added and the solution was let it stir for 5 hours at 0 degree Celsius. The reaction mixture was then acidified with HCl and transferred to separatory funnel. The aqueous layer was extracted three times with DCM. The combined organic layers were dried with sodium sulfate and the solvent was removed with rotary evaporator. Purification by silica gel chromatography (90% DCM/ 10% MeOH) afforded **19** as a white solid in 28% yield (0.019 g).

(19) mp = 228–231 °C, TLC R_f= 0.4 (DCM 90%/ MeOH 10%); IR (film, cm^{-1}) ν_{max} 3316, 2934, 2609, 1706, 1591, 1458; ^{1}H NMR (400 MHz, DMSO-d_6) δ 12.09 (s, 1H), 8.64 (d, *J* = 8.6 Hz, 1H), 8.38 (d, *J* = 6.8 Hz, 1H), 8.31 (d, *J* = 8.2 Hz, 1H), 8.08 (d, *J* = 8.0 Hz, 1H), 7.89 (s, 1H), 7.74-7.69 (m, 3H), 7.65 (t, *J* = 7.1 Hz, 1H), 7.45 (d, *J* = 7.4 Hz, 1H), 7.26 (t, *J* = 7.4 Hz, 1H), 7.22 (t, *J* = 7.0 Hz, 1H), 2.69 (t, *J* = 7.3 Hz, 2H), 2.21 (t, *J* = 7.3 Hz, 2H), 1.85 (pent, *J* = 7.3 Hz, 2H); ^{13}C NMR (100 MHz, DMSO-d_6) δ 174.8, 136.5, 134.6, 134.3, 132.8, 130.7, 130.7, 129.9, 129.4, 127.9, 127.5, 125.1, 125.1, 124.3, 123.8, 123.5, 121.7, 120.3, 113.3, 33.5, 24.5, 23.8; Anal. calcd for $C_{22}H_{19}NO_4S$: C, 67.16; H, 4.87; N, 3.56. Found: C, 67.00; H, 5.08; N, 3.47.

Additional indole butyric acids were synthesized by acylation[5] of the commercially available indoles (**S3**-**S12**) with ethyl succinyl chloride to provide the acylated indoles (**S14**-**S24**) as shown in Scheme S2. The ketone was then reduced with sodium borohydride and TFA,[6] and the ester was hydrolyzed to provide the indole 3-butyric acids. Ketone **S16** was prepared using $ZrCl_4$[7] instead of Et_2AlCl.

Scheme S2

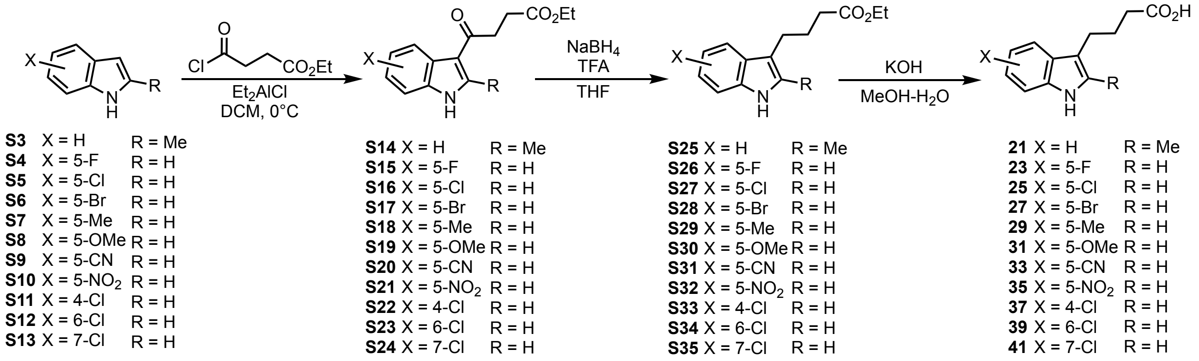

General Procedure for the Acylation of Indoles:[5,8]

In a flame dried round bottom flask equipped with a stir bar, 1 equiv. of substituted indole was dissolved in dry DCM (0.2M) and let it stir at 0 °C. Then, 1.5 equiv. of diethyl aluminum chloride (1.0 M in hexane) was added, and the reaction was stirred at 0 °C. After 30 min, 1.5 equiv. of ethyl succinyl chloride was added and the reaction mixture was stirred for 2 hours. After 2 hours, the reaction was quenched slowly with cold NH_4Cl and extracted twice with EA. The combined organic layers were washed with Rochelle salt, saturated sodium bicarbonate, dried with Na_2SO_4 and reduced under pressure. The residue was purified by silica gel flash column chromatography to provide the ketone product.

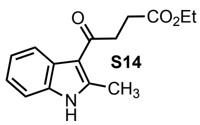

S14

Ethyl 4-(2-methyl-1H-indole-3-yl)-4-oxobutanoate (S14) (0.79 g, 40%) was prepared according to the representative procedure from 2-methyl-indole (1.0 g, 7.6 mmol). Purification by silica gel chromatography (50% EA/ 50% hexanes) afforded **S14** as a brown solid. mp = 129-131 °C; TLC R_f = 0.42 (hexanes: EA 50%:50%); IR (film, cm^{-1}) ν_{max} 3269, 3053, 2989, 1707, 1625, 766; ^{1}H NMR (400 MHz, $CDCl_3$) δ 8.63 (bs, 1H), 8.03 (d, J = 7.5 Hz, 1H), 7.34 (dd, J = 7.2, 1.1 Hz, 1H), 7.27-7.19 (m, 2H), 4.22 (q, J = 7.1 Hz, 2H), 3.33 (t, J = 6.7 Hz, 2H), 2.83 (t, J = 6.7 Hz, 2H), 2.72 (s, 3H), 1.31 (t, J = 7.1 Hz, 3H); ^{13}C NMR (100 MHz, $CDCl_3$) δ 194.5, 173.6, 143.9, 134.5, 126.6, 122.4, 122.1, 120.9, 113.8, 110.9, 60.6, 37.5, 28.3, 15.6, 14.2; Anal. calcd for $C_{15}H_{17}NO_3$: C, 69.48; H, 6.61; N, 5.40. Found: C, 69.26; H, 6.53; N, 5.20.

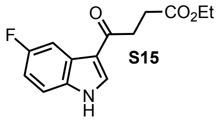

S15

Ethyl 4-(5-fluoro-1H-indole-3-yl)-4-oxobutanoate (S15) (0.71 g, 36%) was prepared according to the representative procedure from 5-fluoro-indole (1.0 g, 7.4 mmol). Purification by silica gel chromatography (95% DCM/ 5% EA) afforded **S15** as a brown solid. mp = 153–155 °C TLC R_f = 0.35 (hexanes: EA 50%:50%); IR (film, cm^{-1}) ν_{max} 3328, 2962, 2915, 1729, 1632, 1520, 1432, 1224; ^{1}H NMR (400 MHz, $CDCl_3$) δ 9.2 (bs, 1H), 8.00 (dd, J = 9.7, 2.3 Hz, 1H), 7.83 (d, J = 3.0 Hz, 1H), 7.33-7.29 (m, 1H), 7.00 (td, J = 8.9, 2.4 Hz, 1H), 4.20 (q, J = 7.1 Hz, 2H), 3.17 (t, J = 6.6 Hz, 2H), 2.81 (t, J = 6.6 Hz, 2H), 1.30 (t, J = 7.1 Hz, 3H); ^{13}C NMR (100 MHz, $CDCl_6$) δ 193.5, 173.7, 159.6 (d, J = 239 Hz), 132.7, 132.5, 126.1 (d, J = 11.2 Hz), 117.5, 112.3, 112.1 (d, J = 26.2 Hz), 107.6 (d, J = 25.2 Hz), 60.8, 33.9, 28.4, 14.2; ^{19}F NMR (376.5 MHz, $CDCl_3$) δ -120,7; Anal. calcd for $C_{14}H_{14}FNO_3$: C, 63.87; H, 5.36; N, 5.32. Found: C, 64.20; H, 5.43; N, 5.54.

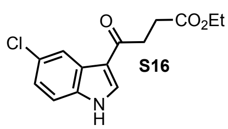

S16

Ethyl 4-(5-chloro-1H-indole-3-yl)-4-oxobutanoate (S16) (1.03 g, 56%) was prepared according to the representative procedure from 5-chloro-indole (1.0 g, 6.6 mmol). Purification by silica gel chromatography (50% EA/ 50% hexanes) afforded **S16** as a yellow solid. mp = 155-157 °C; TLC R_f = 0.30 (hexanes: EA 50%:50%); IR (film, cm^{-1}) ν_{max} 3211, 3049, 2984, 1723, 1626, 1434, 1141; ^{1}H NMR (400 MHz, DMSO-*d$_6$*) δ 12.12 (bs, 1H), 8.45 (s, 1H), 8.14 (d, *J* = 1.8 Hz, 1H), 7.50 (d, *J* = 8.6 Hz, 1H), 7.23 (dd, *J* = 8.6, 2.0 Hz, 1H), 4.06 (q, *J* = 7.1 Hz, 2H), 3.17 (t, *J* = 6.2 Hz, 2H), 2.64 (t, *J* = 6.2 Hz, 2H), 1.18 (t, *J* = 7.1 Hz, 3H); ^{13}C NMR (100 MHz, DMSO-*d$_6$*) δ 193.7, 173.0, 135.6, 126.9, 126.9, 123.2, 120.8, 115.9, 114.2, 60.3, 33.9, 28.4, 14.6; Anal. calcd for $C_{14}H_{14}ClNO_3$: C, 60.12; H, 5.05; N, 5.01. Found: C, 60.08; H, 5.11; N, 4.78.

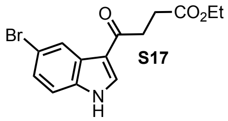

S17

Ethyl 4-(5-bromo-1H-indole-3-yl)-4-oxobutanoate (S17). In a flame dried round bottom flask equipped with a stir bar, 5-bromo-indole (5.1 mmol, 1.0 g) was dissolved in DCE (42 mL) and stirred at 0 °C. Then, 5.86 mmol (1.37 g) of $ZrCl_4$ was added and the reaction was stirred at 0 degrees for 30 minutes. After 30 minutes, 3.93 mmol (0.56 mL) of ethyl succinyl chloride was added and the reaction mixture was stirred for 2 hours. After 2 hours, the reaction was quenched with water and extracted twice with EA. The combined organic layers were washed with water, dried with Na_2SO_4 and reduced under pressure. Purification by silica gel chromatography (50% EA/ 50% hexanes) afforded **S17** as a brown solid in 24% yield (0.39 g).

(S17) TLC R_f = 0.23 (hexanes : EA 50%:50%); IR (film, cm^{-1}) ν_{max} 3236, 2985, 2923, 1723, 1626, 1611, 1521; ^{1}H NMR (400 MHz, DMSO-*d$_6$*) δ 12.14 (bs, 1H), 8.43 (s,1H), 8.29 (d, *J* = 1.8 Hz, 1H), 7.45 (d, *J* = 8.6 Hz, 1H), 7.35 (dd, *J* = 8.6, 2.0 Hz, 1H), 4.06 (q, *J* = 7.1 Hz, 2H), 3.17 (t, *J* = 6.2 Hz, 2H), 2.64 (t, *J* = 6.2 Hz, 2H), 1.18 (t, *J* = 7.1 Hz, 3H); ^{13}C NMR (100 MHz, DMSO-*d$_6$*) δ 193.7, 173.0, 135.7, 135.5, 127.5, 125.8, 123.8, 115.8, 114.9, 114.7, 60.3, 33.9, 28.4, 14.6. This compound has been previously prepared.[8]

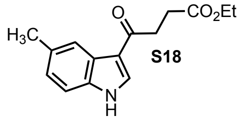

S18

Ethyl 4-(5-methyl-1H-indole-3-yl)-4-oxobutanoate (S18) (0.73 g, 37%) was prepared according to the representative procedure from 5-methyl-indole (1.0 g, 7.6 mmol). Purification by silica gel chromatography (50% EA/ 50% hexanes) afforded **S18** as a brown solid. mp = 186-189 °C; TLC R_f = 0.41 (hexanes: EA 50%:50%); IR (film, cm^{-1}) ν_{max} 3221, 3050, 2917, 1724, 1613, 1436, 1150; ^{1}H NMR (400 MHz, $CDCl_3$) δ 8.56 (bs, 1H), 8.18 (s, 1H), 7.86 (d, *J* = 3.1 Hz, 1H), 7.29 (d, *J* = 8.3 Hz, 1H), 7.10 (d, *J* = 8.4 Hz, 1H), 4.17 (q, *J* = 7.1 Hz, 2H), 3.21 (t, *J* = 6.9 Hz, 2H), 2.79 (t, *J* = 6.9 Hz, 2H), 2.47 (s, 3H), 1.27 (t, *J* = 7.1 Hz, 3H); ^{13}C NMR (100 MHz, $CDCl_3$) δ 193.7, 173.4, 134.5, 132.3, 131.1, 125.7, 125.3, 122.1, 117.4, 110.9, 60.6, 34.2, 28.5, 21.5, 14.2; Anal. calcd for $C_{15}H_{17}NO_3$: C, 69.48; H, 6.61; N, 5.40. Found: C, 69.60; H, 6.40; N, 5.36.

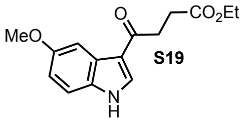

S19

Ethyl 4-(5-methoxy-1H-indole-3-yl)-4-oxobutanoate (S19) (0.43 g, 23%) was prepared according to the representative procedure from 5-methoxy-indole (1.0 g, 6.8 mmol). Purification by silica gel chromatography (95% DCM/ 5% methanol) afforded **45** as a white solid. TLC R_f = 0.37 (hexanes:EA 50%:50%); IR (film, cm^{-1}) ν_{max} 3165, 2981, 1728, 1615, 1468, 1285; ^{1}H NMR (400 MHz, DMSO-*d$_6$*) δ 11.8 (bs, 1H), 8.29 (s,1H), 7.67 (d, *J* = 2.4 Hz, 1H), 7.36 (d, *J* = 8.8 Hz, 1H), 6.84 (dd, *J* = 8.8, 2.5 Hz, 1H), 4.06 (q, *J* = 7.1 Hz, 2H), 3.77 (s, 3H), 3.15 (t, *J* = 6.2 Hz, 2H), 2.62 (t, *J* = 6.2 Hz, 2H), 1.18 (t, *J* = 7.1 Hz, 3H); ^{13}C NMR (100 MHz, DMSO-

d6) δ 193.5, 173.1, 155.8, 134.4, 131.8, 126.5, 116.2, 113.3, 113.1, 103.3, 60.3, 55.7, 33.7, 28.5, 14.6. This compound has been previously prepared.[8]

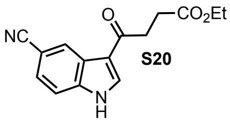

Ethyl 4-(5-cyano-1H-indole-3-yl)-4-oxobutanoate (S20) (0.59 g, 31%) was prepared according to the representative procedure from 5-cyano-indole (1.0 g, 7.0 mmol). Purification by silica gel chromatography (70% EA/ 30% hexanes) afforded **S20** as a brown solid. mp = 142–145 ºC TLC R_f = 0.24 (hexanes: EA 70%:30%); IR (film, cm^{-1}) ν_{max} 3275, 2983, 2913, 2223, 1727, 1632, 1442, 1230; ^{1}H NMR (400 MHz, DMSO-*d6*) δ 12.4 (bs, 1H), 8.59 (s,1H), 8.52 (d, *J* = 0.9 Hz, 1H), 7.66 (dd, *J* = 8.4, 0.4 Hz, 1H), 7.60 (dd, *J* = 8.4, 1.6 Hz, 1H), 4.06 (q, *J* = 7.1 Hz, 2H), 3.21 (t, *J* = 6.1 Hz, 2H), 2.66 (t, *J* = 6.6 Hz, 2H), 1.18 (t, *J* = 7.1 Hz, 3H); ^{13}C NMR (100 MHz, DMSO-*d6*) δ 193.9, 172.9, 138.9, 136.6, 126.7, 126.2, 125.5, 120.6, 116.5, 114.1, 104.4, 60.3, 34.0, 28.3, 14.6; Anal. calcd for $C_{15}H_{14}N_2O_3$: C, 66.66; H, 5.22; N, 10.36. Found: C, 66.56; H, 4.89; N, 10.33.

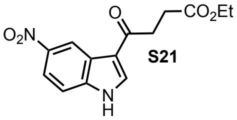

Ethyl 4-(5-nitro-1H-indole-3-yl)-4-oxobutanoate (S21) (0.73 g, 41%) was prepared according to the representative procedure from 5-nitro-indole (1.0 g, 6.2 mmol). Purification by silica gel chromatography (95% DCM/ 5% EA) afforded **S21** as a brown solid. mp = 205–208 ºC; TLC R_f = 0.23 (hexanes: EA 40%:60%); IR (film, cm^{-1}) ν_{max} 3180, 3110, 3058, 2983, 1721, 1633, 1534, 1144; ^{1}H NMR (400 MHz, DMSO-*d6*) δ 12.57 (bs, 1H), 9.03 (d, *J* = 2.2 Hz, 1H), 8.66 (s, 1H), 8.12 (dd, *J* = 9.0, 2.4 Hz, 1H), 7.68 (d, *J* = 9.0 Hz, 1H), 4.07 (q, *J* = 7.1 Hz, 2H), 3.23 (t, *J* = 6.3 Hz, 2H), 2.67 (t, *J* = 6.3 Hz, 2H), 1.18 (t, *J* = 7.1 Hz, 3H); ^{13}C NMR (100 MHz, DMSO-*d6*) δ194.1, 173.0, 143.2, 140.2, 137.7, 125.1, 118.7, 118.1, 117.6, 113.4, 60.3, 34.0, 28.3, 14.6; Anal. calcd for $C_{14}H_{14}N_2O_5$: C, 57.93; H, 4.86; N, 9.65. Found: C, 57.71; H, 4.98; N, 9.71.

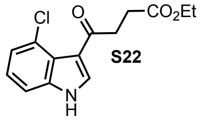

Ethyl 4-(4-chloro-1H-indole-3-yl)-4-oxobutanoate (S22) (0.71 g, 38%) was prepared according to the representative procedure from 7-methyl-indole (1.0 g, 6.6 mmol). Purification by silica gel chromatography (50% EA/ 50% hexanes) afforded **61** as a beige solid. mp = 155-158 ºC; TLC R_f = 0.31 (hexanes: EA 50%:50%); IR (film, cm^{-1}) ν_{max} 3315, 2989, 1726, 1647, 1566; ^{1}H NMR (400 MHz, DMSO-*d6*) δ 12.19 (bs, 1H), 8.42 (s, 1H), 7.47-7.42 (m, 1H), 7.21-7.17 (m, 2H), 4.06 (q, *J* = 7.2 Hz, 2H), 3.20 (t, *J* = 6.4 Hz, 2H), 2.62 (t, *J* = 6.4 Hz, 2H), 1.18 (t, *J* = 7.2 Hz, 3H); ^{13}C NMR (100 MHz, DMSO-*d6*) δ 192.1, 173.0, 139.2, 135.4, 126.0, 124.0, 123.4, 122.8, 117.2, 111.7, 60.3, 35.5, 28.8, 14.6; Anal. calcd for $C_{14}H_{14}ClNO_3$: C, 60.12; H, 5.05; N, 5.01. Found: C, 60.16; H, 4.84; N, 5.24.

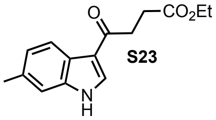

Ethyl 4-(6-chloro-1H-indole-3-yl)-4-oxobutanoate (S23) (1.02 g, 55%) was prepared according to the representative procedure from 6-chloro-indole (1.0 g, 6.6 mmol). Purification by silica gel chromatography (50% EA/ 50% hexanes) afforded **S23** as a brown solid. mp = 137-141 ºC; TLC R_f = 0.31 (hexanes: EA 50%:50%); IR (film, cm^{-1}) ν_{max} 3257, 3063, 2986, 1706, 1649, 1527; ^{1}H NMR (400 MHz, $CDCl_3$) δ 8.92 (bs, 1H), 8.27 (d, *J* = 8.6 Hz, 1H), 7.83 (d, *J* = 3.0 Hz, 1H), 7.40 (d, *J* = 1.5 Hz, 1H), 7.25 (dd, *J* = 8.6, 1.8 Hz, 1H), 4.20 (q, *J* = 7.2 Hz, 2H), 3.19 (t, *J* = 6.5 Hz, 2H), 2.81 (t, *J* = 6.5 Hz, 2H), 1.30 (t, *J* = 7.2 Hz, 3H); ^{13}C

NMR (100 MHz, $CDCl_3$) δ 193.5, 173.5, 136.6, 131.5, 129.6, 123.9, 123.3, 123.3, 117.6, 111.4, 60.8, 34.0, 28.3, 14.2; Anal. calcd for $C_{14}H_{14}ClNO_3$: C, 60.12; H, 5.05; N, 5.01. Found: C, 60.08; H, 4.98; N, 5.10.

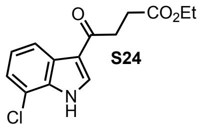

Ethyl 4-(7-chloro-1H-indole-3-yl)-4-oxobutanoate (S24) (0.87 g, 47%) was prepared according to the representative procedure from 7-chloro-indole (1.0 g, 6.6 mmol). Purification by silica gel chromatography (50% EA/ 50% hexanes) afforded **S24** as a yellow solid. mp = 146-149 °C; TLC R_f = 0.44 (hexanes: EA 50%:50%); IR (film, cm^{-1}) ν_{max} 3276, 3112, 2982, 1716, 1652, 1522, 1435; ^1H NMR (400 MHz, $CDCl_3$) δ 8.87 (bs, 1H), 8.29 (d, J = 7.9 Hz, 1H), 7.98 (d, J = 3.0 Hz, 1H), 7.24 (t, J = 7.8 Hz, 1H), 4.19 (q, J = 7.1 Hz, 2H), 3.25 (t, J = 6.8 Hz, 2H), 2.82 (t, J = 6.8 Hz, 2H), 1.29 (t, J = 7.1 Hz, 3H); ^{13}C NMR (100 MHz, $CDCl_3$) δ 193.5, 173.3, 133.6, 131.1, 126.8, 123.5, 123.1, 121.1, 118.7, 116.7, 60.7, 34.3, 28.3, 14.2; Anal. calcd for $C_{14}H_{14}ClNO_3$: C, 60.12; H, 5.05; N, 5.01. Found: C, 60.42; H, 5.05; N, 5.20.

General Procedure for the Ketone Reduction:[6]

In a flame dried round bottom flask equipped with a stir bar, 1 equiv. of substituted indole derivative was dissolved in 0.2 M of THF. Then, $NaBH_4$ (2 equiv.) and TFA (1 equiv.) was added slowly to the flask and the solution was stirred for 2 hours. Then, the reaction mixture was quenched with cold 1M NaOH and extracted three times with EA. The combined organic layers were washed with brine, dried over Na_2SO_4 and reduced under pressure. The residue was purified by silica gel chromatography.

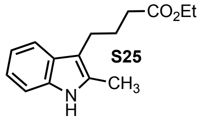

Ethyl 4-(2-methyl-1H-indole-3-yl)-butanoate (S25) (0.20 g, 30%) was prepared according to the representative procedure from **S14** (0.7 g, 2.7 mmol). Purification by silica gel chromatography (60% EA/ 40% hexanes) afforded **S25** as a yellow solid. mp = 68–71 °C; TLC R_f = 0.70 (hexanes: EA 40%:60%); IR (film, cm^{-1}) ν_{max} 3396, 2929, 1711, 1621, 1462; ^1H NMR (400 MHz, $CDCl_3$) δ 7.76 (bs, 1H), 7.52 (d, J = 7.0 Hz, 1H), 7.28 (dd, J = 6.8, 1.0 Hz, 1H), 7.11 (dp, J = 7.1, 1.3 Hz, 2H), 4.13 (q, J = 7.2 Hz, 2H), 2.77 (t, J = 7.4 Hz, 2H), 2.39 (s, 3H), 2.35 (t, J = 7.4 Hz, 2H), 1.99 (p, J = 7.4 Hz, 2H), 1.26 (t, J = 7.2 Hz, 3H); ^{13}C NMR (100 MHz, $CDCl_3$) δ 173.8, 135.3, 131.1, 128.7, 120.9, 119.1, 118.1, 111.0, 110.1, 60.2, 33.8, 25.7, 23.3, 14.2, 11.6; Anal. calcd for $C_{15}H_{19}NO_2$: C, 73.44; H, 7.81; N, 5.71. Found: C, 73.36; H, 7.83; N, 5.93.

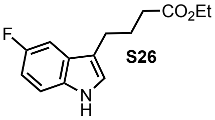

Ethyl 4-(5-fluoro-1H-indol-3-yl)butanoate (30) (0.36 g, 47%) was prepared according to the representative procedure from **29** (0.8 g, 3.04 mmol). Purification by silica gel chromatography (50% EA/ 50% hexanes) afforded **30** as a yellow solid. mp = 66–68 °C ; TLC R_f = 0.73 (hexanes: EA 50%:50%); IR (film, cm^{-1}) ν_{max} 3305, 2962, 1716, 1457; ^1H NMR (400 MHz, $CDCl_3$) δ 8.03 (s, 1H), 7.29-7.25 (m, 2H), 7.05 (d, J = 1.4 Hz, 1H), 6.95 (td, J = 9.0, 2.4 Hz, 1H), 4.15 (q, J = 7.1 Hz, 2H), 2.78 (t, J = 7.4 Hz, 2H), 2.40 (t, J = 7.4 Hz, 2H), 2.05 (pent, J = 7.4 Hz, 2H), 1.28 (t, J = 7.1 Hz, 3H); ^{13}C NMR (100MHz, $CDCl_3$) δ 173.7, 157.6 (d, J = 232.7 Hz), 132.8, 127.8 (d, J = 9.5 Hz), 123.3, 115.8 (d, J = 4.9 Hz), 111.6 (d, J = 10.0 Hz), 110.3 (d, J = 26.0 Hz), 103.8 (d, J = 23.2 Hz), 60.3, 33.9, 25.3, 24.4, 14.2; ^{19}F NMR (376.5 MHz, $CDCl_3$) δ -125.0 Anal. calcd for $C_{14}H_{16}FNO_2$: C, 67.45; H, 6.47; N, 5.62. Found: C, 67.58; H, 6.58; N, 5.75.

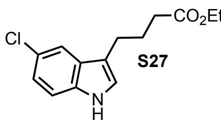

S27

Ethyl 4-(5-chloro-1H-indole-3-yl)-butanoate (34) (0.21 g, 27%) was prepared according to the representative procedure from **33** (0.8 g, 2.86 mmol). Purification by silica gel chromatography (50% EA/ 50% hexanes) afforded **34** as a brown solid. mp = 67–69 ºC; TLC R_f = 0.74 (hexanes: EA 50%:50%); IR (film, cm^{-1}) ν_{max} 3289, 2982, 2931, 1710, 1459, 1183; ^1H NMR (400 MHz, $CDCl_3$) δ 8.00 (bs, 1H), 7.58 (d, *J* = 1.8 Hz, 1H), 7.29 (d, *J* = 8.5 Hz, 1H), 7.16 (dd, *J* = 8.6, 1.9 Hz, 1H), 7.04 (d, *J* = 1.8 Hz, 1H), 4.14 (q, *J* = 7.2 Hz, 2H), 2.78 (t, *J* = 7.4 Hz, 2H), 2.39 (t, *J* = 7.4 Hz, 2H), 2.04 (p, *J* = 7.4 Hz, 2H), 1.28 (t, *J* = 7.2 Hz, 3H); ^{13}C NMR (100 MHz, $CDCl_3$) δ 173.6, 134.7, 128.6, 125.0, 122.8, 122.3, 118.4, 115.6, 112.0, 60.3, 33.9, 25.3, 24.3, 14.2; Anal. calcd for $C_{14}H_{16}ClNO_2$: C, 63.28; H, 6.07; N, 5.27. Found: C, 63.41; H, 6.08; N, 5.14.

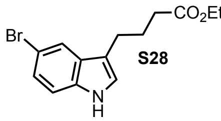

S28

Ethyl 4-(5-bromo-1H-indol-3-yl)butanoate (38) (0.20 g, 29%) was prepared according to the representative procedure from **37** (0.7 g, 2.16 mmol). Purification by silica gel chromatography (60% EA/ 40% hexanes) afforded **38** as a yellow solid. mp = 69–73 ºC; TLC R_f = 0.73 (hexanes: EA 50%:50%); mp = 67 – 69 ºC ; TLC R_f = 0.35 (hexanes : EA 70%:30%); IR (film, cm^{-1}) ν_{max} 3283, 2980, 2904, 2839, 1707, 1617, 1437; ^1H NMR (400 MHz, $CDCl_3$) δ 8.01 (s, 1H), 7.74 (s, 1H), 7.25 (t, *J* = 8.6 Hz, 2H), 7.02 (d, *J* = 2.0 Hz, 1H), 4.15 (q, *J* = 7.1 Hz, 2H), 2.78 (t, *J* = 7.4 Hz, 2H), 2.39 (t, *J* = 7.4 Hz, 2H), 2.04 (pent, *J* = 7.4 Hz, 2H), 1.28 (t, *J* = 7.1 Hz, 3H); ^{13}C NMR (100 MHz, $CDCl_3$) δ 173.6, 134.9, 129.3, 124.8, 122.7, 121.5, 115.5, 112.5, 112.5, 60.3, 33.9, 25.3, 24.3, 14.3; Anal. calcd for $C_{14}H_{16}BrNO_2$: C, 54.21; H, 5.20; N, 4.52. Found: C, 54.05; H, 5.23; N, 4.38.

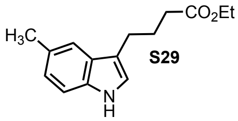

S29

Ethyl 4-(5-methyl-1H-indol-3-yl)butanoate (S29) (0.33 g, 50%) was prepared according to the representative procedure from **41** (0.7 g, 2.7 mmol). Purification by silica gel chromatography (60% hexanes/ 40% EA) afforded **42** as a white solid. mp = 49–52 ºC; TLC R_f = 0.59 (hexanes: EA 60%:40%); IR (film, cm^{-1}) ν_{max} 3324, 2992, 2938, 2864, 1715, 1463, 1164; ^1H NMR (400 MHz, $CDCl_3$) δ 7.84 (bs, 1H), 7.38 (s, 1H), 7.24 (d, *J* = 8.3 Hz, 1H), 7.02 (dd, *J* = 8.2, 0.7 Hz, 1H), 6.95 (s, 1H), 4.13 (q, *J* = 7.1 Hz, 2H), 2.78 (t, *J* = 7.4 Hz, 2H), 2.46 (s, 3H), 2.38 (t, *J* = 7.4 Hz, 2H), 2.04 (pent, *J* = 7.4 Hz, 2H), 1.25 (t, *J* = 7.1 Hz, 3H); ^{13}C NMR (100 MHz, $CDCl_3$) δ 173.8, 134.7, 128.4, 127.7, 123.5, 121.6, 118.6, 115.2, 110.7, 60.2, 34.0, 25.4, 24.5, 21.5, 14.3; Anal. calcd for $C_{15}H_{19}NO_2$: C, 73.44; H, 7.81; N, 5.71. Found: C, 73.76; H, 7.59; N, 5.40.

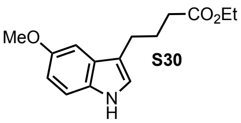

S30

Ethyl 4-(5-methoxy-1H-indol-3-yl)butanoate (S30) (0.28 g, 42%) was prepared according to the representative procedure from **45** (0.7 g, 2.5 mmol). Purification by silica gel chromatography (50% EA/ 50% hexanes) afforded **46** as a white solid. mp = 69–71 ºC ; TLC R_f = 0.77 (hexanes: EA 50%:50%); IR (film, cm^{-1}) ν_{max} 3377, 2980, 2922, 1716, 1626, 1487; ^1H NMR (400 MHz, $CDCl_3$) δ 7.87 (bs, 1H), 7.27 (d, *J* = 8.9 Hz, 1H), 7.07 (d, *J* = 2.3Hz, 1H), 7.0 (s, 1H), 6.88 (dd, *J* = 8.8, 4.8 Hz, 1H), 4.15 (q, *J* = 7.1 Hz, 2H), 3.90 (s, 3H), 2.80 (t, *J* = 7.4 Hz, 2H), 2.41 (t, *J* = 7.4 Hz, 2H), 2.06 (pent, *J* = 7.4 Hz, 2H), 1.27 (t, *J* = 7.1 Hz, 3H); ^{13}C NMR (100 MHz, $CDCl_3$) δ 173.8, 153.9, 131.5, 127.8, 122.3, 115.4, 112.1, 111.8, 100.8, 60.2, 56.0, 34.0, 25.2, 24.5, 14.3; Anal. calcd for $C_{15}H_{19}NO_3$: C, 68.94; H, 7.33; N, 5.36. Found: C, 68.80; H, 7.52; N, 5.67.

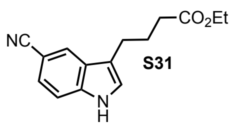

S31

Ethyl 4-(5-cyano-1H-indol-3-yl)butanoate (S31) (0.063 g, 12%) was prepared according to the representative procedure from **49** (0.56 g, 2.1 mmol). Purification by silica gel chromatography (70% EA/ 30% hexanes) afforded **50** as a white solid. mp = 64–67 °C ; TLC R_f = 0.71 (hexanes: EA 30%:70%); IR (film, cm^{-1}) ν_{max} 3261, 2978, 2214, 1728, 1617, 1444; ^1H NMR (400 MHz, $CDCl_3$) δ 8.30 (s, 1H), 7.98 (s, 1H), 7.46-7.41 (m, 2H), 7.14 (d, *J* = 2.0 Hz, 1H), 4.15 (q, *J* = 7.1 Hz, 2H), 2.83 (t, *J* = 7.4 Hz, 2H), 2.40 (t, *J* = 7.4 Hz, 2H), 2.06 (pent, *J* = 7.4 Hz, 2H), 1.28 (t, *J* = 7.1 Hz, 3H); ^{13}C NMR (100 MHz, $CDCl_3$) δ 173.4, 137.9, 127.4, 125, 124.7, 123.5, 120.8, 116.8, 111.9, 102.5, 60.4, 33.8, 25.3, 24.2, 14.2; Anal. calcd for $C_{15}H_{16}N_2O_2$: C, 70.29; H, 6.29; N, 10.93. Found: C, 70.15; H, 6.36; N, 10.83.

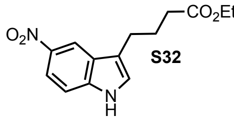

S32

Ethyl 4-(5-nitro-1H-indol-3-yl)butanoate (S32) (0.18 g, 21%) was prepared according to the representative procedure from **53** (0.9 g, 3.1 mmol). Purification by silica gel chromatography (60% EA/ 40% hexanes) afforded **54** as a yellow solid. mp = 102–105 °C ; TLC R_f = 0.64 (hexanes: EA 40%:60%); IR (film, cm^{-1}) ν_{max} 3301, 2978, 2943, 1703, 1623, 1512; ^1H NMR (400 MHz, $CDCl_3$) δ 8.57 (d, *J* = 2.1 Hz, 1H), 8.42 (bs, 1H), 8.10 (dd, *J* = 9.0, 2.2 Hz, 1H), 7.38 (d, *J* = 9.0 Hz, 1H), 7.15 (d, *J* = 1.9 Hz, 1H), 4.14 (q, *J* = 7.1 Hz, 2H), 2.84 (t, *J* = 7.5 Hz, 2H), 2.39 (t, *J* = 7.5 Hz, 2H), 2.05 (pent, *J* = 7.5 Hz, 2H), 1.26 (t, *J* = 7.1 Hz, 3H); ^{13}C NMR (100 MHz, $CDCl_3$) δ 173.4, 141.6, 139.3, 127.0, 124.4, 118.5, 117.8, 116.3, 111.0, 60.4, 33.8, 25.3, 24.2, 14.2; Anal. calcd for $C_{14}H_{16}N_2O_4$: C, 60.86; H, 5.84; N, 10.14. Found: C, 60.72; H, 5.83; N, 10.14.

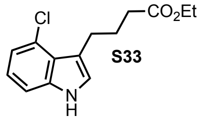

S33

Ethyl 4-(4-chloro-1H-indole-3-yl)-butanoate (S33) (0.16 g, 26%) was prepared according to the representative procedure from **61** (0.65 g, 2.3 mmol). Purification by silica gel chromatography (50% EA/ 50% hexanes) afforded **62** as a brown solid. mp = 63–65 °C; TLC R_f = 0.69 (hexanes: EA 50%:50%); IR (film, cm^{-1}) ν_{max} 3326, 2982, 2947, 1703, 1617, 1431; ^1H NMR (400 MHz, $CDCl_3$) δ 8.06 (bs, 1H), 7.25 (q, *J* = 4.3 Hz, 1H), 7.09 (s, 1H), 7.07 (d, *J* = 1.0 Hz, 1H), 7.03 (d, *J* = 2.0 Hz, 1H), 4.13 (q, *J* = 7.1 Hz, 2H), 3.05 (t, *J* = 7.5 Hz, 2H), 2.42 (t, *J* = 7.5 Hz, 2H), 2.09 (p, *J* = 7.5 Hz, 2H), 1.27 (t, *J* = 7.1 Hz, 3H); ^{13}C NMR (100 MHz, $CDCl_3$) δ 173.8, 137.9, 126.5, 124.2, 123.0, 122.5, 120.4, 116.3, 109.8, 60.2, 33.9, 26.7, 25.6, 14.3; Anal. calcd for $C_{14}H_{16}ClNO_2$: C, 63.28; H, 6.07; N, 5.27. Found: C, 63.56; H, 6.18; N, 5.44.

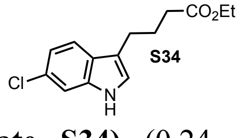

S34

Ethyl 4-(6-chloro-1H-indole-3-yl)-butanoate S34) (0.24 g, 25%) was prepared according to the representative procedure from **65** (1.0 g, 3.6 mmol). Purification by silica gel chromatography (50% EA/ 50% hexanes) afforded **66** as a brown solid. mp = 85–87 °C; TLC R_f = 0.74 (hexanes: EA 50%:50%); IR (film, cm^{-1}) ν_{max} 3310, 2980, 2925, 1709, 1622, 1549; ^1H NMR (400 MHz, $CDCl_3$) δ 7.97 (bs, 1H), 7.52 (d, *J* = 8.5 Hz, 1H), 7.36 (d, *J* = 1.5 Hz, 1H), 7.10 (dd, *J* = 8.5, 1.8 Hz, 1H), 7.00 (d, *J* = 1.9 Hz, 1H), 4.14 (q, *J* = 7.2 Hz, 2H), 2.80 (t, *J* = 7.5 Hz, 2H), 2.39 (t, *J* = 7.5 Hz, 2H), 2.05 (p, *J* = 7.5 Hz, 2H), 1.27 (t, *J* = 7.2 Hz, 3H); ^{13}C NMR (100 MHz, $CDCl_3$) δ 173.7, 136.7, 127.9, 126.1, 122.0, 120.0, 119.8, 115.9, 111.0, 60.3, 33.9, 25.3, 24.4, 14.2; Anal calcd for $C_{14}H_{16}ClNO_2$: C, 63.28; H, 6.07; N, 5.27. Found: C, 63.36; H, 5.88; N, 5.42.

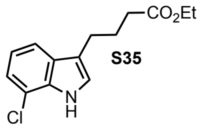

Ethyl 4-(7-chloro-1H-indole-3-yl)-butanoate (S35) (0.23 g, 31%) was prepared according to the representative procedure from **69** (0.79 g, 2.8 mmol). Purification by silica gel chromatography (30% EA/ 70% hexanes) afforded **70** as a white solid. mp = 74-66 °C; TLC R_f= 0.59 (70% hexanes: 30% EA); IR (film, cm^{-1}) ν_{max} 3397, 2978, 1719, 1622, 1494; ^{1}H NMR (400 MHz, DMSO-d_6) δ 11.2 (bs, 1H), 7.50 (d, *J* = 7.9 Hz, 1H), 7.20 (d, *J* = 2.2 Hz, 1H), 7.15 (d, *J* = 7.5 Hz, 1H), 7.00 (t, *J* = 7.7 Hz, 1H), 4.04 (q, *J* = 7.1 Hz, 2H), 2.71 (t, *J* = 7.5 Hz, 2H), 2.33 (t, *J* = 7.5 Hz, 2H), 1.89 (p, *J* = 7.5Hz, 2H), 1.17 (t, *J* = 7.1 Hz, 3H); ^{13}C NMR (100 MHz, DMSO-d_6) δ 173.3, 133.5, 129.6, 124.3, 120.9, 119.7, 117.9, 116.3, 115.7, 60.1, 33.6, 25.7, 24.3, 14.6; Anal. calcd for $C_{14}H_{16}ClNO_2$: C, 63.28; H, 6.07; N, 5.27. Found: C, 63.58; H, 6.11; N, 5.43.

General Procedure for Ester Hydrolysis:

In a round bottom flask equipped with stir bar, 1 equiv. of substituted indole-derivative was dissolved in methanol (0.4 M). Then, 3 equiv. of KOH was added, and the reaction mixture was heated to reflux (80 °C oil bath temperature). After two hours, the reaction mixture was quenched with HCl and transferred to a separatory funnel. The aqueous layer was extracted three times with EA. The combined organic layers were washed with brine, dried (Na_2SO_4) and concentrated to afford the desired acids.

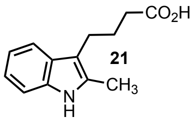

4-(2-Methyl-1H-indol-3-yl)butanoic acid (21) (0.24 g, 92%) was prepared according to the representative procedure from **58** (0.30 g, 1.2 mmol). Brown solid, mp = 86–89 °C; TLC R_f= 0.38 (hexanes : EA 60%:40%); IR (film, cm^{-1}) ν_{max} 3387, 2960, 1702, 1459; ^{1}H NMR (400 MHz, $CDCl_3$) δ 7.65 (bs, 1H), 7.42 (d, *J* = 7.0 Hz, 1H), 7.20-7.18 (m, 1H), 7.05-6.98 (m, 2H) 2.69 (t, *J* = 7.3 Hz, 2H), 2.32-2.29 (m, 5H), 1.91 (pent, *J* = 7.3 Hz, 2H); ^{13}C NMR (100 MHz, DMSO-d_6) δ 175.0, 135.6, 132.0, 128.7, 120.3, 118.5, 117.8, 110.8, 110.0, 33.6, 26.2, 23.3, 11.6; Anal. calcd for $C_{13}H_{15}NO_2$: C, 71.87; H, 6.96; N, 6.45. Found: C, 71.87; H, 6.95; N, 6.37.

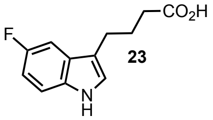

Ethyl 4-(5-fluoro-1H-indol-3-yl)butanoic acid (23) (0.17 g, 95%) was prepared according to the representative procedure from **30** (0.202 g, 0.81 mmol). brown solid, mp = 124–127 °C ; TLC R_f= 0.29 (EA/H 50%/50%); IR (film, cm^{-1}) ν_{max} 3381, 2929, 1690, 1582, 1486; ^{1}H NMR (400 MHz, DMSO-d_6) δ 12.0 (bs, 1H), 10.9 (s, 1H), 7.32 (dd, *J* = 8.8, 4.6 Hz, 1H), 7.25 (dd, *J* = 10.0, 2.5 Hz, 1H), 7.19 (d, *J* = 2.1 Hz, 1H), 6.90 (td, *J* = 9.2, 2.5Hz, 1H), 2.66 (t, *J* = 7.5 Hz, 2H), 2.26 (t, *J* = 7.5 Hz, 2H), 1.84 (pent, *J* = 7.5 Hz, 2H); ^{13}C NMR (100 MHz, DMSO-d_6) δ 175.0, 157.0 (d, *J* = 231.0 Hz), 133.4, 127.8 (d, *J* = 9.6 Hz), 125.0, 114.7 (d, *J* = 4.9 Hz), 112.7 (d, *J* = 9.8 Hz), 109.4 (d, *J* = 26.1 Hz), 103.4 (*J* = 22.8 Hz), 33.8, 25.8, 24.4; ^{19}F NMR (376.5 MHz, DMSO-d_6) δ -125,7; Anal. calcd for $C_{12}H_{12}FNO_2$: C, 65.15; H, 5.47; N, 6.33. Found: C, 64.95; H, 5.44; N, 6.43.

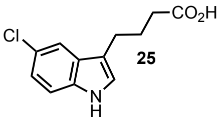

4-(5-Chloro-1H-indol-3-yl)butanoic acid (25) (0.16 g, 88%) was prepared according to the representative procedure from **34** (0.20 g, 0.75 mmol). Orange solid, mp = 141–143 °C ; TLC R_f= 0.30 (hexanes : EA 80%:20%); IR (film, cm^{-1}) ν_{max} 3383, 2993, 2925, 1696, 1436, 1203; ^{1}H NMR (400 MHz, DMSO-d_6) δ 12.03 (bs, 1H), 11.0 (s, 1H), 7.54 (d, *J* = 1.8 Hz, 1H), 7.35 (d, *J* = 8.5 Hz, 1H), 7.20 (d, *J* = 2.0 Hz, 1H), 7.05 (dd, *J*

= 8.6, 2.0 Hz, 1H), 2.67 (t, J = 7.5 Hz, 2H), 2.26 (t, J = 7.5 Hz, 2H), 1.84 (pent, J = 7.5 Hz, 2H); ^{13}C NMR (100 MHz, DMSO-d_6) δ 174.9, 135.2, 128.8, 124.7, 123.3, 121.2, 118.0, 114.4, 113.3, 33.8, 25.8, 24.3; Anal. calcd for $C_{12}H_{12}ClNO_2$: C, 60.64; H, 5.09; N, 5.89. Found: C, 60.94; H, 5.36; N, 6.03.

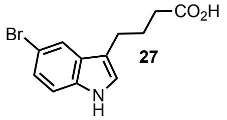

27

4-(5-Bromo-1H-indol-3-yl)butanoic acid (27) (0.14 g, 83%) was prepared according to the representative procedure from **38** (0.18 g, 0.58 mmol). Brown solid, mp = 142–144 ºC; TLC R_f = 0.32 (hexanes : EA 10%:90%); IR (film, cm^{-1}) ν_{max} 3382, 3120, 2924, 2701, 1695, 1568, 1457; ^{1}H NMR (400 MHz, DMSO-d_6) δ 12.1 (s, 1H), 11.0 (s, 1H), 7.68 (d, J = 1.6 Hz, 1H), 7.31 (d, J = 8.6 Hz, 1H), 7.19-7.15 (m, 2H), 2.67 (t, J = 7.5 Hz, 2H), 2.26 (t, J = 7.5 Hz, 2H), 1.83 (pent, J = 7.5 Hz, 2H); ^{13}C NMR (100 MHz, DMSO-d_6) δ 174.9, 135.4, 129.5, 124.5, 123.7, 121.0, 114.4, 113.8, 111.3, 33.9, 25.9, 24.3; Anal. calcd for $C_{12}H_{12}BrNO_2$: C, 51.09; H, 4.29; N, 4.96. Found: C, 51.01; H, 4.23; N, 4.91.

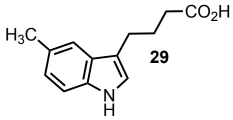

29

4-(5-Methyl-1H-indol-3-yl)butanoic acid (29) (0.21 g, 80%) was prepared according to the representative procedure from **42** (0.3 g, 1.23 mmol). orange solid, mp = 152–155 ºC ; TLC R_f = 0.35 (hexanes : EA 40%:60%); IR (film, cm^{-1}) ν_{max} 3392, 2922, 2867, 1690, 1431; ^{1}H NMR (400 MHz, acetone-d_6) δ 9.82 (bs, 1H), 7.38 (d, J = 0.5 Hz, 1H), 7.26 (d, J = 8.3 Hz, 1H), 7.10 (s, 1H), 6.93 (dd, J = 8.2, 0.7 Hz, 1H), 2.80 (t, J = 7.5 Hz, 2H), 2.41 (s, 3H), 2.38 (t, J = 7.5 Hz, 2H), 2.00 (pent, J = 7.5 Hz, 2H); ^{13}C NMR (100 MHz, acetone-d_6) δ 173.8, 135.1, 127.9, 127.1, 122.7, 121.9, 118.1, 114.2, 110.9, 32.9, 25.6, 24.3, 20.7; Anal. calcd for $C_{13}H_{15}NO_2$: C, 71.87; H, 6.96; N, 6.45. Found: C, 71.99; H, 6.97; N, 6.64.

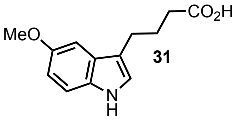

31

Ethyl 4-(5-methoxy-1H-indol-3-yl)butanoic acid (31) (0.20 g, 90%) was prepared according to the representative procedure from **46** (0.22 g, 0.96 mmol). Brown solid; TLC R_f = 0.21 (H/EA 50%/50%); IR (film, cm^{-1}) ν_{max} 3331, 3129, 2945, 1705, 1612, 1469, 1219; ^{1}H NMR (400 MHz, DMSO-d_6) δ 12.0 (bs, 1H), 10.6 (s, 1H), 7.22 (d, J = 8.8 Hz, 1H), 7.06 (d, J = 2.0 Hz, 1H), 7.00 (d, J = 2.3 Hz, 1H), 6.7 (dd, J = 8.7, 2.4 Hz, 1H), 3.76 (s, 3H), 2.66 (t, J = 7.3 Hz, 2H), 2.27 (t, J = 7.3 Hz, 2H), 1.86 (pent, J = 7.3 Hz, 2H); ^{13}C NMR (100 MHz, DMSO-d_6) δ 175.0, 153.3, 131.9, 127.9, 123.4, 114.1, 112.4, 111.4, 100.6, 55.8, 33.8, 25.7, 24.5. This compound has been previously prepared.[9]

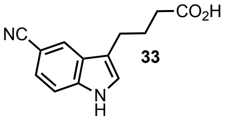

33

Ethyl 4-(5-cyano-1H-indol-3-yl)butanoic acid (33) (0.047 g, 82%) was prepared according to the representative procedure from **50** (0.063 g, 0.25 mmol). yellow solid, mp = 155–158 ºC ; TLC R_f = 0.2 (EA 100%); IR (film, cm^{-1}) ν_{max} 3298, 3142, 2937, 2224, 1705, 1616, 1474, 1221; ^{1}H NMR (400 MHz, DMSO-d_6) δ 12.0 (bs, 1H), 11.4 (s, 1H), 8.08 (s, 1H), 7.50 (d, J = 8.5 Hz, 1H), 7.41 (dd, J = 8.5, 1.5 Hz, 1H), 7.34 (d, J = 1.9 Hz, 1H), 2.73 (t, J = 7.3 Hz, 2H), 2.27 (t, J = 7.3 Hz, 2H), 1.86 (pent, J = 7.3 Hz, 2H); ^{13}C NMR (100 MHz, DMSO-d_6) δ 174.9, 138.4, 127.5, 125.5, 124.6, 124.1, 121.4, 115.8, 113.1, 100.7, 33.8, 25.8, 24.1; Anal. calcd for $C_{13}H_{12}N_2O_2$: C, 68.41; H, 5.30; N, 12.27. Found: C, 68.10; H, 5.06; N, 11.91.

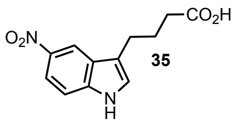

35

Ethyl 4-(5-nitro-1H-indol-3-yl)butanoic acid (35) (0.11 g, 70%) was prepared according to the representative procedure from **54** (0.17 g, 0.61 mmol). yellow solid, mp = 167–169 °C ; TLC R_f = 0.25 (EA/H 60%/40%); IR (film, cm^{-1}) ν_{max} 3367, 2936, 1682, 1621, 1514; ^1H NMR (400 MHz, CD_3OD) δ 8.57 (d, *J* = 2.1 Hz, 1H), 8.05 (dd, *J* = 9.0, 2.2 Hz, 1H), 7.46 (d, *J* = 9.0 Hz, 1H), 7.28 (s, 1H), 2.88 (t, *J* = 7.3 Hz, 2H), 2.39 (t, *J* = 7.3 Hz, 2H), 2.04 (pent, *J* = 7.3 Hz, 2H); ^{13}C NMR (100 MHz, acetone-d_6) δ 173.6, 141.1, 139.8, 127.0, 125.9, 117.8, 116.6, 115.6, 111.5, 32.8, 25.6, 23.9; Anal. calcd for $C_{12}H_{12}N_2O_4$: C, 58.06; H, 4.87; N, 11.29. Found: C, 58.28; H, 5.07; N, 11.18.

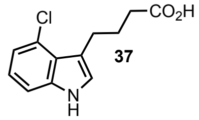

37

4-(4-Chloro-1H-indol-3-yl)butanoic acid (37) (0.074 g, 57%) was prepared according to the representative procedure from **62** (0.14 g, 0.54 mmol). Yellow solid, mp = 134–136 °C ; TLC R_f = 0.25 (hexanes : EA 50%:50%); IR (film, cm^{-1}) ν_{max} 3436, 2912, 1685, 1428; ^1H NMR (400 MHz, CD_3OD) δ 7.27 (dd, *J* = 7.8, 0.9 Hz, 1H), 7.09 (s, 1H), 7.01 (t, *J* = 7.7 Hz, 1H), 6.96 (dd, *J* = 7.5, 0.9 Hz, 1H), 3.02 (t, *J* = 7.5 Hz, 2H), 2.37 (t, *J* = 7.5 Hz, 2H), 2.02 (pent, *J* = 7.5 Hz, 2H); ^{13}C NMR (100 MHz, DMSO-d_6) δ 174.9, 138.5, 125.1, 125.0, 123.8, 122.1, 119.5, 114.5, 111.2, 33.8, 27.1, 25.7; Anal. calcd for $C_{12}H_{12}ClNO_2$: C, 60.64; H, 5.09; N, 5.89. Found: C, 60.38; H, 5.04; N, 6.01.

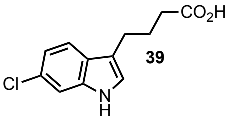

39

4-(6-Chloro-1H-indol-3-yl)butanoic acid (39) (0.14 g, 78%) was prepared according to the representative procedure from **66** (0.20 g, 0.75 mmol). Orange solid, mp = 151–154 °C ; TLC R_f = 0.25 (hexanes : EA 50%:50%); IR (film, cm^{-1}) ν_{max} 3404, 2936, 1695, 1456; ^1H NMR (400 MHz, CD_3OD) δ 7.50 (d, *J* = 8.5 Hz, 1H), 7.34 (d, *J* = 1.5 Hz, 1H), 7.06 (s, 1H), 6.98 (dd, *J* = 8.4, 1.9 Hz, 1H), 2.79 (t, *J* = 7.4 Hz, 2H), 2.36 (t, *J* = 7.4 Hz, 2H), 1.99 (pent, *J* = 7.4 Hz, 2H); ^{13}C NMR (100 MHz, CD_3OD) δ 176.2, 137.1, 126.7, 126.1, 122.5, 119.0, 118.5, 114.6, 110.5, 33.1, 25.5, 23.9; Anal. calcd for $C_{12}H_{12}ClNO_2$: C, 60.64; H, 5.09; N, 5.89. Found: C, 60.54; H, 4.91; N, 5.85.

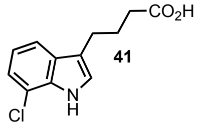

41

4-(7-Chloro-1H-indol-3-yl)butanoic acid (41) (0.14 g, 75%) was prepared according to the representative procedure from **70** (0.21 g, 0.8 mmol). Brown solid, mp = 136–138 °C; TLC R_f = 0.41 (hexanes : EA 50%:50%); IR (film, cm^{-1}) ν_{max} 3393, 2916, 1686, 1430; ^1H NMR (400 MHz , DMSO-d_6) δ 12.0 (s, 1H), 11.1 (s, 1H), 7.50 (d, *J* = 7.9 Hz, 1H), 7.20 (d, *J* = 2.1 Hz, 1H), 7.15 (d, *J* = 7.4 Hz, 1H), 6.99 (d, *J* = 7.7 Hz, 1H), 2.71 (t, *J* = 7.5 Hz, 2H), 2.26 (t, *J* = 7.5 Hz, 2H), 1.87 (pent, *J* = 7.5 Hz, 2H); ^{13}C NMR (100 MHz, DMSO-d_6) δ 174.9, 133.5, 129.6, 124.2, 120.9, 119.7, 117.9, 116.2, 115.8, 33.8, 25.7, 24.5; Anal. calcd for $C_{12}H_{12}ClNO_2$: C, 60.64; H, 5.09; N, 5.89. Found: C, 60.84; H, 5.29; N, 5.85.

General Procedure for the catalytic oxidative lactonization with DDQ:

In a round bottom flask equipped with stir bar, 1 equiv. of substituted indole butyric acid was dissolved in 0.2 M of THF. Then, 10 mol% of DDQ and 5 equiv of MnO_2 were added all at once and the solution was let it stir overnight at room temperature. The reaction mixture was then diluted with DCM and filtrated in order to remove MnO_2. Then the solution was transferred to separatory funnel which was extracted three times with

$NaHCO_3$. The combined organic layers were washed with brine, dried with sodium sulfate and concentrated. Purification by silica gel chromatography afforded the desired lactones.

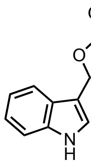
12

5-(1H-Indol-3-yl)oxolan-2-one (12) (0.075 g, 76%) was prepared according to the representative procedure from **11** (0.1 g, 0.49 mmol). Purification by silica gel chromatography (60% EA/ 40% hexanes) afforded **12** as a red solid. mp = 117–121 °C; TLC R_f = 0.66 (hexanes : EA 20%:80%); IR (film, cm^{-1}) ν_{max} 3417, 3056, 2909, 1752, 1423, 1189; ^1H NMR (400 MHz, $CDCl_3$,) δ 8.23 (s, 1H), 7.62 (d, *J* = 7.9 Hz, 1H), 7.39 (d, *J* = 8.1 Hz, 1H), 7.23-7.02 (m, 2H), 7.15 (dt, *J* = 8.0, 0.9 Hz, 1H), 5.88-5.82 (m, 1H), 2.72-2.61 (m, 3H) 2.56 – 2.45 (m, 1H); ^{13}C NMR (100 MHz, $CDCl_3$) δ 177.3, 136.7, 125.5, 122.9, 122.0, 120.4, 119.2, 114.5, 111.6, 31.0, 29.3, 28.9; Anal. calcd for $C_{12}H_{11}NO_2$: C, 71.63; H, 5.51; N, 6.96. Found: C, 71.90; H, 5.18; N, 7.22.

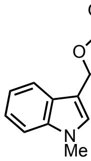
14

5-(1-Methyl-1H-indol-3-yl)oxolan-2-one (14) (0.034 g, 92%) was prepared according to the representative procedure from **13** (0.036 g, 0.17 mmol). Purification by silica gel chromatography (80% EA/ 20% hexanes) afforded **14** as a yellow solid. mp = 96–98 °C; TLC R_f = 0.69 (hexanes : EA 20%:80%); IR (film, cm^{-1}) ν_{max} 3117, 3073, 2919, 1742, 1616, 1476, 799; ^1H NMR (DMSO-*d*6, 300 MHz) δ 7.58 (d, *J* = 7.9 Hz, 1H), 7.48 (s, 1H), 7.44 (d, *J* = 8.2 Hz, 1H), 7.19 (t, *J* = 7.0 Hz, 1H), 7.07 (t, *J* = 7.8 Hz, 1H), 5.81 (dd, *J* = 8.5 Hz, *J* = 6.8 Hz, 1H), 3.76 (s, 3H) 2.70–2.47 (m, 4H); ^{13}C NMR (75 MHz, DMSO-*d*6) δ 177.6, 137.5, 128.8, 126.5, 122.2, 119.8, 119.4, 112.3, 110.6, 76.5, 33.0, 29.8, 28.5; Anal. calcd for $C_{13}H_{13}NO_2$: C, 72.50; H, 6.09; N, 6.51. Found: C, 72.76; H, 5.87; N, 6.13.

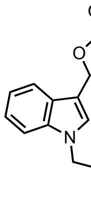
16

5-(1-Allyl-1H-indol-3-yl)oxolan-2-one (16) (0.0073 g, 39%) was prepared according to the representative procedure from **15** (0.019 g, 0.078 mmol). Purification by silica gel chromatography (50% EA/ 50% hexanes) afforded **16** as a yellow oil. TLC R_f = 0.62 (hexanes : EA 50%:50%); IR (film, cm^{-1}) ν_{max} 3051, 2983, 2920, 1757, 1555, 1467; ^1H NMR (400 MHz, $CDCl_3$) δ 7.56 (d, *J* = 7.9 Hz, 1H), 7.26 (d, *J* = 8.3 Hz, 1H), 7.18 (dt, *J* = 5.3, 0.8 Hz, 1H), 7.09 (d, *J* = 7.6 Hz, 1H), 7.06 (s, 1H), 5.96-5.86 (m, 1H), 5.81 – 5.77 (m, 1H), 5.16 (dd, *J* = 10.2, 1.2 Hz, 1H), 5.05 (dd, *J* = 17.1, 1.1 Hz, 1H), 4.64 (d, *J* = 5.5 Hz, 2H), 2.66 – 2.39 (m, 4H); ^{13}C NMR (100 MHz, $CDCl_3$) δ 177.2, 136.9, 133.0, 126.1, 125.5, 122.4, 120.0, 119.3, 117.8, 113.2, 110.1, 76.9, 48.9, 29.3, 29.0; Anal. calcd for $C_{15}H_{15}NO_2$: C, 74.67; H, 6.27; N, 5.81. Found: C, 74.46; H, 5.96; N, 6.06.

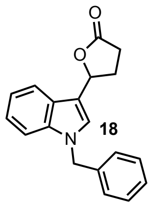

18

<u>5-(1-Benzyl-1H-indol-3-yl)oxolan-2-one (18)</u> (0.0057 g, 44%) was prepared according to the representative procedure from **17** (0.013 g, 0.046 mmol). Purification by silica gel chromatography (60% EA/ 40% hexanes) afforded **18** as a white solid. mp = 165–168 ºC; TLC R_f = 0.52 (hexanes: ethyl acetate 50%:50%); IR (film, cm^{-1}) ν_{max} 3086, 2980, 2909, 1756, 1614, 1557, 1495, 773; ^{1}H NMR (400 MHz, $CDCl_3$) δ 7.67 (d, *J* = 7.8 Hz, 1H), 7.35-7.30 (m, 4H), 7.25 (td, *J* = 7.1, 1.0 Hz, 1H), 7.20-7.14 (m, 4H), 5.91-5.88 (m, 1H), 5.32 (s, 2H), 2.78 – 2.49 (m, 4H); ^{13}C NMR (100 MHz, $CDCl_3$) δ 177.1, 137.1, 136.9, 128.9, 127.8, 126.9, 126.1, 125.9, 122.6, 120.1, 119.4, 113.5, 110.2, 76.8, 50.2, 29.3, 28.9; Anal. calcd for $C_{19}H_{17}NO_2$: C, 78.33; H, 5.88; N, 4.81. Found: C, 78.02; H, 5.48; N, 4.75.

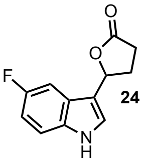

24

<u>5-(5-Fluoro-1H-indol-3-yl)oxolan-2-one (24)</u> (0.019 g, 19%) was prepared according to the representative procedure from **31** (0.1 g, 0.45 mmol). Purification by silica gel chromatography (60% EA/ 40% hexanes) afforded **32** as a red solid. mp = 133–136 ºC; TLC R_f = 0.29 (hexanes : EA 50%:50%); IR (film, cm^{-1}) ν_{max} 3417, 3121, 2921, 1746, 1580, 1483; ^{1}H NMR (400 MHz, acetone-*d6*) δ 10.48 (bs, 1H), 7.57 (d, *J* = 2.2 Hz, 1H), 7.46 (dd, *J* = 8.9, 4.5 Hz, 1H), 7.38 (dd, *J* = 9.9, 2.4 Hz, 1H), 6.97(td, *J* = 9.1, 2.5 Hz, 1H), 5.88-5.84 (m, 1H) 2.77-2.53 (m, 4H); ^{13}C NMR (100 MHz, DMSO-*d6*) 177.6, 157.4 (d, *J* = 232.5 Hz), 133.7, 126.7, 126.4 (d, *J* = 10.0 Hz), 113.4, (d, *J* = 5.0 Hz), 113.3 (d, *J* = 9.9 Hz), 110.3 (d, *J* = 26.1 Hz), 104.0 (d, *J* = 23.5 Hz), 76.1, 29.8, 28.3; ^{19}F NMR (376.5 MHz, DMSO-*d6*) δ -124.4; Anal. calcd for $C_{12}H_{10}FNO_2$: C, 65.75; H, 4.60; N, 6.39. Found: C, 65.81; H, 4.66; N, 6.44.

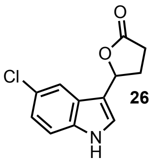

26

<u>5-(5-Chloro-1H-indol-3-yl)oxolan-2-one (26)</u> (0.062 g, 62%) was prepared according to the representative procedure from **35** (0.1 g, 0.42 mmol). Purification by silica gel chromatography (80% EA/ 20% hexanes) afforded **36** as a purple solid. mp = 149–152 ºC; TLC R_f = 0.53 (hexanes : EA 20%:80%); IR (film, cm^{-1}) ν_{max} 3372, 3124, 3078, 2930, 1743, 1457; ^{1}H NMR (400 MHz, CD_3OD) δ 7.60 (d, *J* = 1.8 Hz, 1H), 7.43 (s, 1H), 7.38 (d, *J* = 8.7 Hz, 1H), 7.14 (dd, *J* = 8.7, 2.0 Hz, 1H), 5.88 (dd, *J* = 8.6, 6.7 Hz, 1H), 2.85-2.50 (m, 4H); ^{13}C NMR (100 MHz, CD_3OD) δ 178.8, 135.5, 126.9, 124.9, 124.5, 121.9, 117.8, 112.8, 112.5, 77.3, 29.0, 28.1; Anal. calcd for $C_{12}H_{10}ClNO_2$: C, 61.16; H, 4.28; N, 5.94. Found: C, 61.29; H, 4.34; N, 5.82.

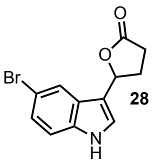

28

<u>5-(5-Bromo-1H-indol-3-yl)oxolan-2-one (28)</u> (0.013 g, 79%) was prepared according to the representative procedure from **39** (0.016 g, 0.057 mmol). Purification by silica gel chromatography (60% EA/ 40% hexanes) afforded **40** as a white solid. mp = 147–150 ºC; TLC R_f = 0.34 (hexanes : EA 30%:70%); IR (film, cm^{-1}) ν_{max} 3216, 2908, 1742, 1546; ^{1}H NMR (400 MHz, DMSO-*d6*) δ 11.4 (s, 1H), 7.77 (d, *J* = 1.7 Hz, 1H), 7.58 (d, *J* =

2.4 Hz, 1H), 7.39 (d, J = 8.8 Hz, 1H), 7.25(dd, J = 8.6, 1.8 Hz, 1H), 5.83(dd, J = 8.6, 6.9 Hz, 1H) 2.77-2.45 (m, 4H); ^{13}C NMR (100 MHz, DMSO-d_6) 177.5, 135.8, 128.0, 126.2, 124.6, 121.5, 114.3, 113.0, 112.3, 76.3, 29.7, 28.4; Anal. calcd for $C_{12}H_{10}BrNO_2$: C, 51.45; H, 3.60; N, 5.00. Found: C, 51.39; H, 3.39; N, 4.93.

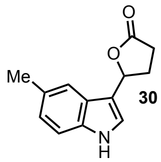

30

5-(5-Methyl-1H-indol-3-yl)oxolan-2-one (30) (0.046 g, 47%) was prepared according to the representative procedure from **43** (0.1 g, 0.46 mmol). Purification by silica gel chromatography (60% EA/ 40% hexanes) afforded **44** as a brown solid. mp = 64-67 ºC; TLC R_f = 0.50 (hexanes : EA 40%:60%); IR (film, cm^{-1}) ν_{max} 3296, 2916, 1737, 1551; ^{1}H NMR (400 MHz, acetone-d_6) δ 10.2 (bs, 1H), 7.47 (s, 1H), 7.43 (s, 1H), 7.34 (d, J = 8.3 Hz, 1H), 7.01 (d, J = 7.7 Hz, 1H), 5.86 (t, J = 6.2 Hz, 1H) 2.78-2.54 (m, 4H), 2.42 (s, 3H); ^{13}C NMR (100 MHz, acetone-d_6) 176.5, 135.5, 128.2, 126.4, 123.5, 123.3, 123.2, 118.6, 113.3, 111.4, 111.3, 76.5, 20.7; Anal. calcd for $C_{13}H_{13}NO_2$: C, 72.54; H, 6.09; N, 6.51. Found: C, 72.46; H, 6.23; N, 6.67.

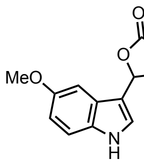

32

5-(5-Methoxy-1H-indol-3-yl)oxolan-2-one (32) (0.084 g, 85%) was prepared according to the representative procedure from **47** (0.1 g, 0.43 mmol). Purification by silica gel chromatography (100% EA) afforded **48** as a brown solid. mp = 59–61 ºC; TLC R_f = 0.30 (50% EA/50%H); IR (film, cm^{-1}) ν_{max} 3371, 3084, 2975, 1744, 1625, 1485, 1211, 1172; ^{1}H NMR (400 MHz, DMSO-d_6) δ 11.1 (bs, 1H), 7.47(d, J = 2.6 Hz, 1H), 7.30 (d, J = 8.8 Hz, 1H), 7.05 (d, J = 2.2 Hz, 1H), 6.79 (dd, J = 8.8, 2.3 Hz, 1H), 5.82(t, J = 7.7 Hz, 1H) 2.78-2.52 (m, 4H); ^{13}C NMR (100 MHz, DMSO-d_6) 177.7, 153.9, 132.1, 126.8, 125.2, 113.0, 112.8, 112.2, 100.9, 76.7, 55.8, 29.9, 28.2; Anal. calcd for $C_{13}H_{13}NO_3$: C, 67.52; H, 5.67; N, 6.06. Found: C, 67.36; H, 5.64; N, 5.87.

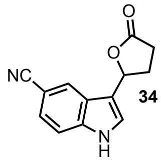

34

5-(5-Cyano-1H-indol-3-yl)oxolan-2-one (34) (0.019 g, 42%) was prepared according to the representative procedure from **51** (0.045 g, 0.2 mmol). Purification by silica gel chromatography (100% EA) afforded **52** as a yellow solid. mp = 179–182 ºC; TLC R_f = 0.65 (100% EA); IR (film, cm^{-1}) ν_{max} 3242, 3086, 2924, 2272, 1743, 1619, 1551; ^{1}H NMR (400 MHz, CD_3OD) δ 8.09 (d, J = 0.6 Hz, 1H), 7.57-7.55 (m, 2H), 7.47(dd, J = 8.5, 1.3 Hz, 1H), 5.94(dd, J = 8.5, 6.6 Hz, 1H) 2.87-2.52 (m, 4H); ^{13}C NMR (100 MHz, DMSO-d_6) 177.5, 138.8, 127.4, 125.9, 125.1, 124.9, 121.0, 114.4, 113.6, 101.8, 76.0, 29.7, 28.6; Anal. calcd for $C_{13}H_{10}N_2O_2$: C, 69.02; H, 4.46; N, 12.38. Found: C, 69.34; H, 4.40; N, 12.14.

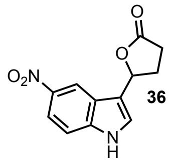

36

5-(5-Nitro-1H-indol-3-yl)oxolan-2-one (36) (0.033 g, 33%) was prepared according to the representative procedure from **55** (0.10 g, 0.4 mmol). Purification by silica gel chromatography (100% EA) afforded **55** as a yellow solid. mp = 165–168 ºC; TLC R_f = 0.25 (60% EA/40% H); IR (film, cm^{-1}) ν_{max} 3302, 3119, 2916, 1734, 1623, 1514; ^{1}H NMR (400 MHz, acetone-d_6) δ 11.05 (bs, 1H), 8.67 (d, J = 2.1 Hz, 1H), 8.11(dd, J = 9.0, 2.2 Hz, 1H), 7.77 (s, 1H), 7.66 (d, J = 9.0 Hz, 1H) 6.03-6.00 (m, 1H), 2.81-2.55 (m, 4H); ^{13}C NMR (100 MHz,

DMSO-d_6) δ 177.4, 141.3, 140.2, 128.3, 125.5, 117.5, 116.4, 116.1, 112.9, 75.7, 29.6, 28.5; Anal. calcd for $C_{12}H_{10}N_2O_4$: C, 58.54; H, 4.09; N, 11.38. Found: C, 58.76; H, 4.16; N, 11.43.

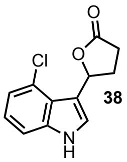

5-(4-Chloro-1H-indol-3-yl)oxolan-2-one (38) (0.026 g, 40%) was prepared according to the representative procedure from **63** (0.067 g, 0.28 mmol). Purification by silica gel chromatography (70% EA/ 30% hexanes) afforded **64** as a yellow solid. mp = 135–137 °C; TLC R_f = 0.39 (hexanes : EA 50%:50%); IR (film, cm^{-1}) ν_{max} 3308, 3183, 3039, 2903, 1740, 1426; ^{1}H NMR (400 MHz, DMSO-d_6) δ 11.6 (bs, 1H), 7.64 (d, *J* = 2.6 Hz, 1H), 7.40 (dd, *J* = 7.4, 1.5 Hz, 1H), 7.14-7.09 (m, 2H), 6.19 (t, *J* = 7.0 Hz, 1H), 2.79-2.45 (m, 4H); ^{13}C NMR (100 MHz, DMSO-d_6) δ 177.5, 138.7, 126.3, 124.6, 123.2, 122.9, 120.6, 113.4, 111.6, 76.0, 29.6, 29.3; Anal. calcd for $C_{12}H_{10}ClNO_2$: C, 61.16; H, 4.28; N, 5.94. Found: C, 61.16; H, 4.37; N, 5.88.

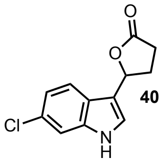

5-(6-Chloro-1H-indol-3-yl)oxolan-2-one (40) (0.046 g, 46%) was prepared according to the representative procedure from **67** (0.1 g, 0.42 mmol). Purification by silica gel chromatography (70% EA/ 30% hexanes) afforded **68** as a purple solid. mp = 121–124 °C; TLC R_f = 0.51 (hexanes : EA 30%:70%); IR (film, cm^{-1}) ν_{max} 3397, 3197, 3042, 2912, 1742, 1456; ^{1}H NMR (400 MHz, $CDCl_3$) δ 8.23 (bs, 1H), 7.57 (d, *J* = 8.5 Hz, 1H), 7.42 (d, *J* = 1.6 Hz, 1H), 7.24 (d, *J* = 2.0 Hz, 1H), 7.16 (dd, *J* = 8.5, 1.8 Hz, 1H), 5.87-5.83 (m, 1H), 2.76-2.66 (m, 3H). 2.56-2.45 (m, 1H); ^{13}C NMR (100 MHz, CD_3OD) δ 178.8, 137.5, 127.6, 124.5, 123.9, 119.6, 119.5, 113.3, 111.1, 77.4, 29.0, 28.2; Anal. calcd for $C_{12}H_{10}ClNO_2$: C, 61.16; H, 4.28; N, 5.94. Found: C, 61.27; H, 4.23; N, 5.96.

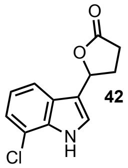

5-(7-Chloro-1H-indol-3-yl)oxolan-2-one (42) (0.042 g, 42%) was prepared according to the representative procedure from **71** (0.1 g, 0.42 mmol). Purification by silica gel chromatography (100% EA) afforded **72** as a red solid. mp = 120–123 °C; TLC R_f = 0.43 (hexanes : EA 50%:50%); IR (film, cm^{-1}) ν_{max} 3324, 2922, 1744, 1438; ^{1}H NMR (400 MHz, DMSO-d_6) δ 11.62 (bs, 1H), 7.60-7.57 (m, 2H), 7.24 (d, *J* = 7.4 Hz, 1H), 7.07 (t, *J* = 7.8 Hz, 1H), 5.84 (dd, *J* = 8.2, 1.0 Hz, 1H), 2.79-2.62 (m, 2H), 2.60-2.46 (m, 2H); ^{13}C NMR (100 MHz, DMSO-d_6) δ 177.5, 133.9, 128.1, 125.9, 121.7, 120.7, 118.4, 116.6, 114.6, 76.3, 29.7, 28.4; Anal. calcd for $C_{12}H_{10}ClNO_2$: C, 61.16; H, 4.28; N, 5.94. Found: C, 61.36; H, 4.55; N, 5.86.

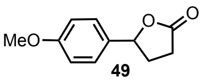

5-(4-Methoxyphenyl)tetrahydrofuran-2-one (49) (0.051 g, 51%) was prepared according to the representative procedure from **48** (0.1 g, 0.52 mmol). Purification by silica gel chromatography (30% EA/ 70% hexanes) afforded **49** as a brown solid. TLC R_f = 0.23 (hexanes : EA 70%:30%); IR (film, cm^{-1}) ν_{max} 3015, 2969, 2843, 1757, 1610 1516; ^{1}H NMR (400 MHz, CD_3OD) δ 7.32 (d, *J* = 8.6 Hz, 2H), 6.96 (d, *J* = 8.7 Hz, 2H), 5.51 (dd, *J* = 8.8, 6.5 Hz, 1H), 3.82 (s, 3H), 2.79-2.57 (m, 3H), 2.27-2.17 (m, 1H); ^{13}C NMR (100 MHz, $CDCl_3$) δ 176.9, 159.8, 131.2, 127.0, 114.1, 81.3, 55.3, 30.9, 29.2. This compound has been previously prepared.[10]

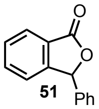

51

3-Phenylphthalide (51) (0.021 g, 21%) was prepared according to the representative procedure from **50** (0.1 g, 0.47 mmol). Purification by silica gel chromatography (25% EA/ 75% hexanes) afforded **51** as a white solid. TLC R_f = 0.41 (hexanes : EA 75%:25%); IR (film, cm^{-1}) ν_{max} 3063, 3032, 2922, 1743, 1611 1285; ^1H NMR (400 MHz, $CDCl_3$) δ 8.00 (d, *J* = 7.4 Hz, 1H), 7.68 (t, *J* = 7.4 Hz, 1H), 7.59 (t, *J* = 7.4 Hz, 1H), 7.42-7.29 (m, 6H), 6.44 (s, 1H); ^{13}C NMR (100 MHz, $CDCl_3$) δ 170.5, 149.7, 136.4, 134.3, 129.4, 129.3, 129.0, 127.0, 125.7, 125.6, 122.9, 82.7. This compound has been previously prepared.[11]

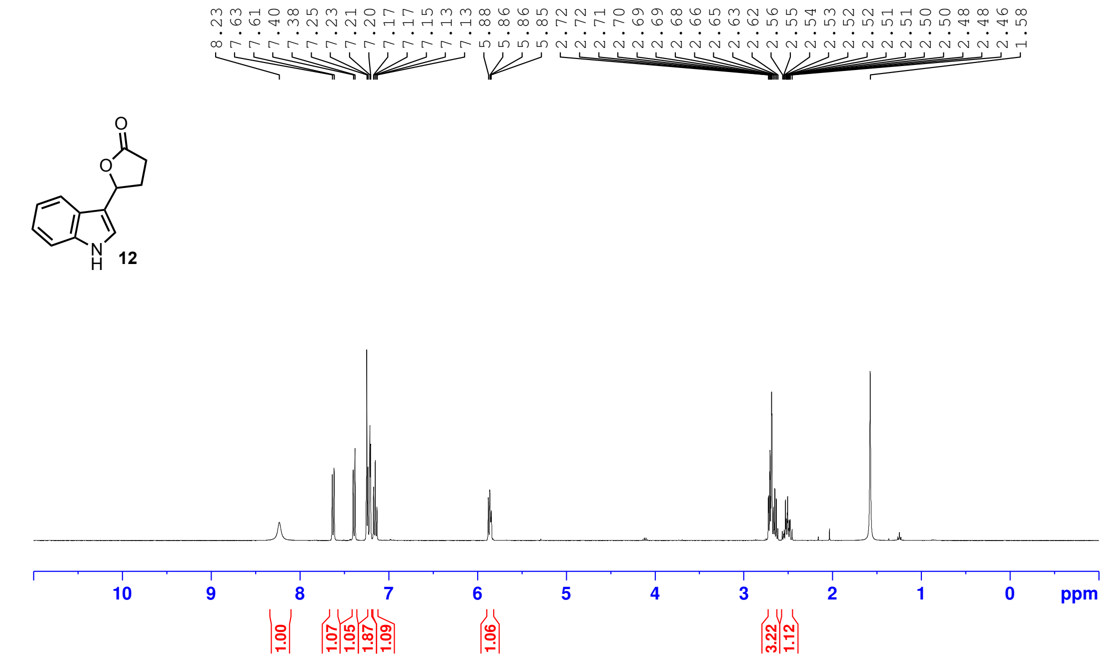

CN_2_115 - 1H NMR - 400.16 MHz - CDCl3

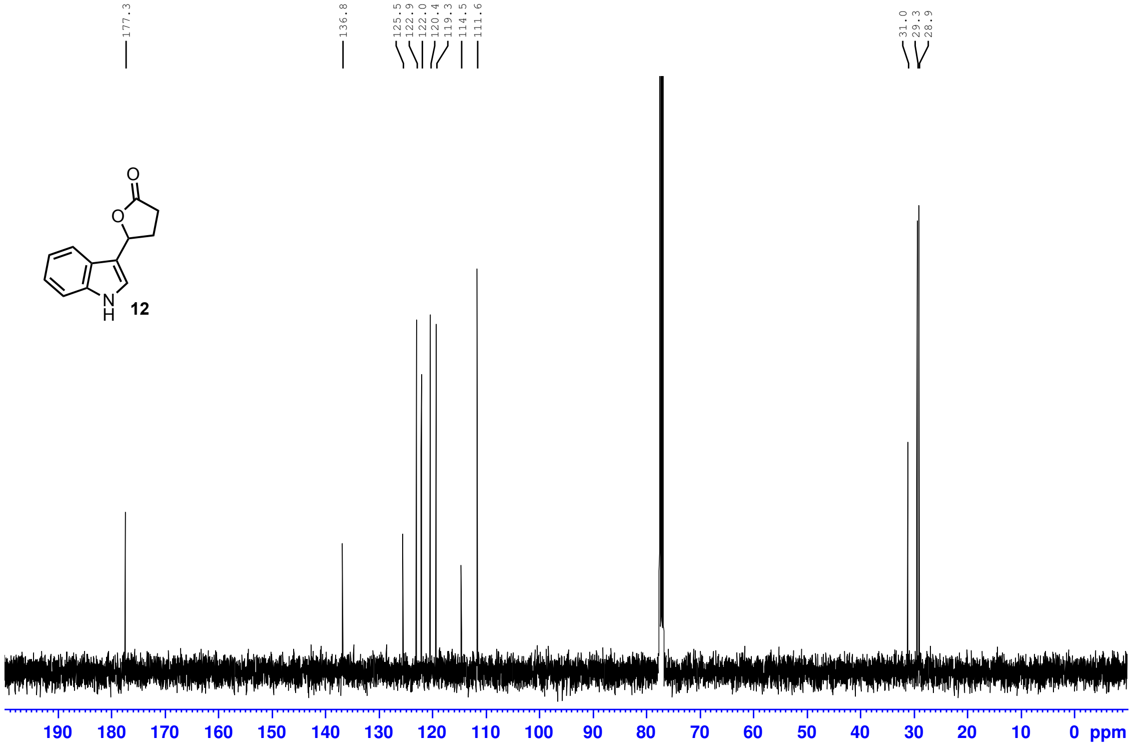

CN_2_115 - 13C NMR - 100.16 MHz - CDCl3

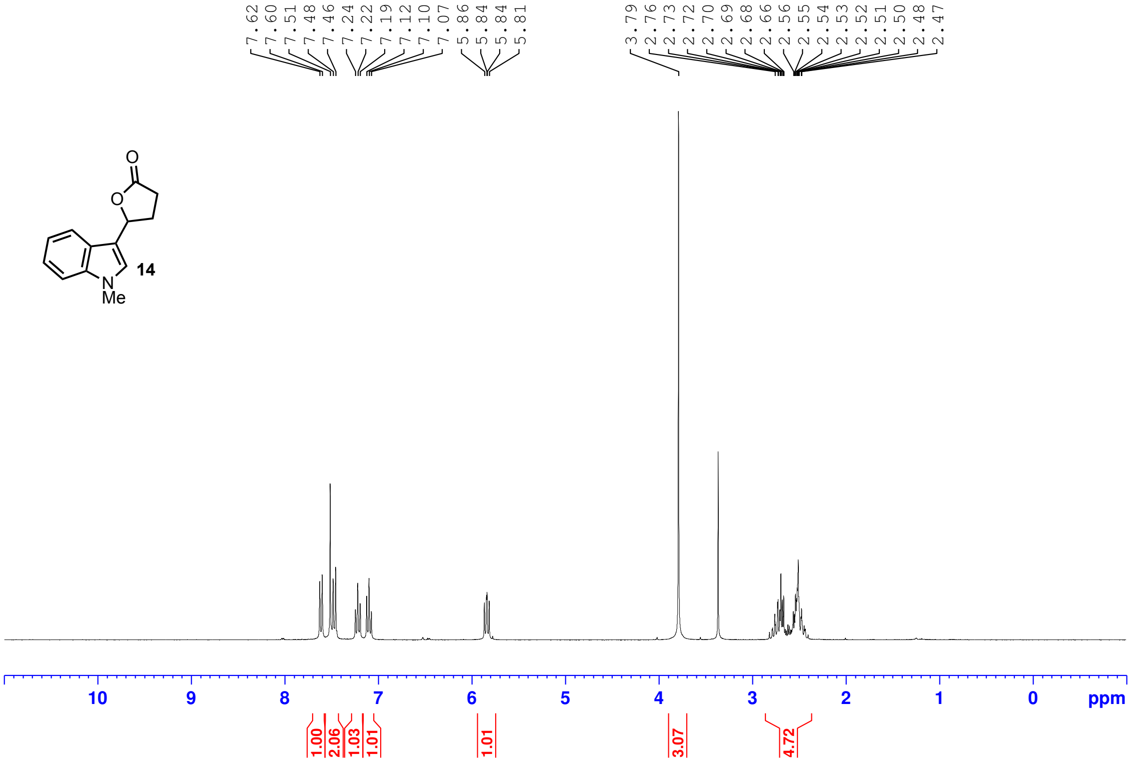

CN_4_129 - 1H NMR - 300 MHz - DMSO-d6

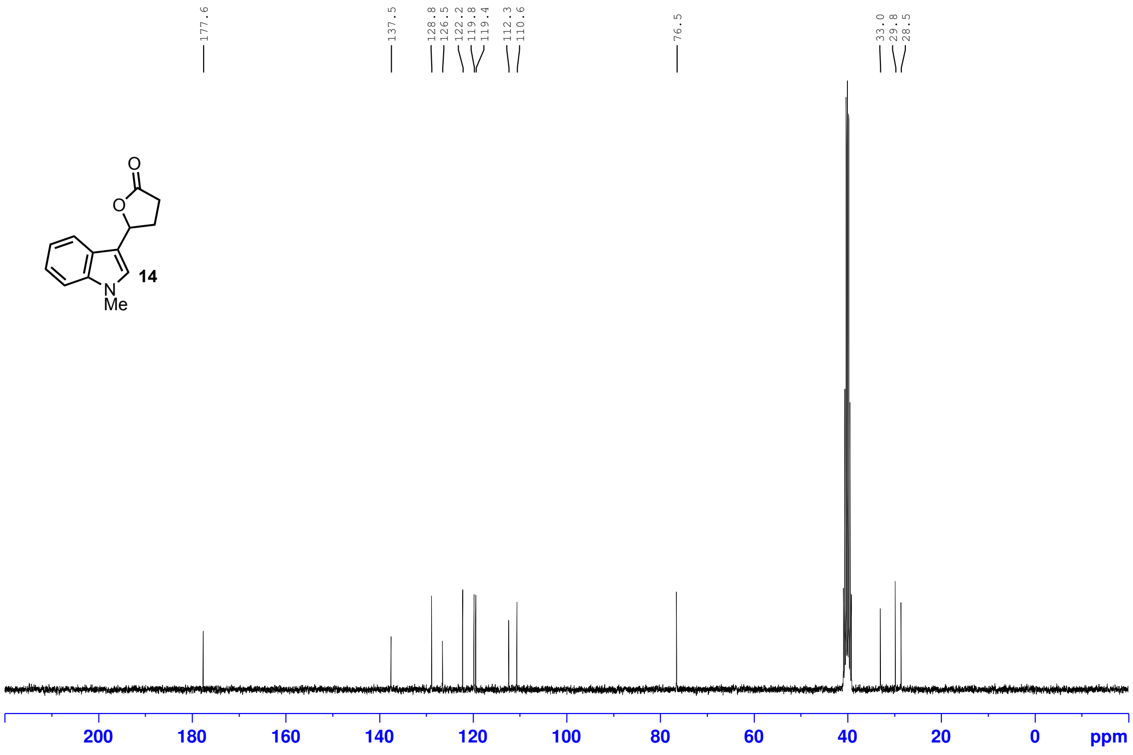

CN_4_129 -13C NMR - 75 MHz - DMSO-d6

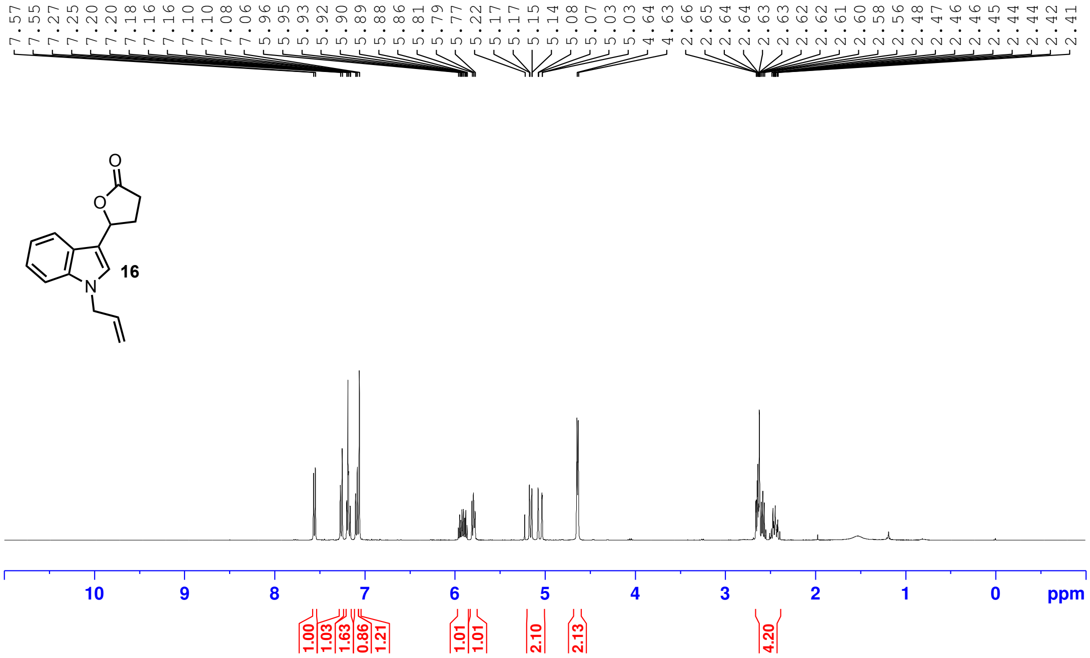

CN_3_47 - 1H NMR - 400.16 MHz - CDCl3

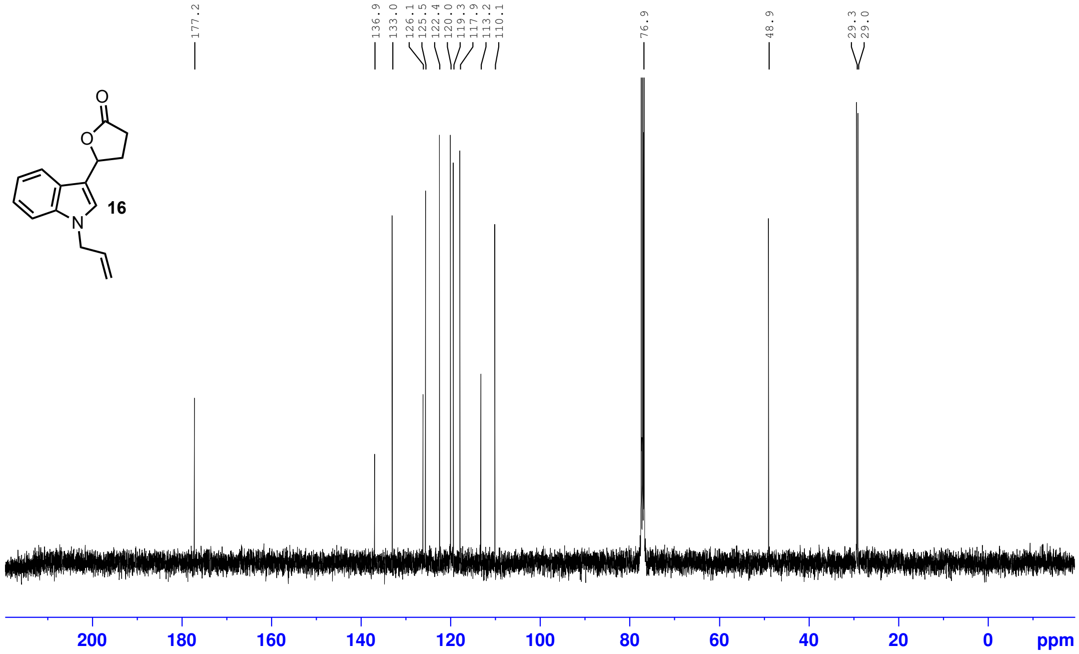

CN_3_47 - 13C NMR - 100.62 MHz - CDCl3

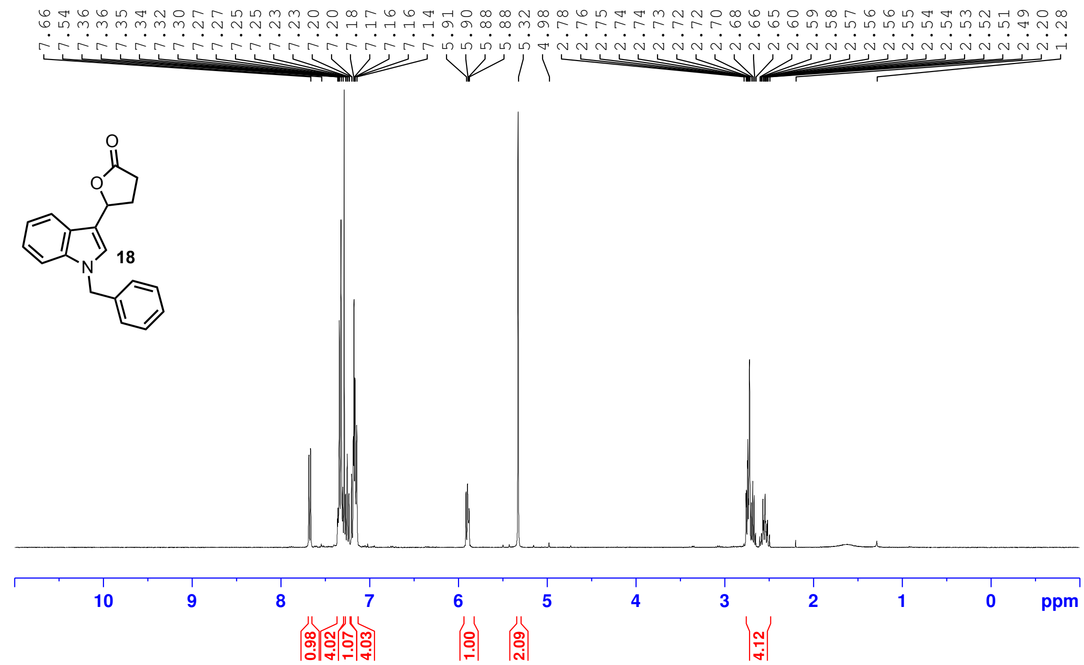

CN_3_57 - 1H NMR - 400.16 MHz - CDCl3

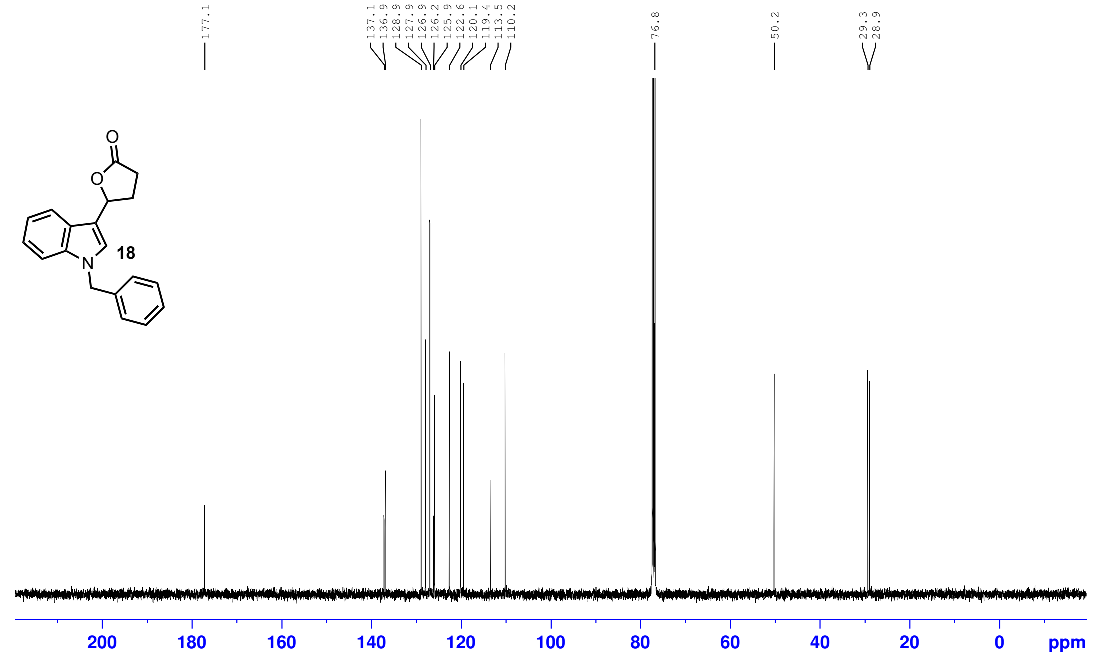

CN_3_57 - 13C NMR - 100.62 MHz - CDCl3

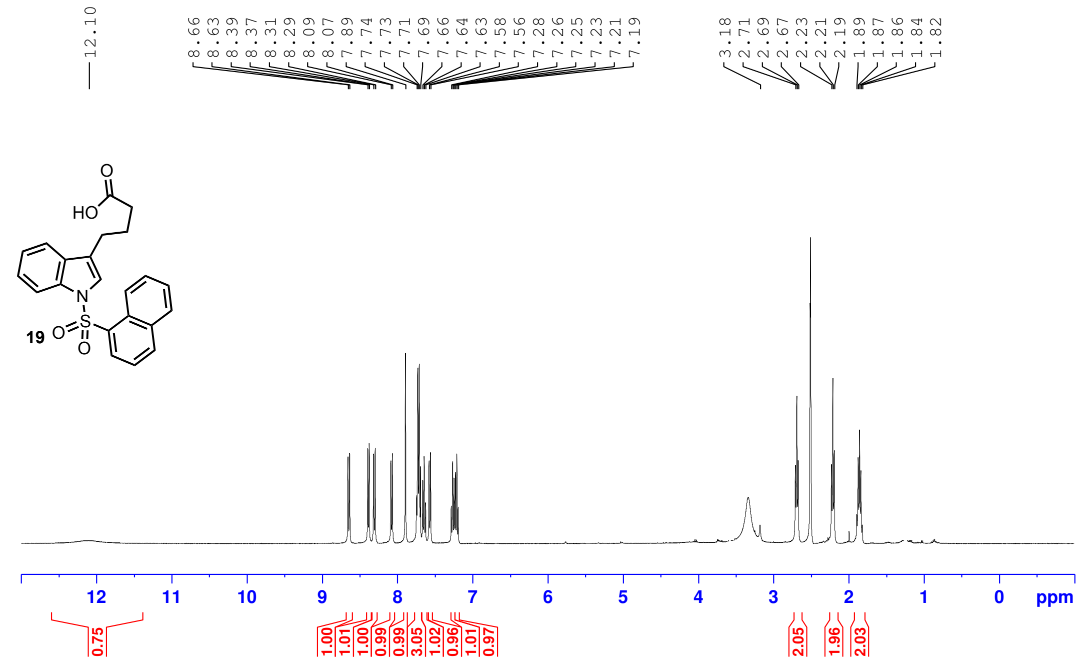

CN_3_100 - 1H NMR - 400.16 MHz - DMSO-d6

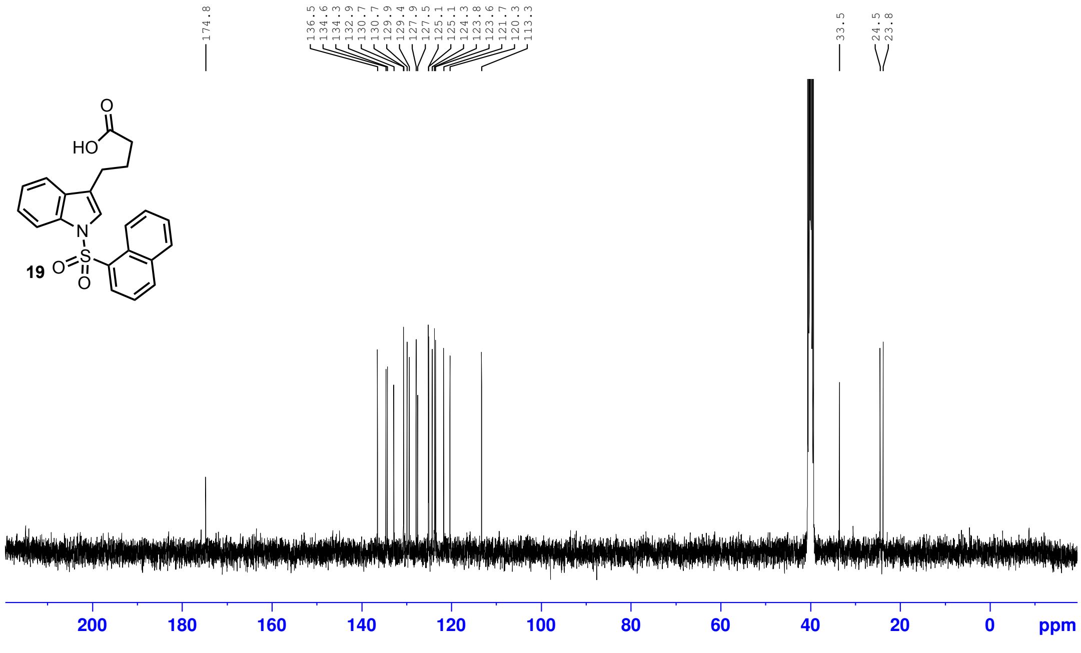

CN_3_100 - 13CNMR - 100.62 MHz - DMSO-d6

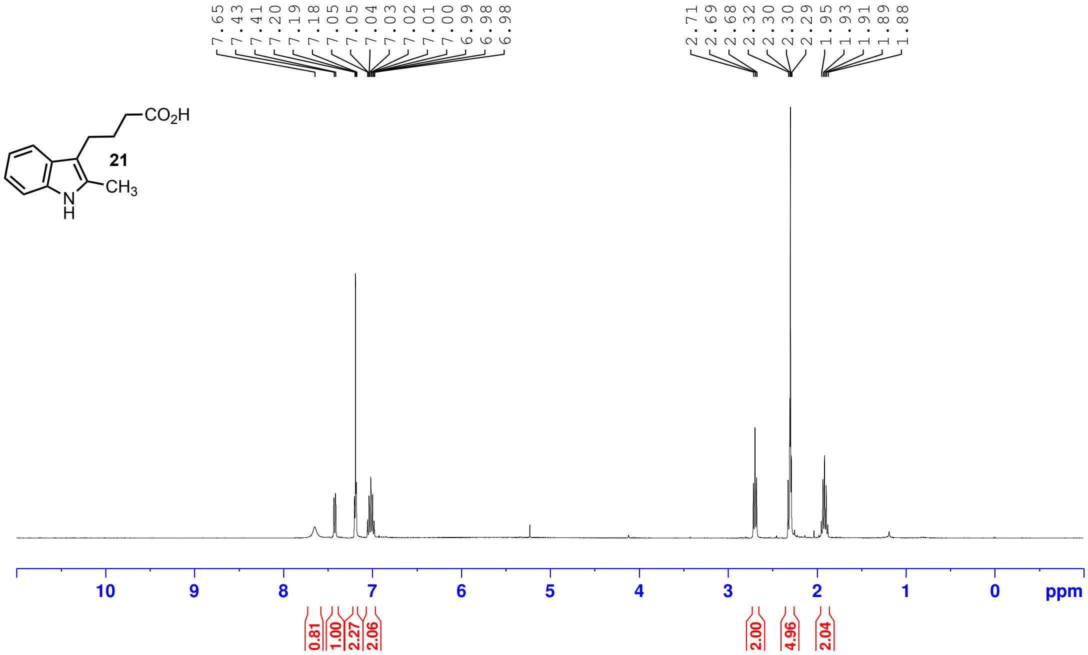

CN_3_8 - 1H NMR - 400.16 MHz - CDCl3

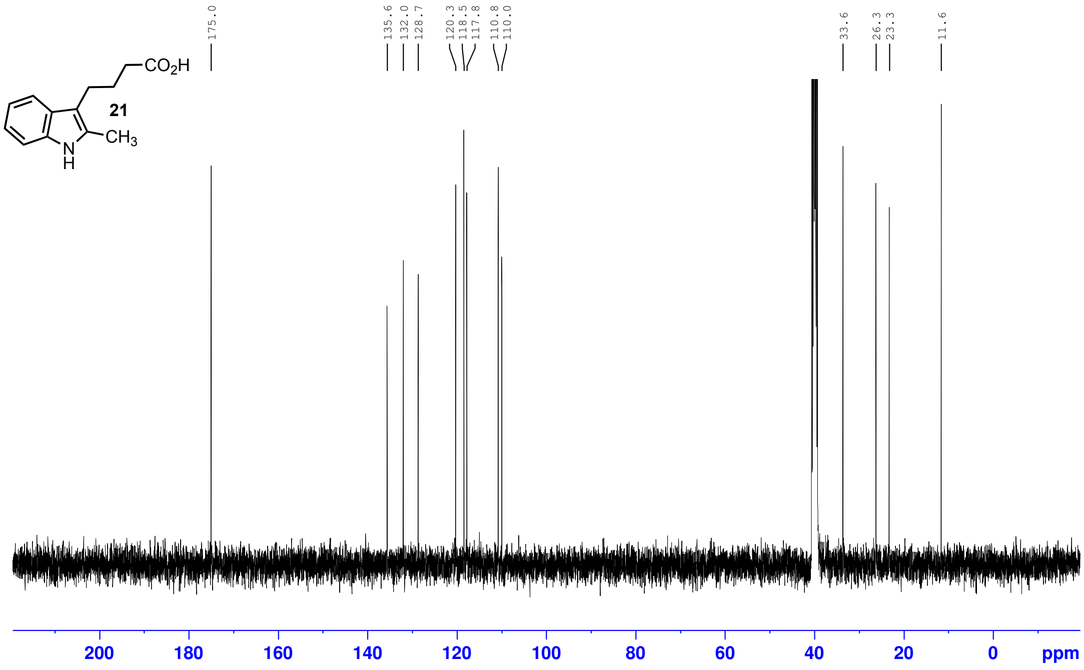

CN_3_8 - 13C NMR - 100.62 MHz - DMSO-d6

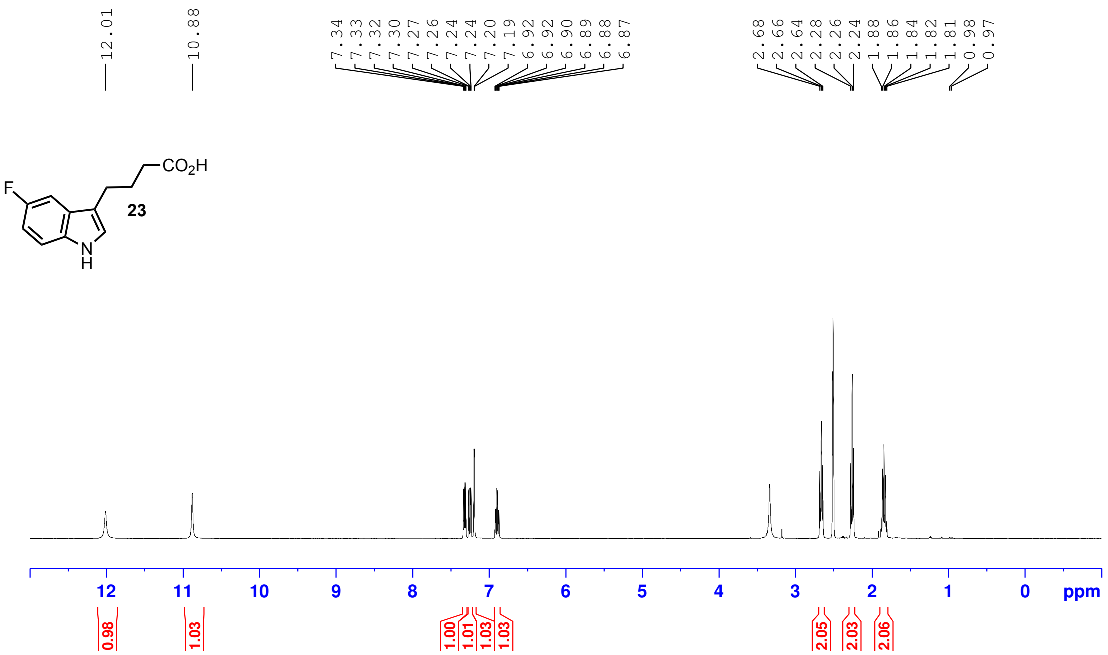

CN_3_55 - 1H NMR - 400.16 MHz - DMSO-d6

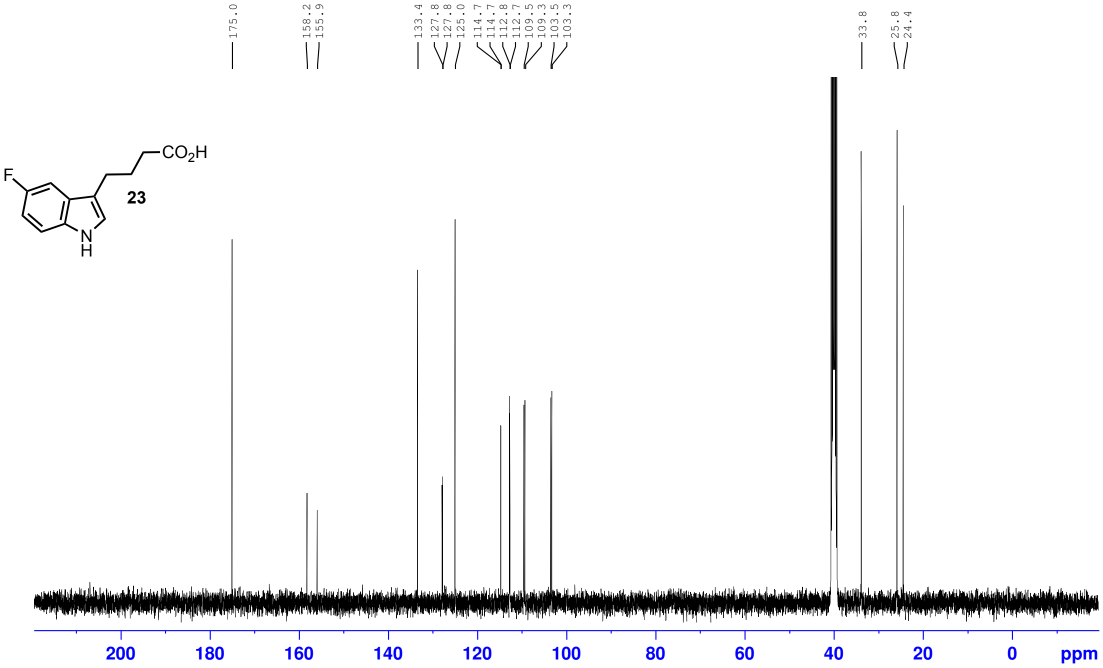

CN_3_55 - 13C NMR - 100.62 MHz - DMSO-d6

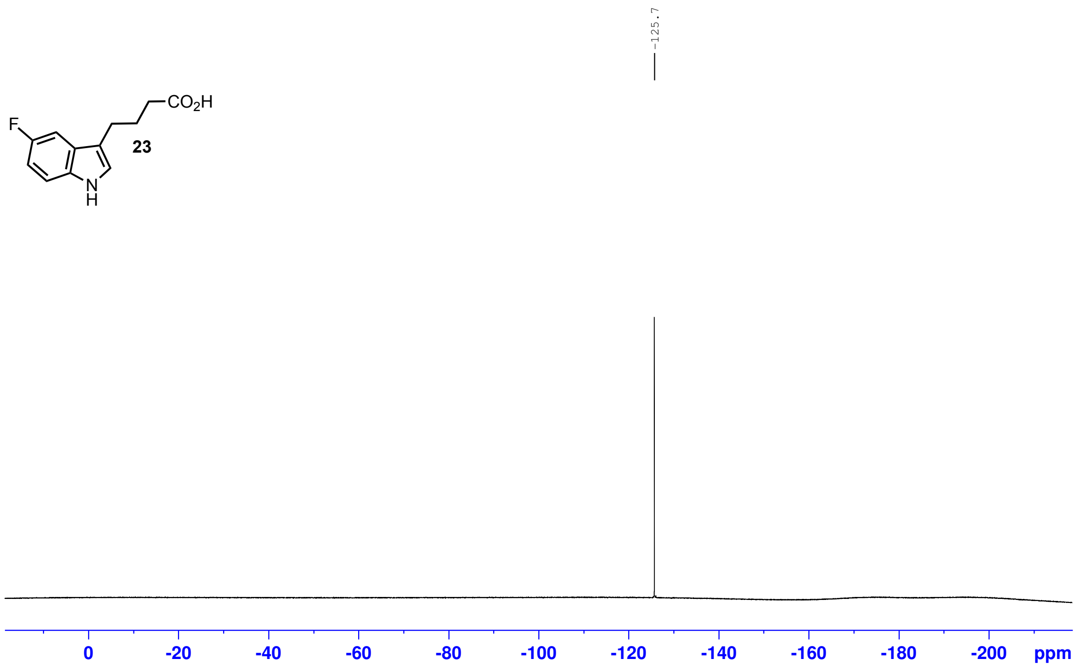

CN_3_55 - 19F NMR - 376.50 MHz - DMSO-d6

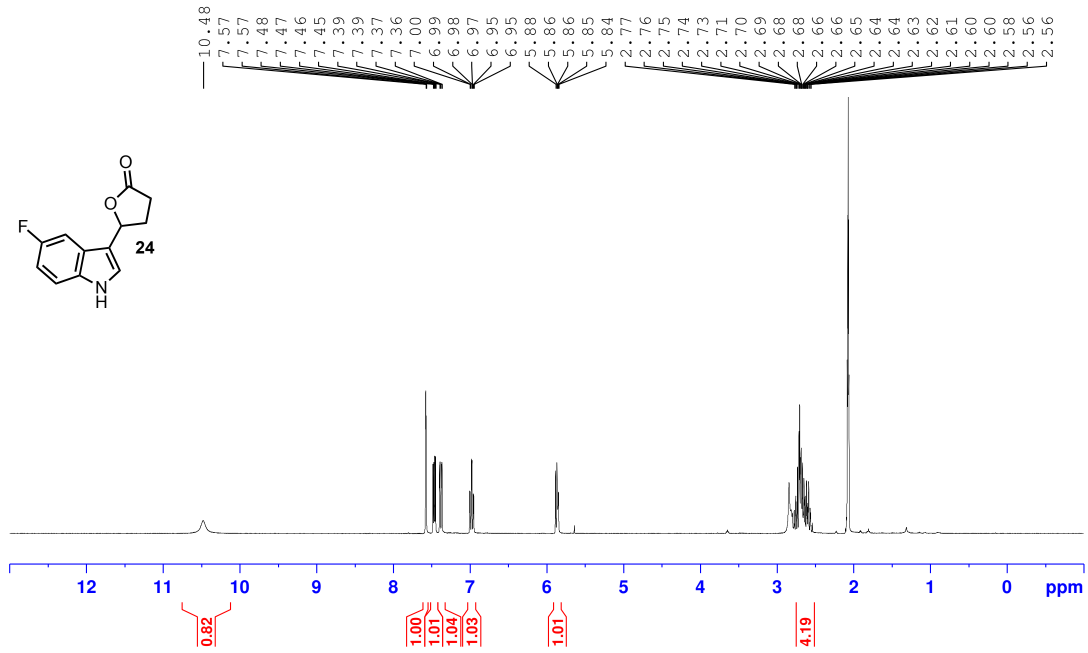

CN_3_58 - 1HNMR - 400.16 MHz - Acetone-d6

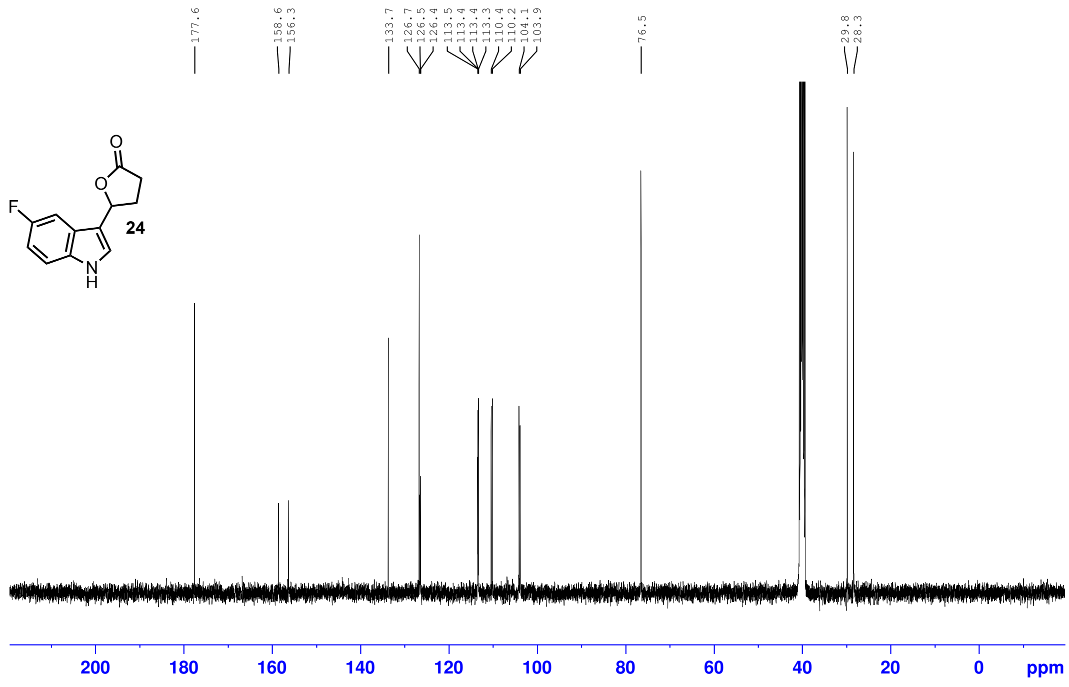

CN_3_58 - 13CNMR - 100.62 MHz - DMSO-d6

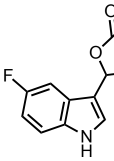

24

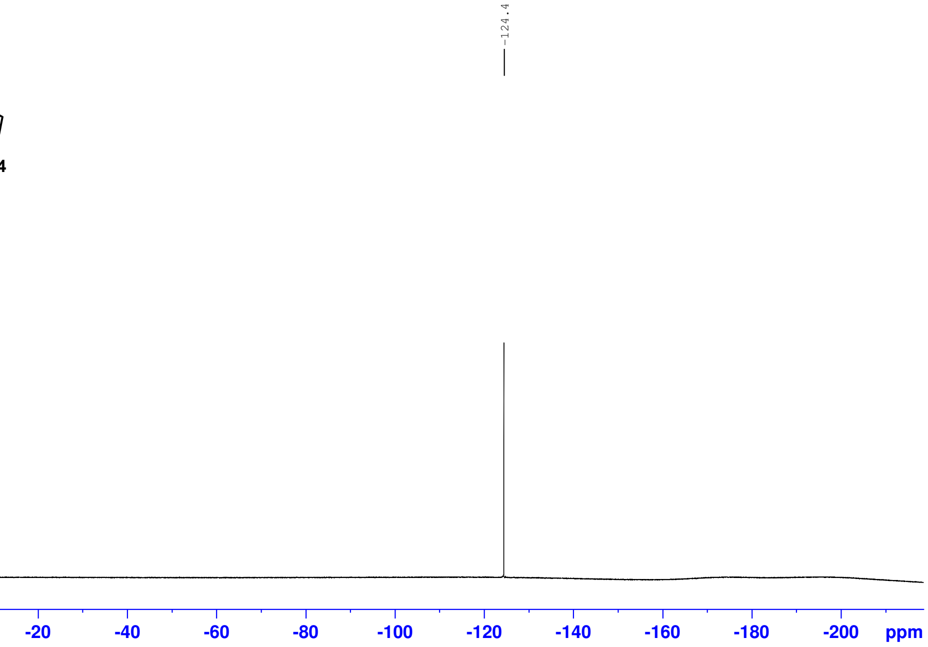

CN_3_58 - 19FNMR - 376.50 MHz - DMSO-d6

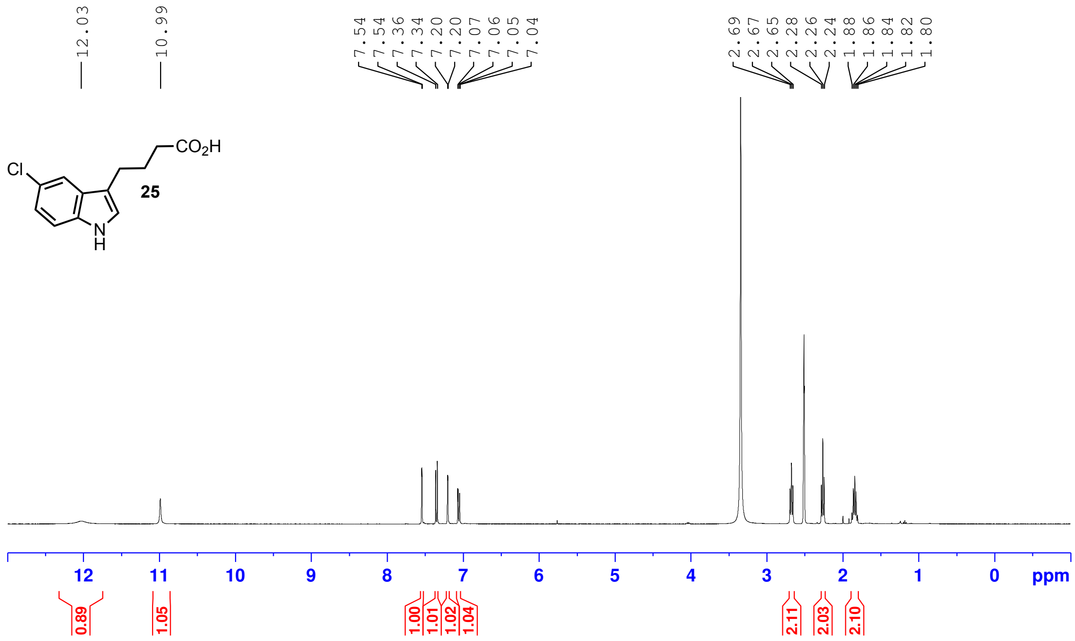

CN_3_18 - 1H NMR - 400.16MHz - DMSO-d6

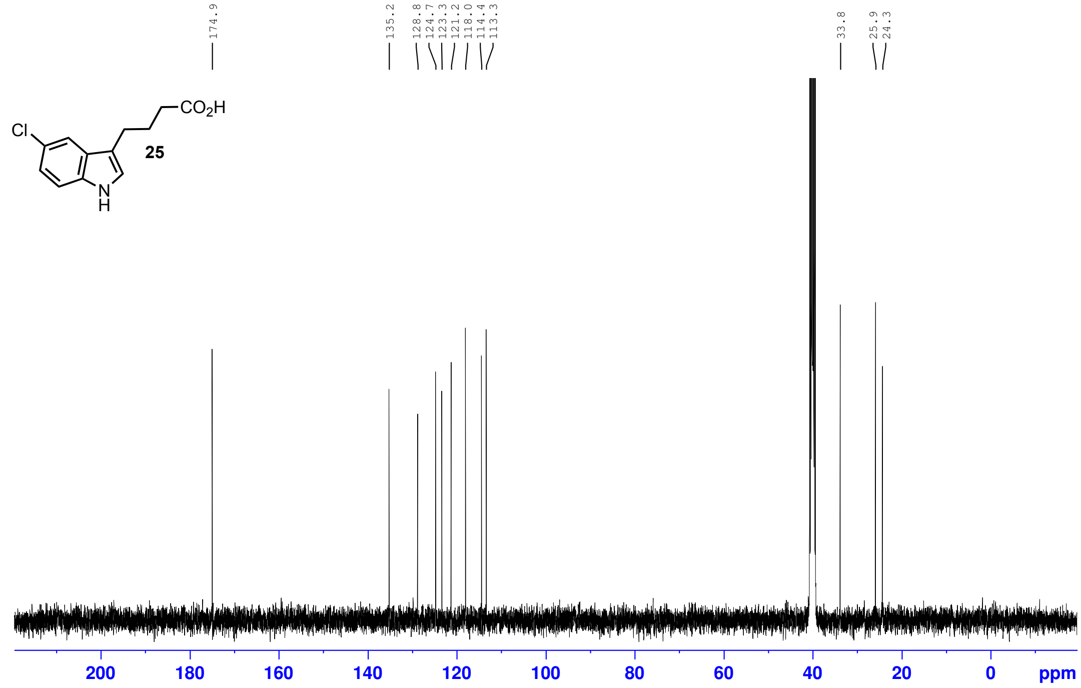

CN_3_18 - 13C NMR - 100.62 MHz - DMSO-d6

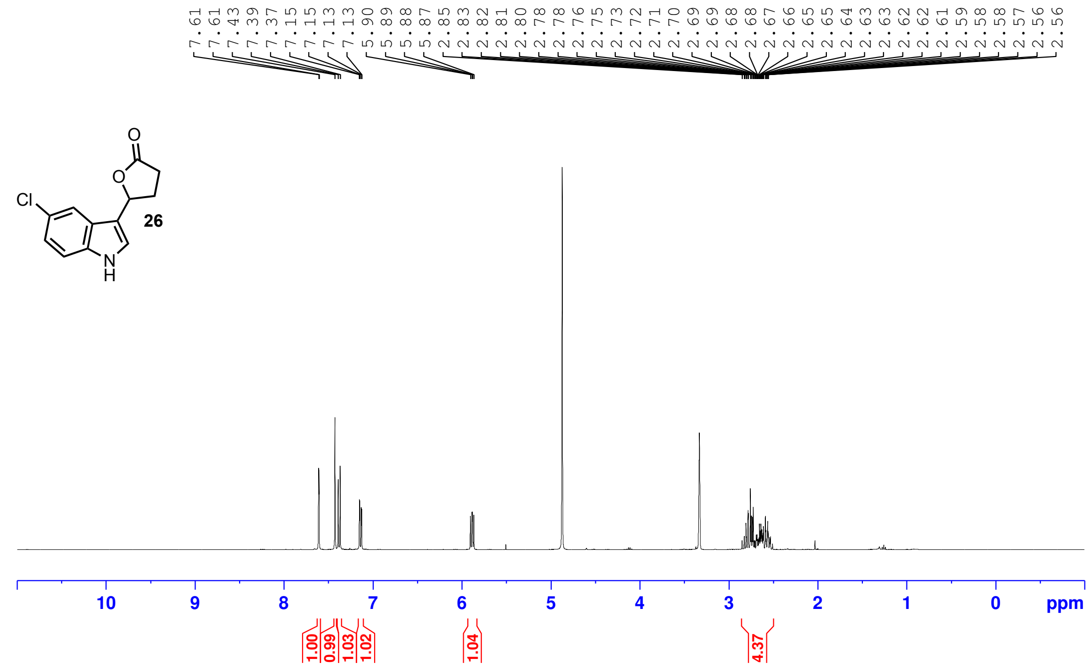

CN_3_19 - 1H NMR - 400.16 MHz - MeOD

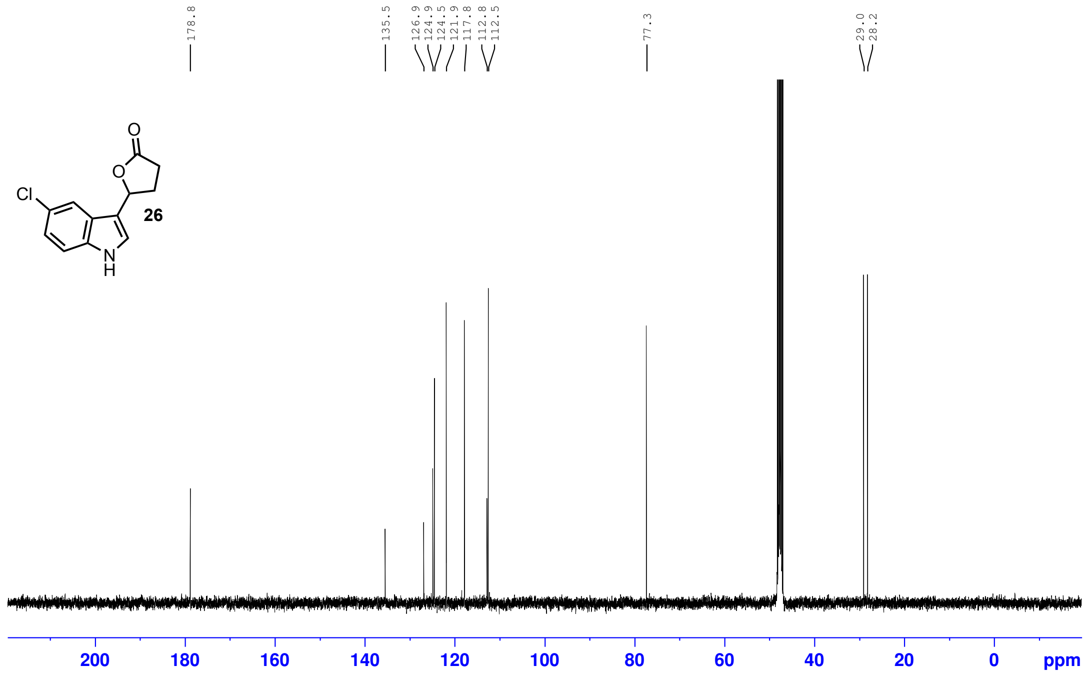

CN_3_19 - 13C NMR - 100.62 MHz - MeOD

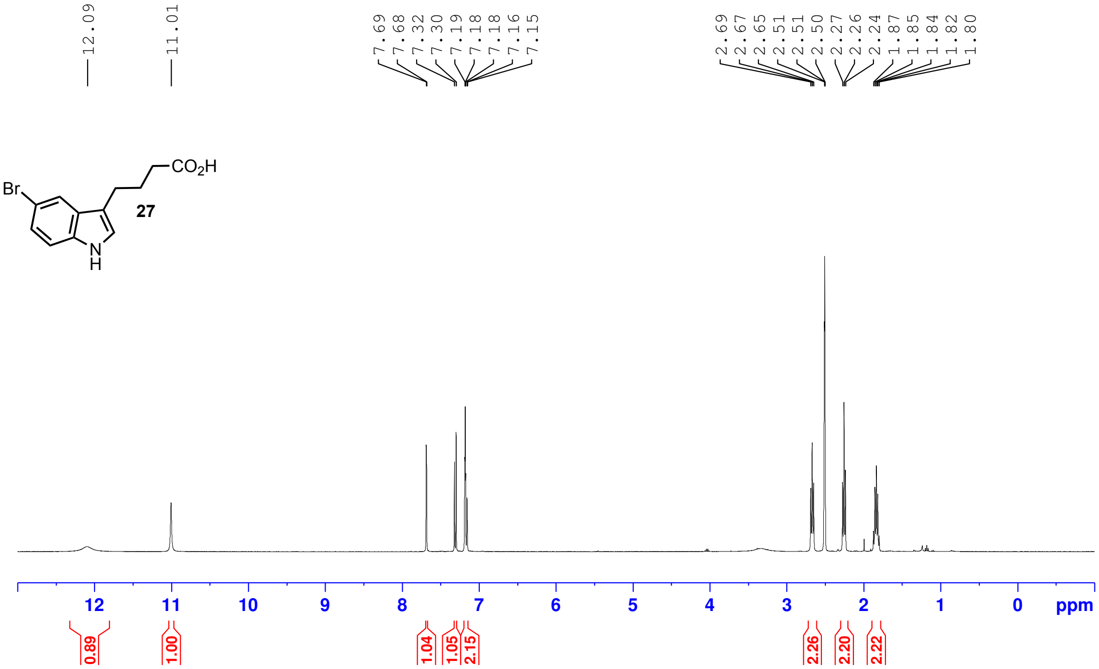

CN_2_89 - 1H NMR - 400.16 MHz - DMSO-d6

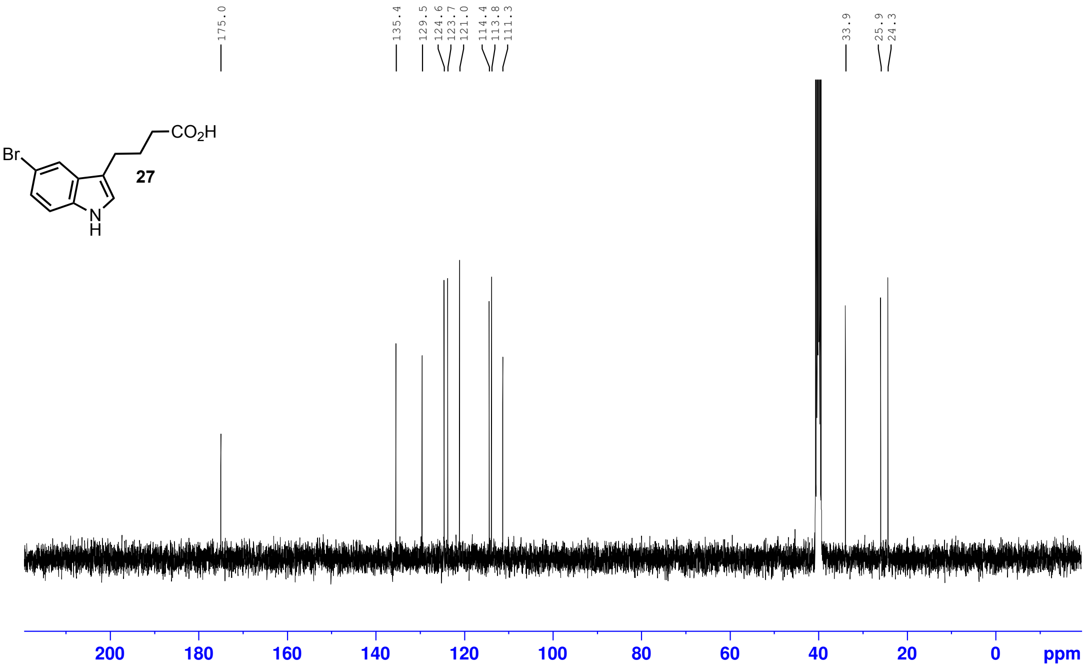

CN_2_89 - 13C NMR - 100.62MHz - DMSO-d6

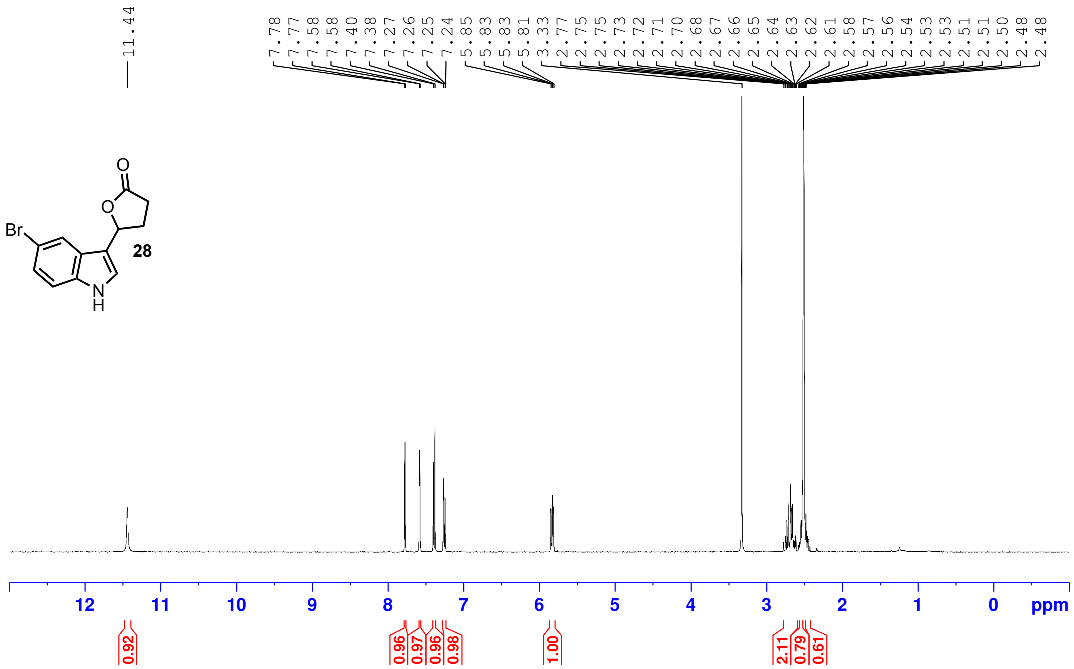

CN_3_20 - 1H NMR - 400.16 MHz - DMSO-d6

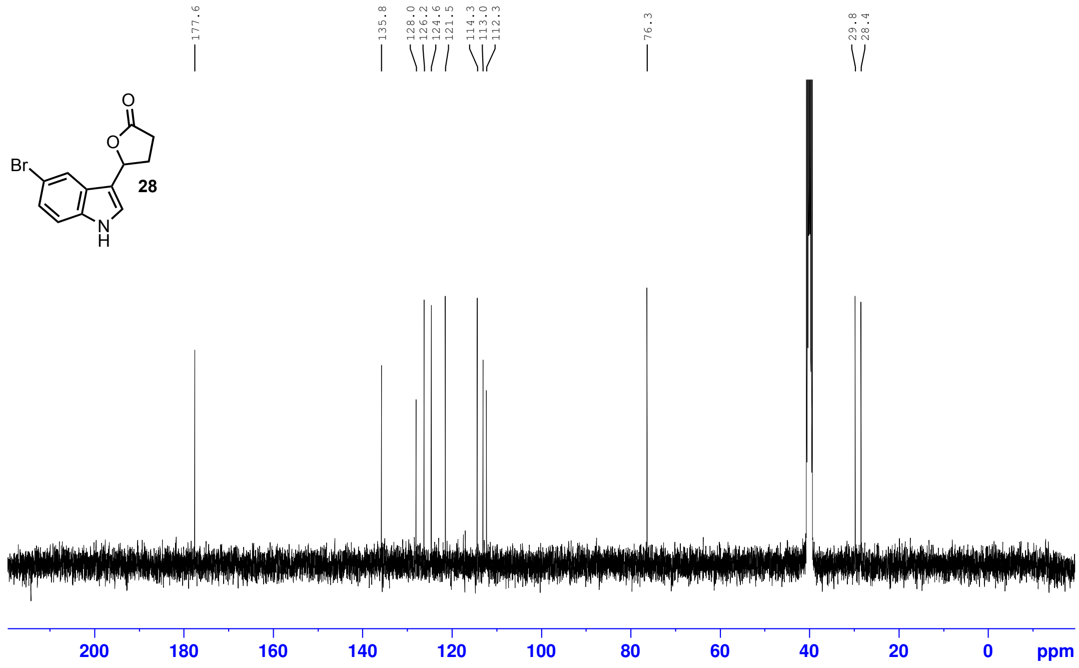

CN_3_20 - 13C NMR - 100.62 MHz - DMSO - d6

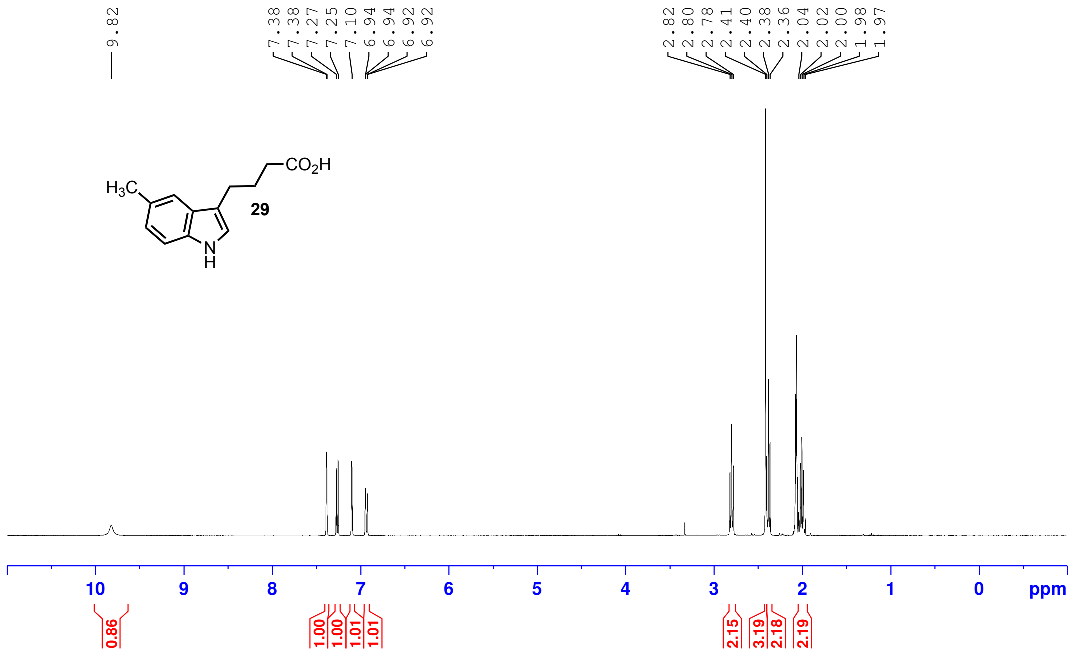

CN_3_67 - 1H NMR - 400.16 MHz - acetone-D6

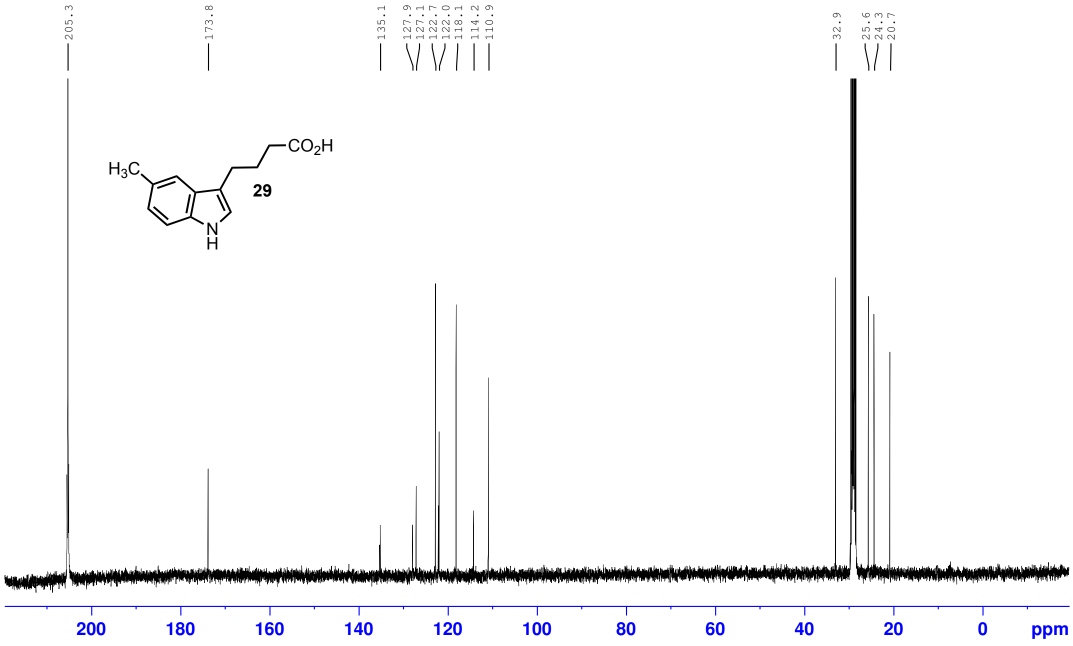

CN_3_67 - 13CNMR - 100.62 MHz - Acetone-d6

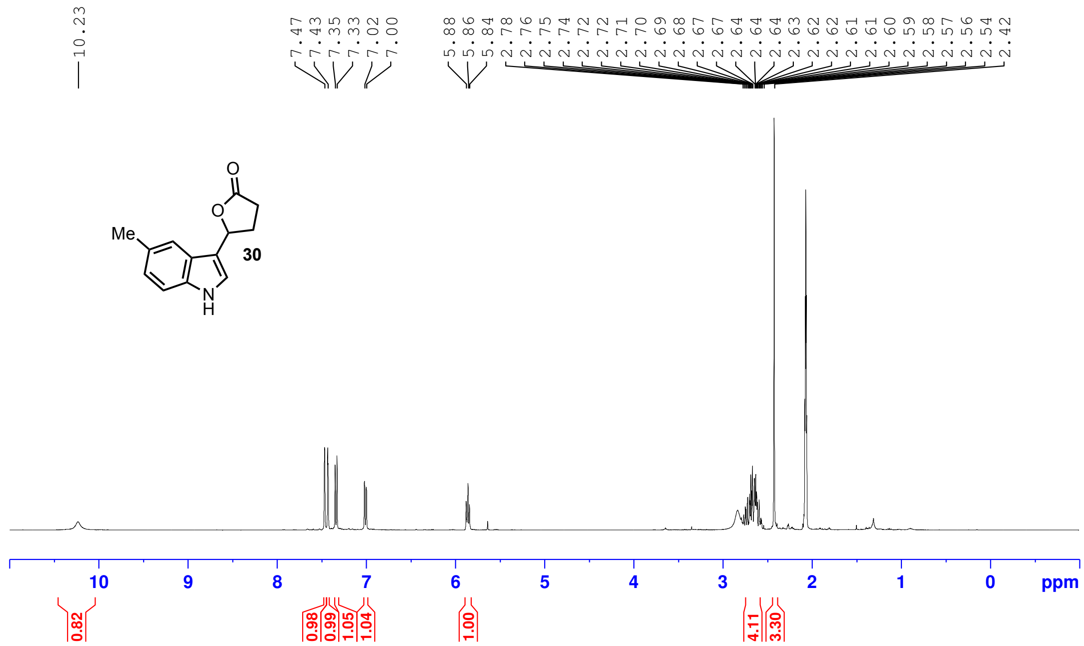

CN_3_68 - 1H NMR - 400.16 MHz - Acetone-d6

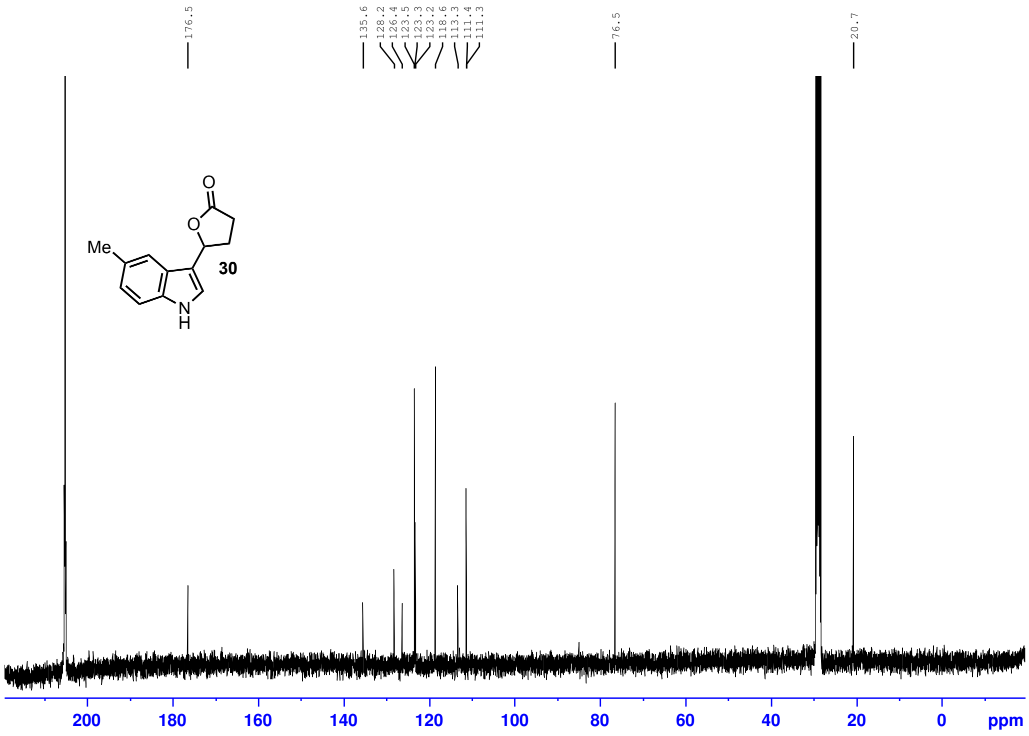

CN_3_68 - 13C NMR - 100.62 MHz - acetone-d6

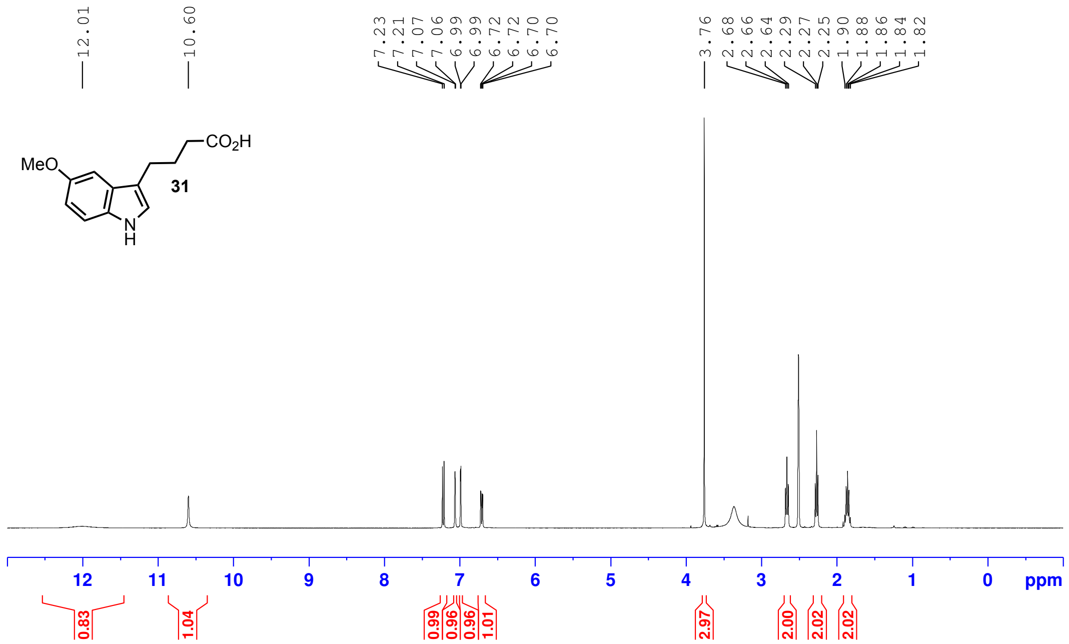

CN_3_54 - 1H NMR - 400.16 MHz - DMSO-d6

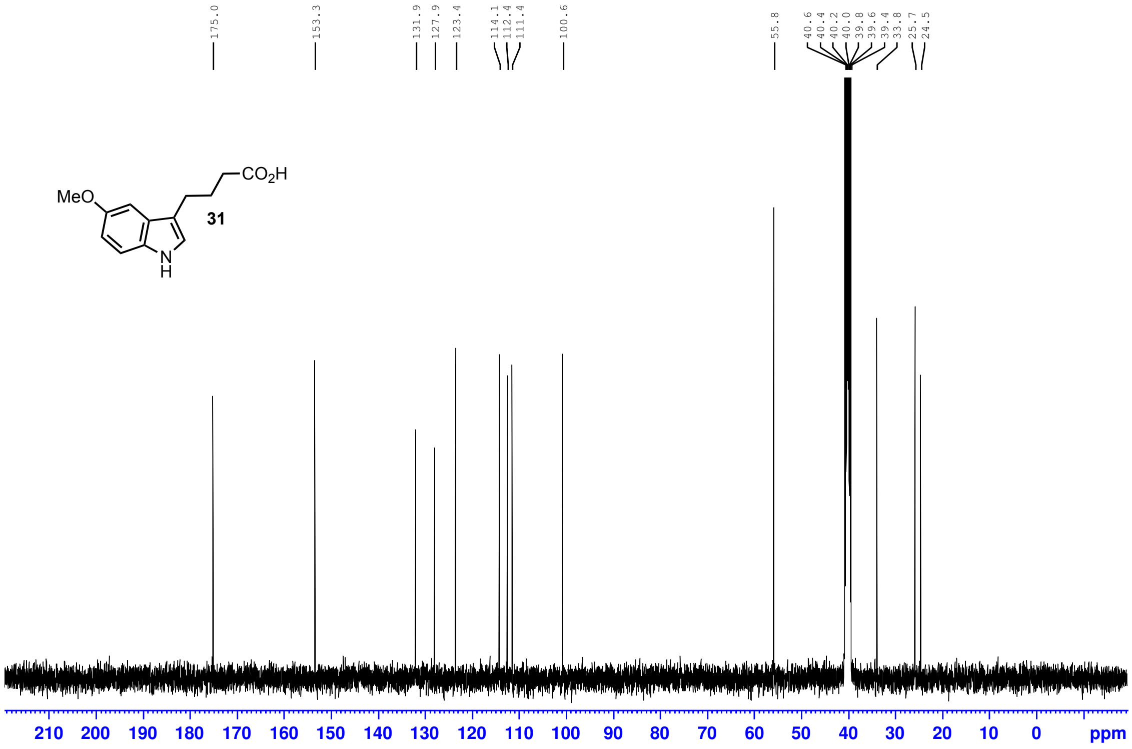

CN_3_54 - 13C NMR - 100.62 MHz - DMSO-d6

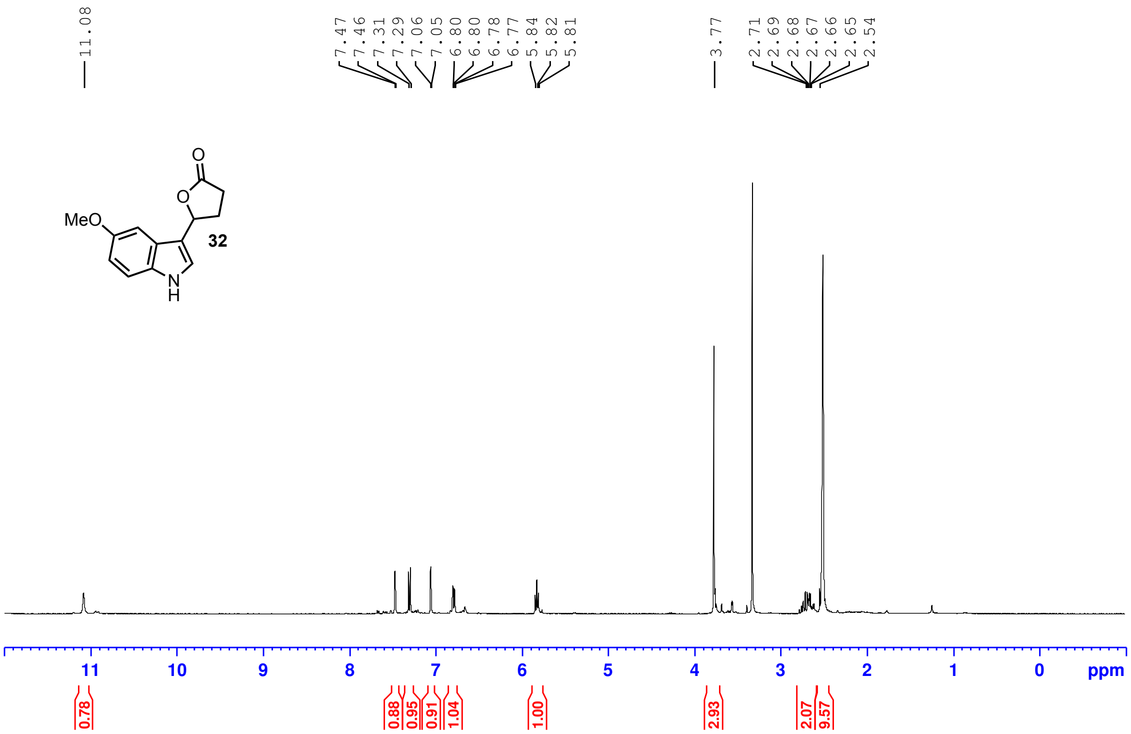

CN_3_56 - 1H NMR - 400.16 MHz - DMSO-d6

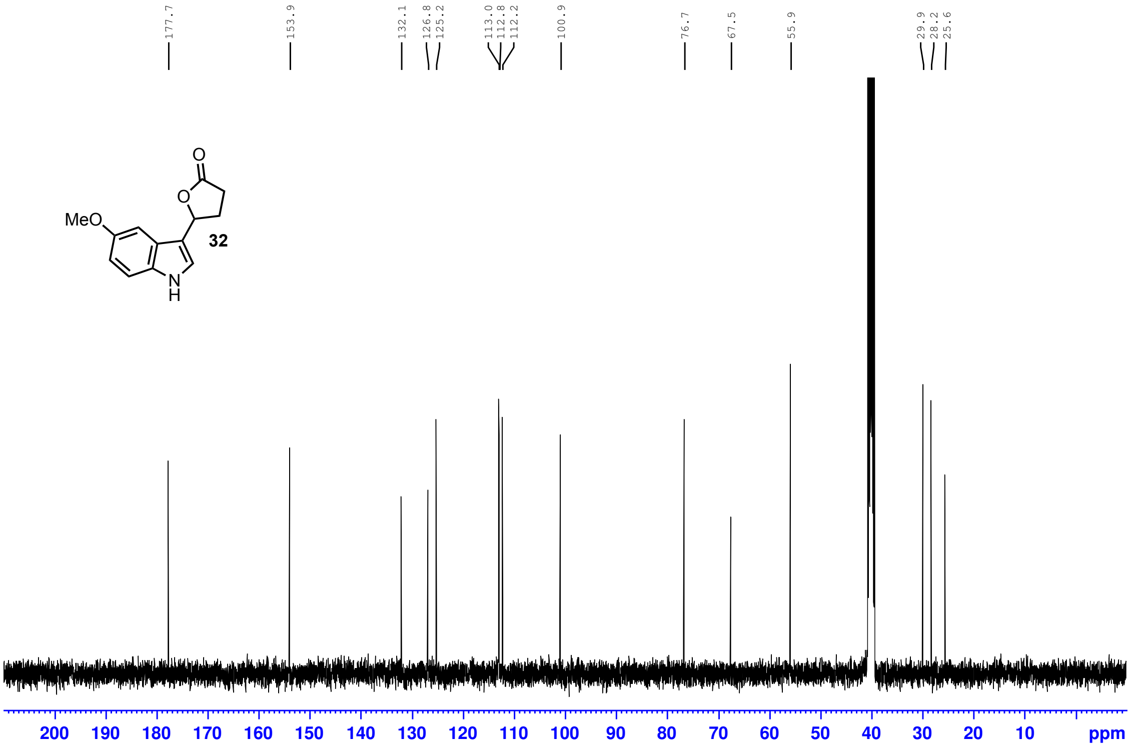

CN_3_56 - 13C NMR - 100.62 MHz - DMSO-d6

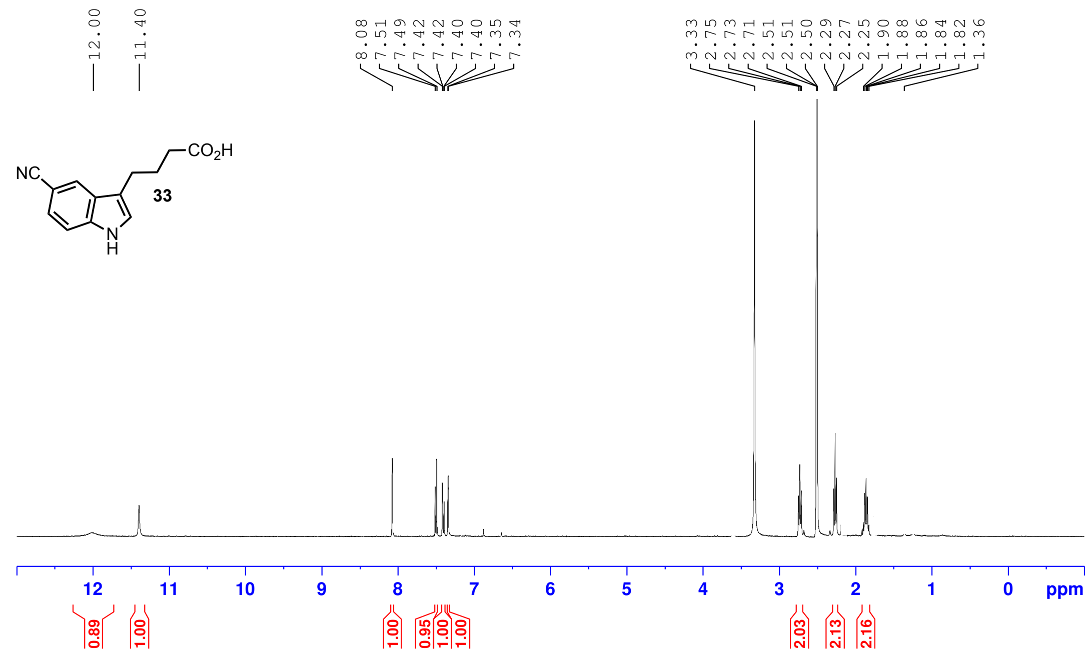

CN_3_45 - 1H NMR - 400.16 MHz - DMSO-d6

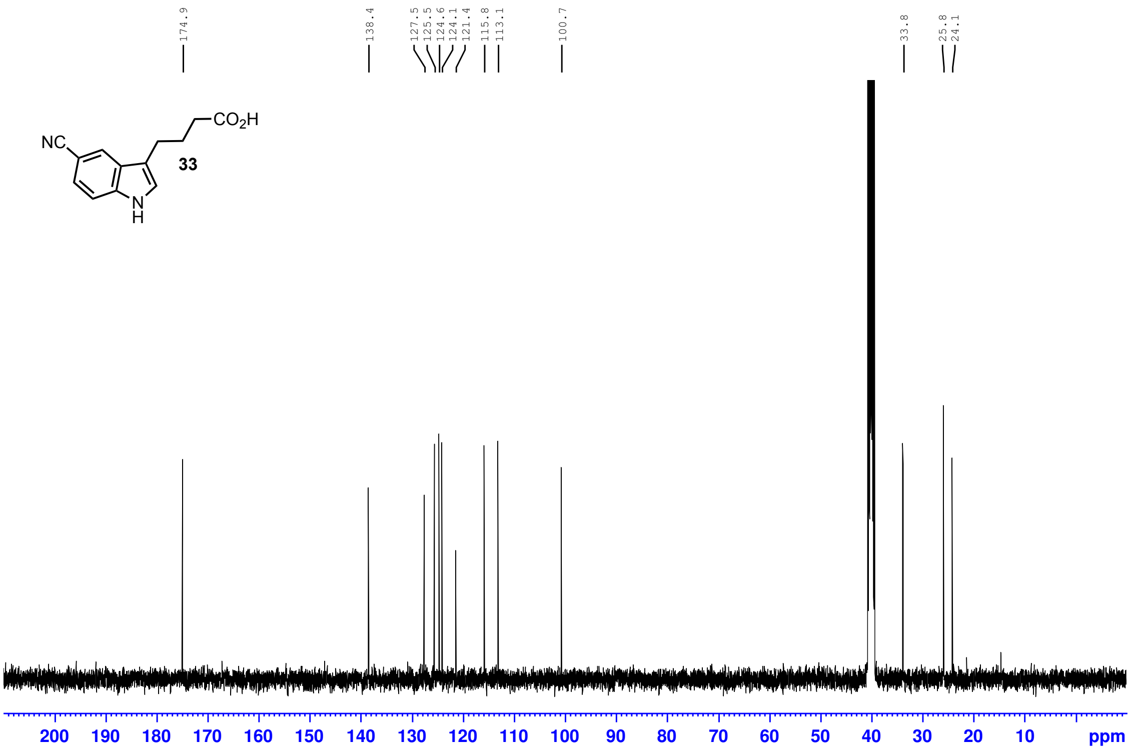

CN_3_45 - 13C NMR - 100.62 MHz - DMSO-d6

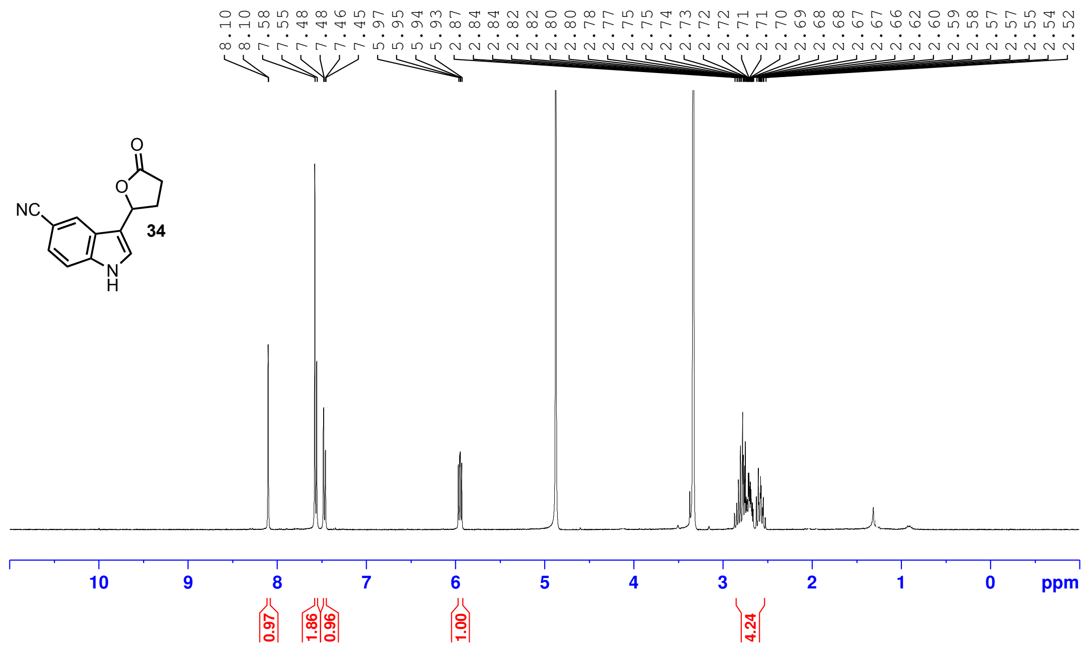

CN_3_48 - 1H NMR - 400.16 MHz - MeOD

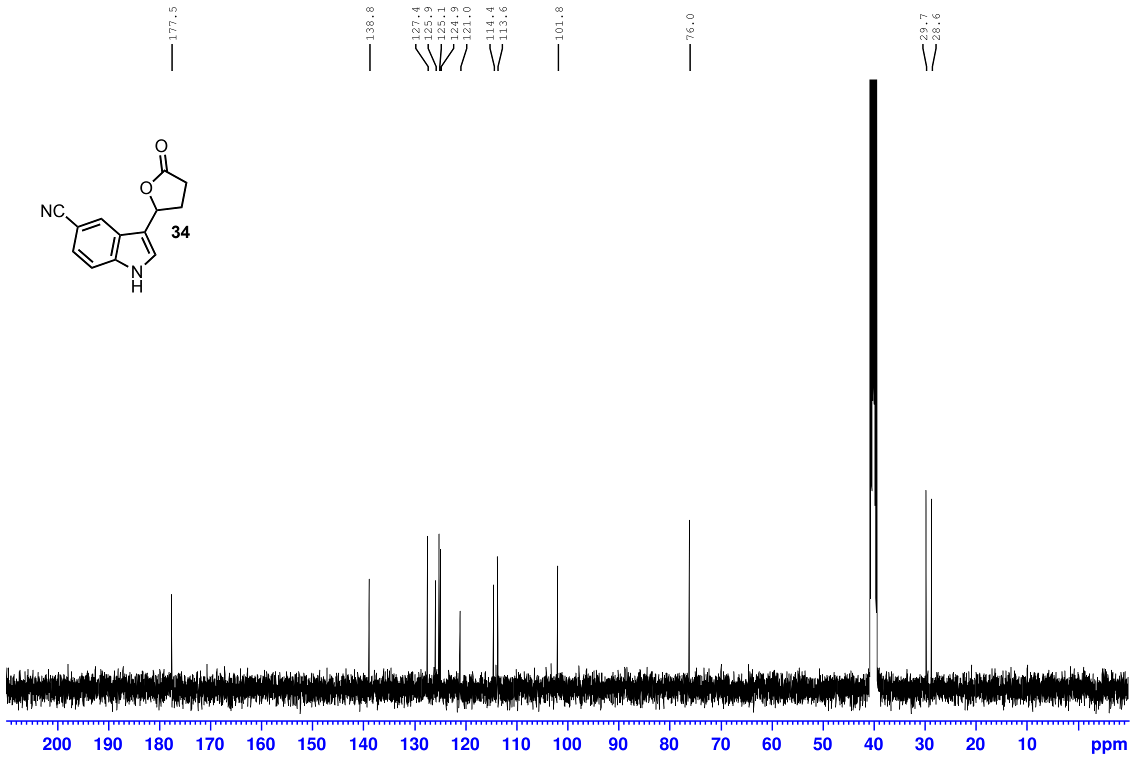

CN-3-48-13C - 13C NMR - 100.62 MHz - DMSO-d6

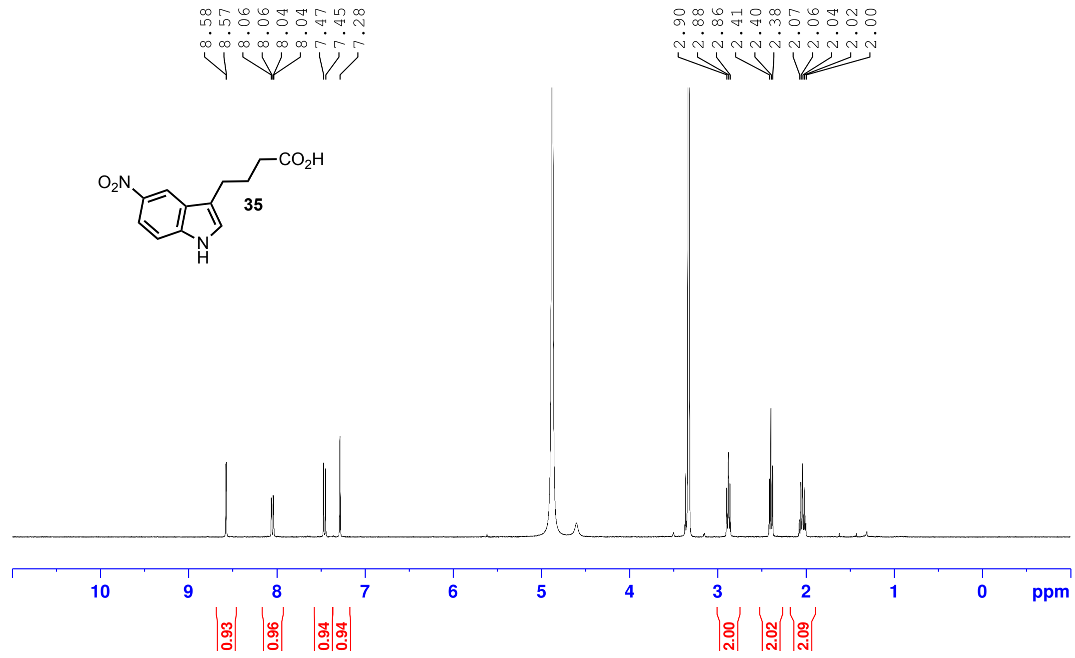

CN_3_83 - 1HNMR - 400.16 MHz - MeOD

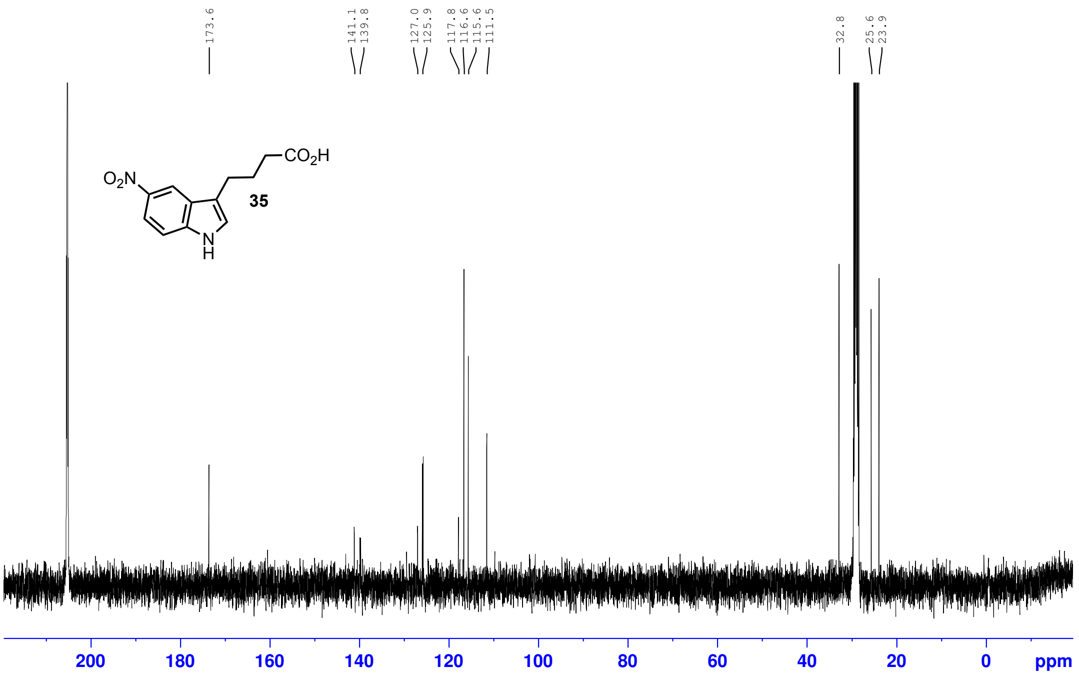

CN_3_83 - 13CNMR - 100.62 MHz - Acetone-d6

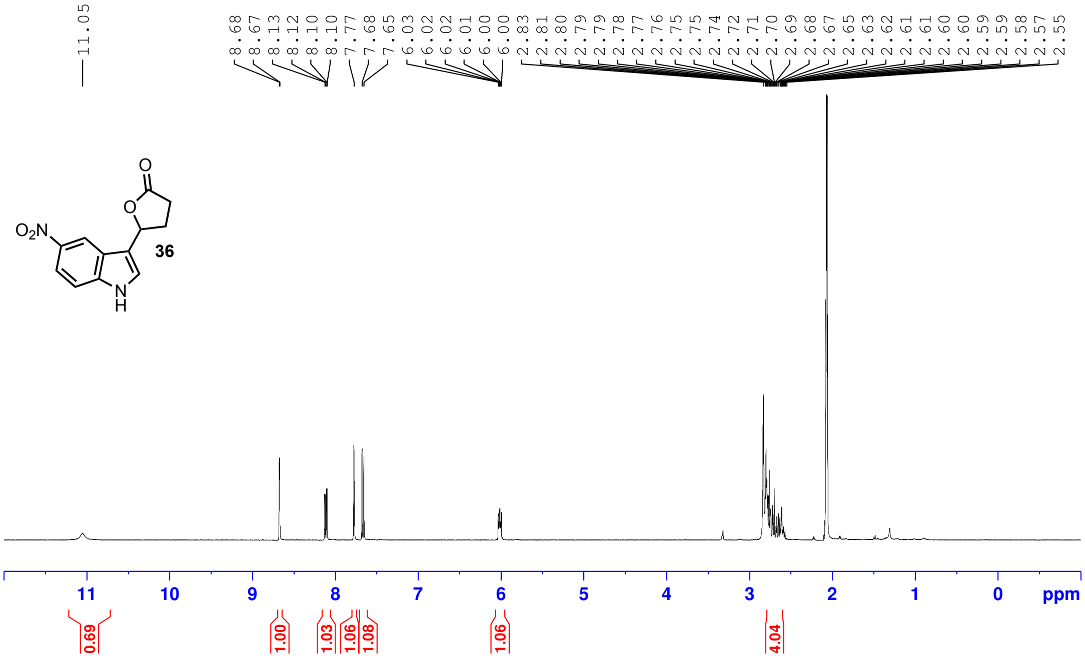

CN_3_85 - 1H NMR - 400.16 MHz - Acetone-d6

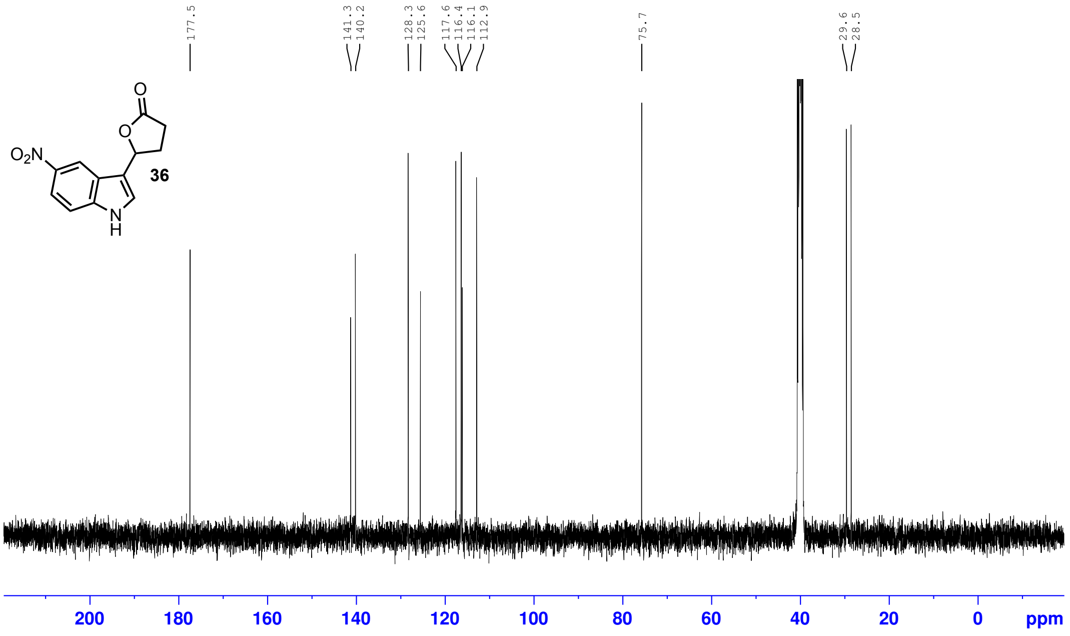

CN_3_85 - 13CNMR - 100.62 MHz - DMSO-d6

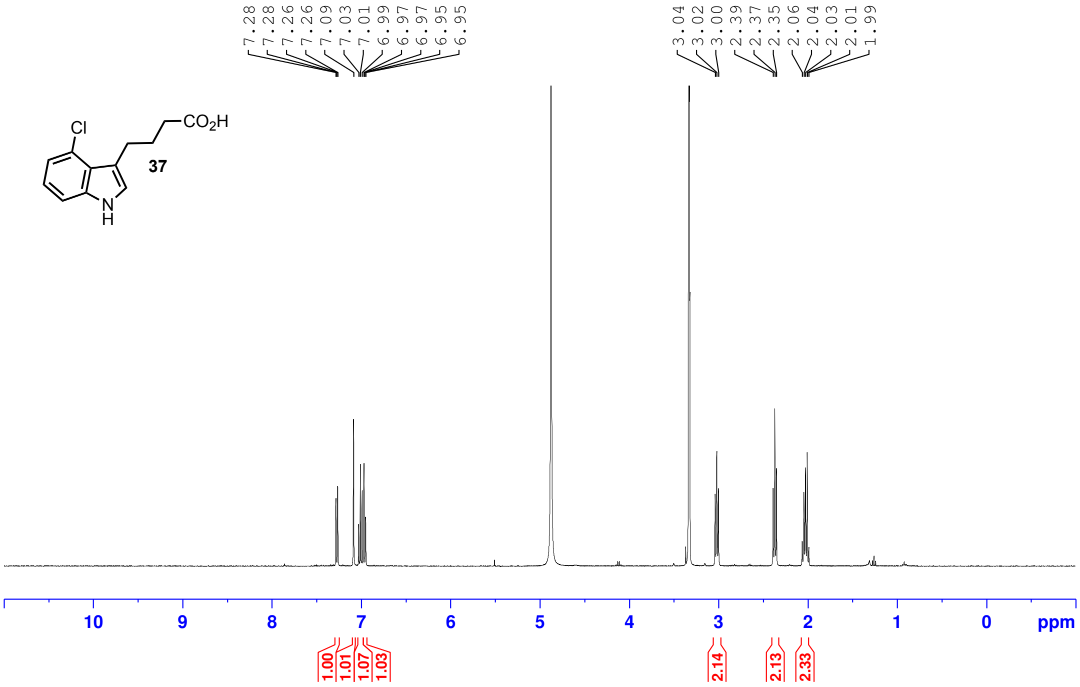

CN_3_37 - 1H NMR - 400.16 MHz - MeOD

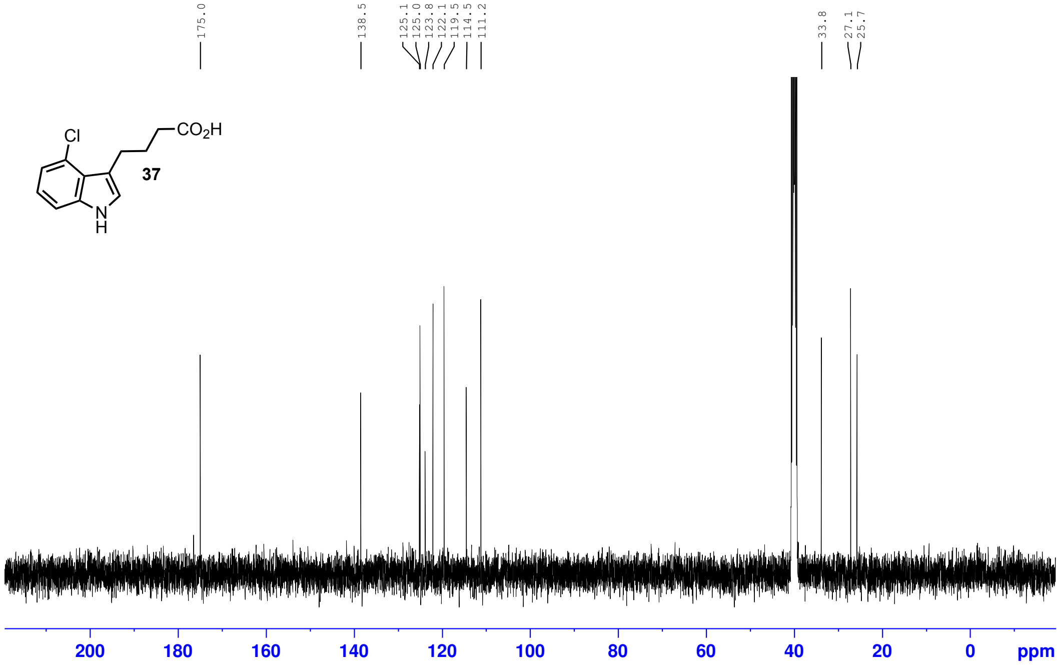

CN_3_37 - 13C NMR - 100.62 MHz - DMSO-d6

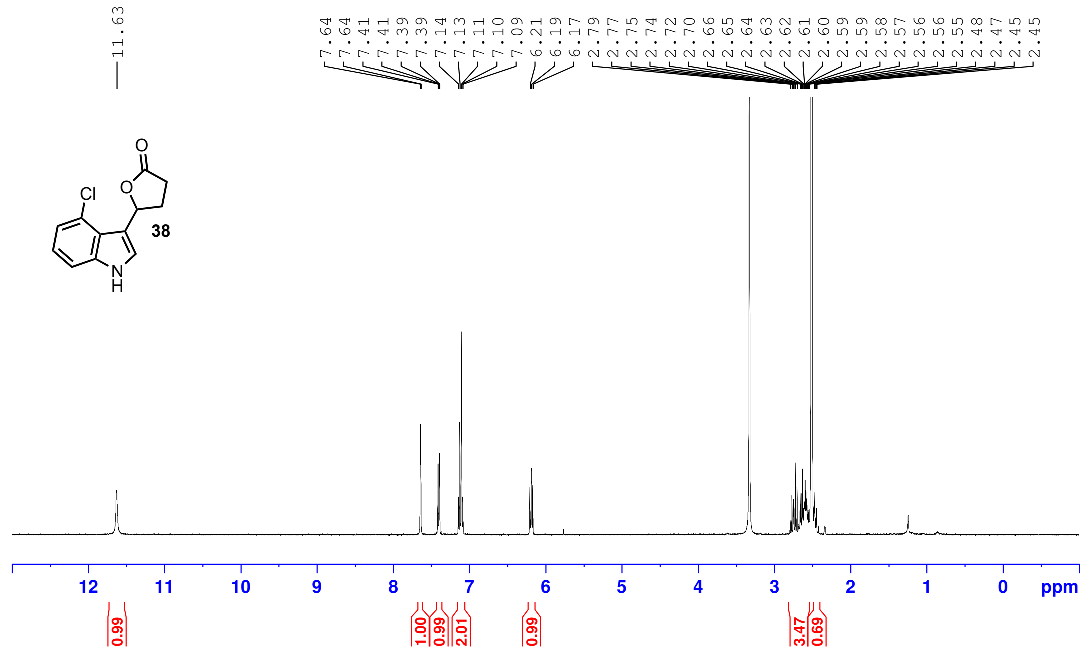

CN_3_41 - 1H NMR - 400.16 MHz - DMSO-d6

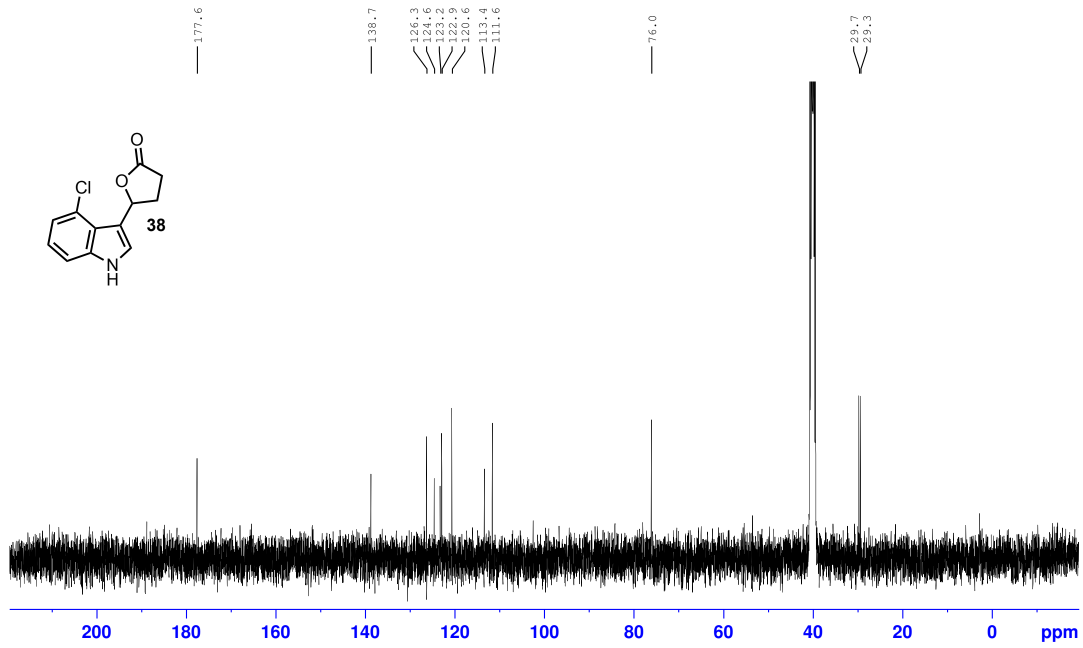

CN_3_41 - 13C NMR - 100.62 MHz - DMSO-d6

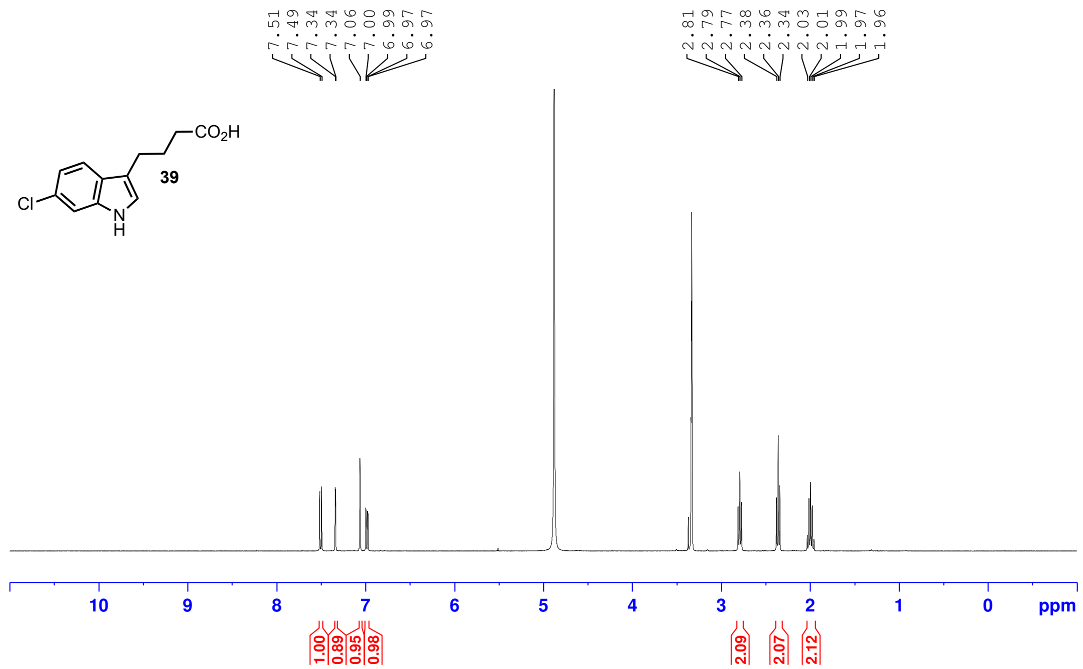

CN_3_26 - 1H NMR - 400.16 MHz - MeOD

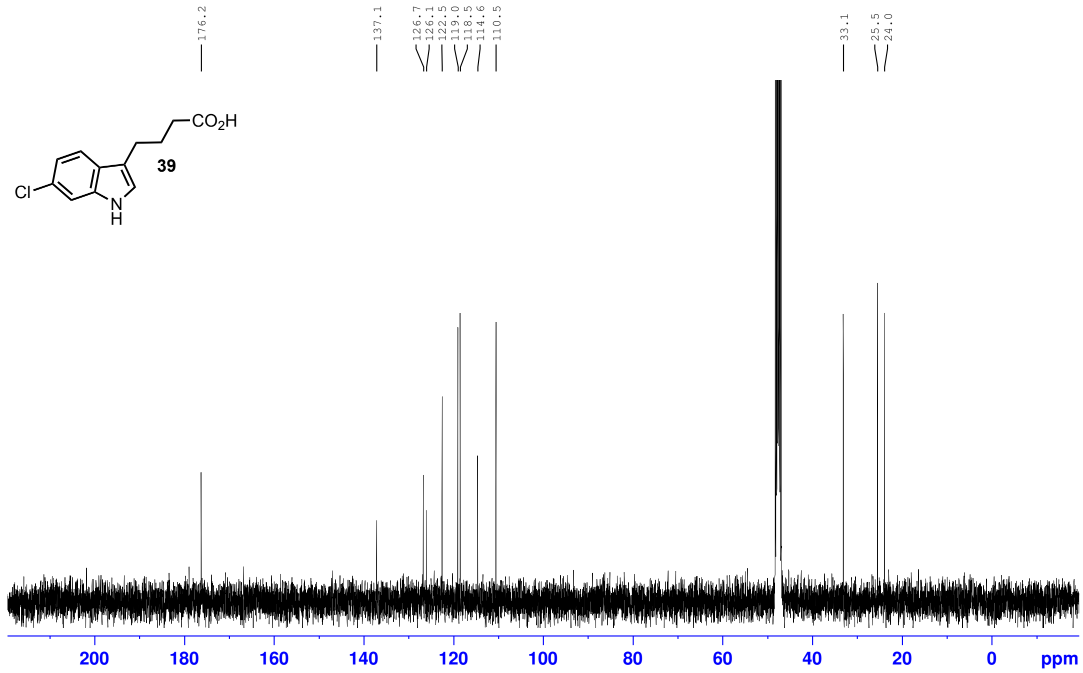

CN_3_26 - 13C NMR - 100.62 MHz - MeOD

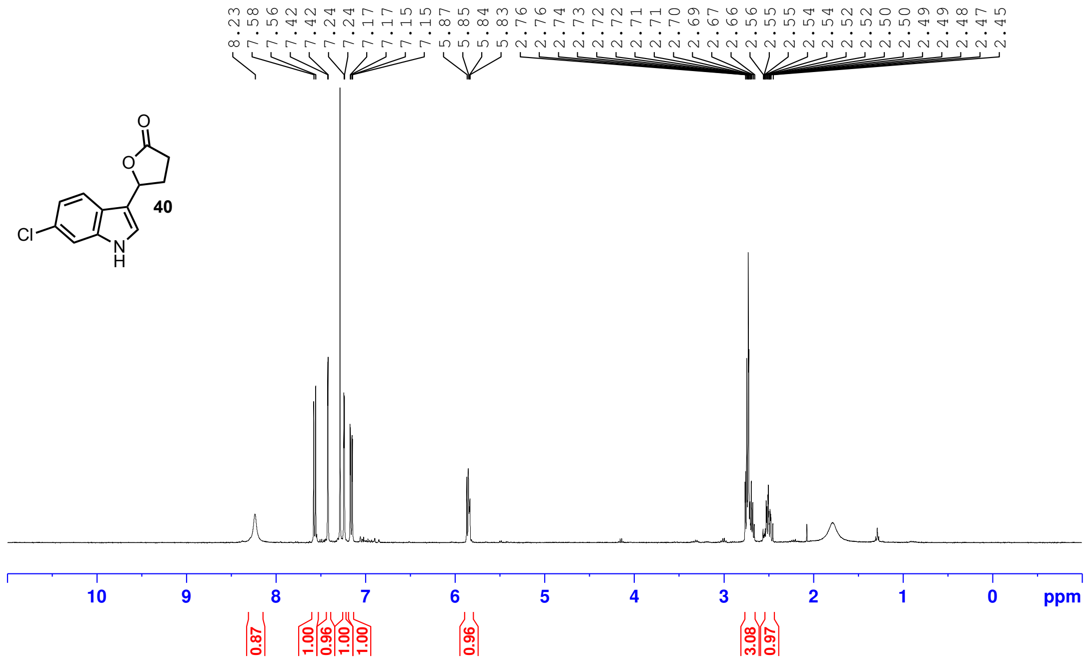

CN_3_33 - 1H NMR - 400.16 MHz - CDCl3

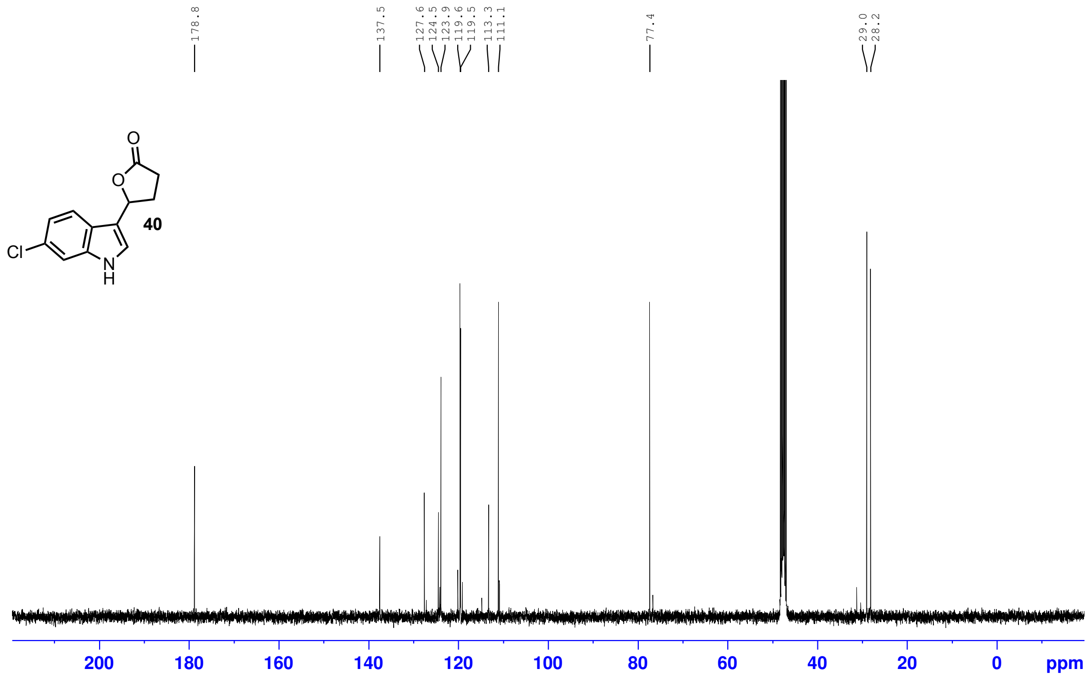

CN_3_33 - 13C NMR - 100.62 MHz - MeOD

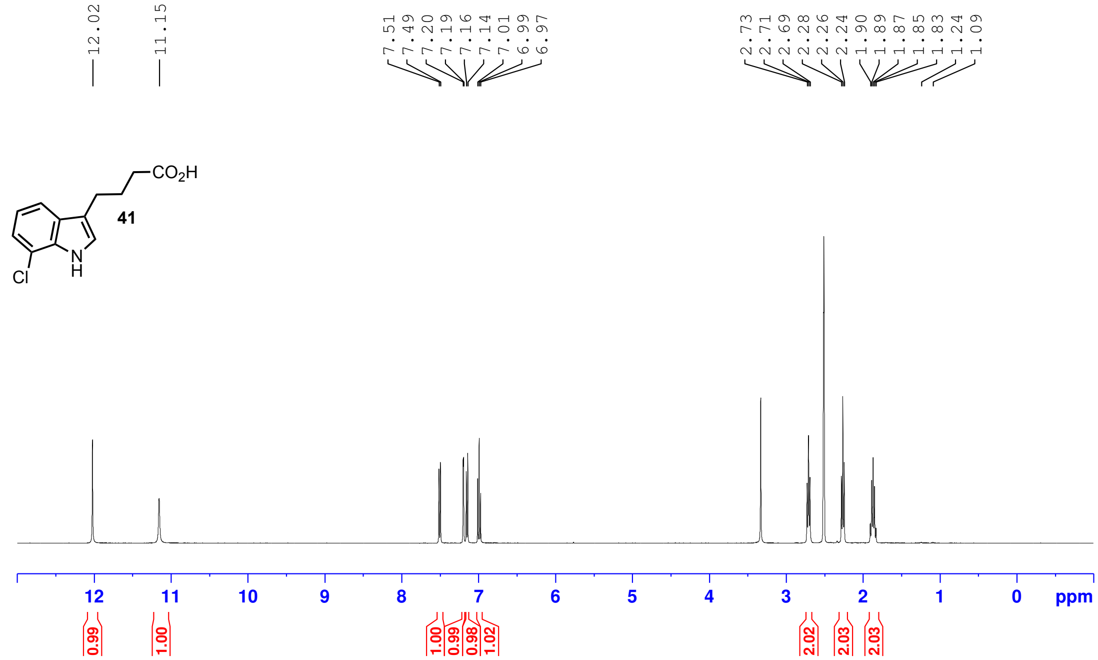

CN_3_61 - 1H NMR - 400.16 MHz - DMSO-d6

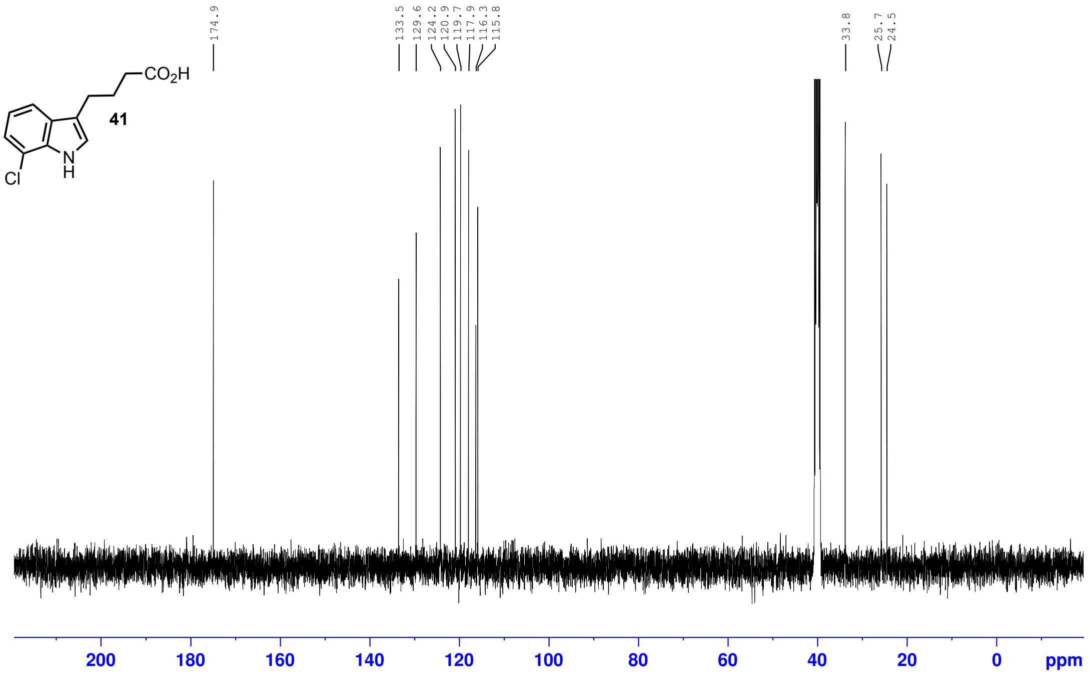

CN_3_61 - 13C NMR - 100.62 MHz - DMSO-d6

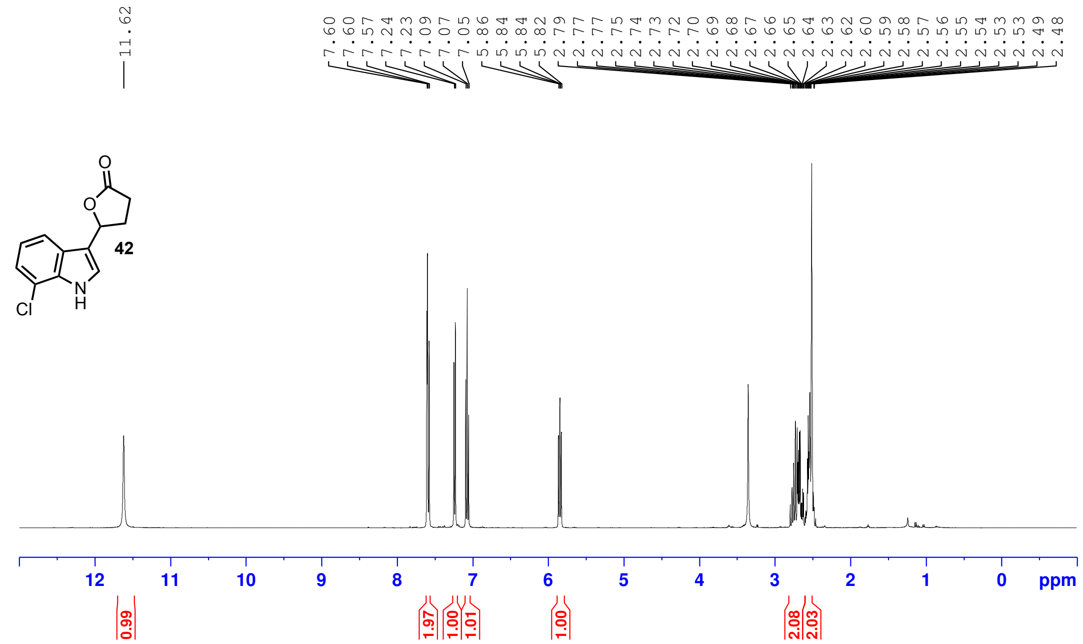

CN_3_62 - 13C NMR - 400.16 MHz - DMSO-d6

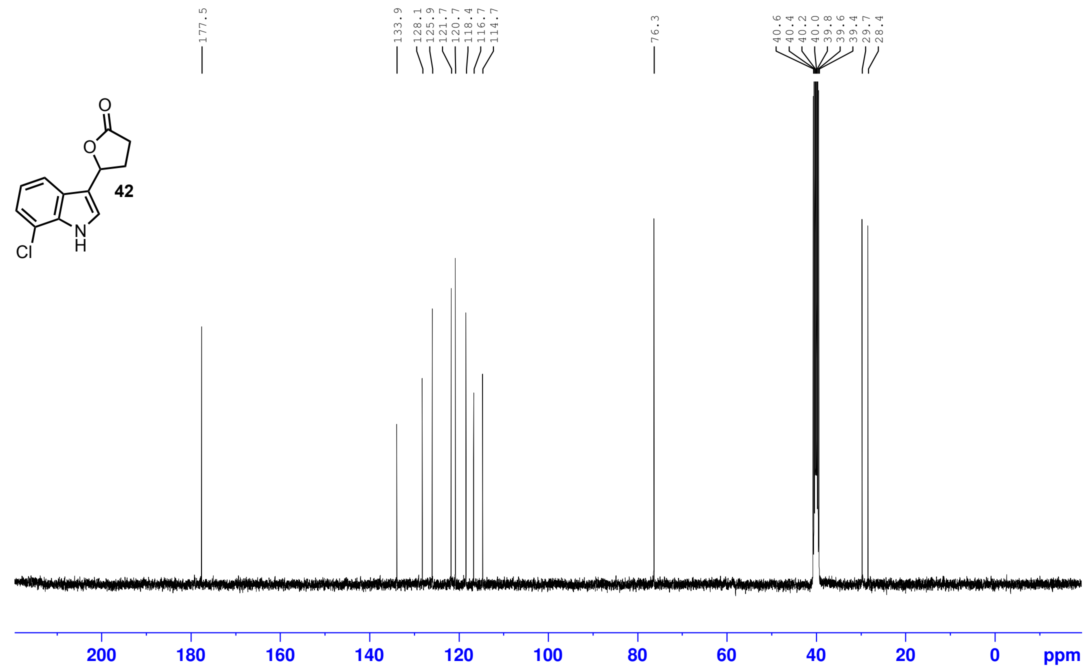

CN_3_62 - 13C NMR - 100.62 MHz - DMSO-d6

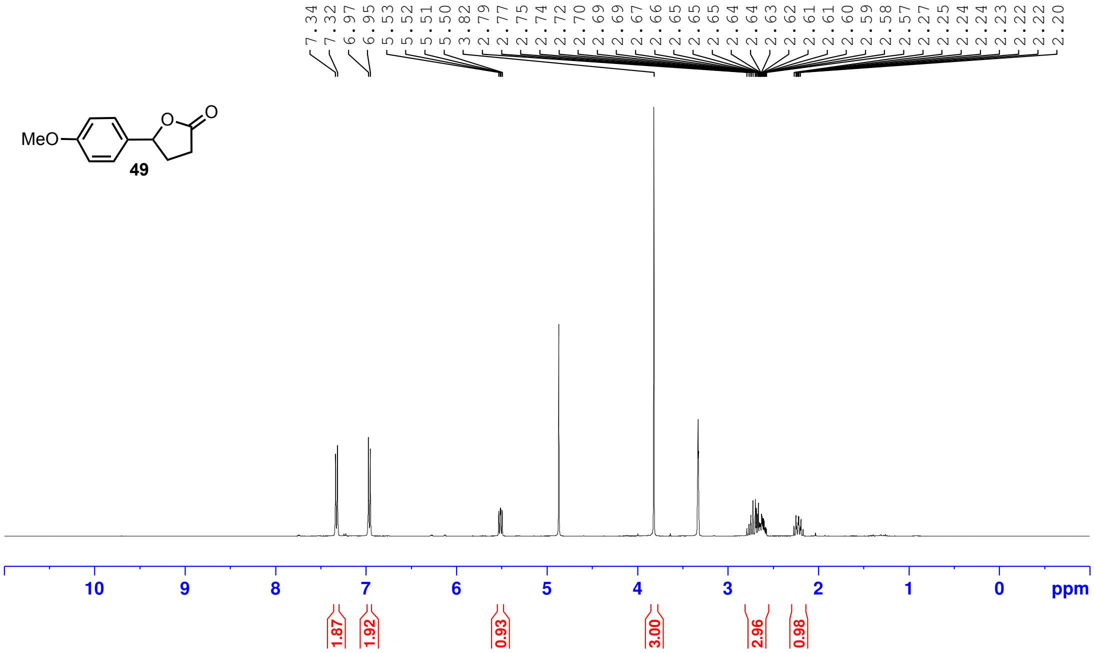

CN_2_128 - 1H NMR - 400.13 MHz - MeOD

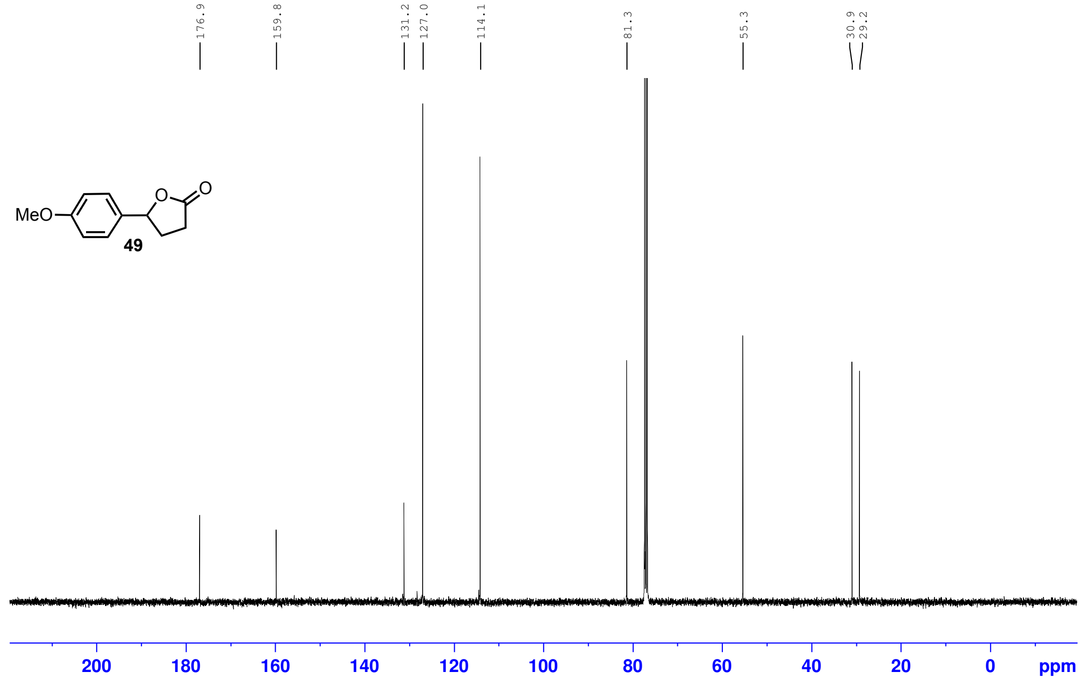

CN_2_128 - 13C NMR - 100.62 MHz - CDCl3

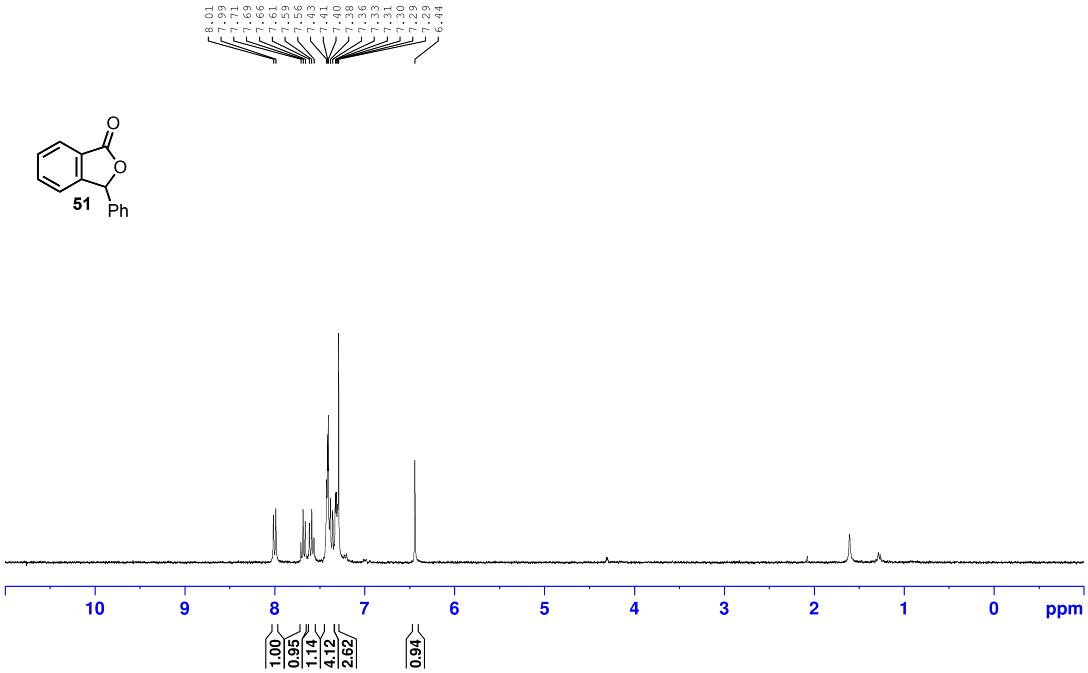

CN_2_123 - 1H NMR - 400.16 MHz - CDCl3

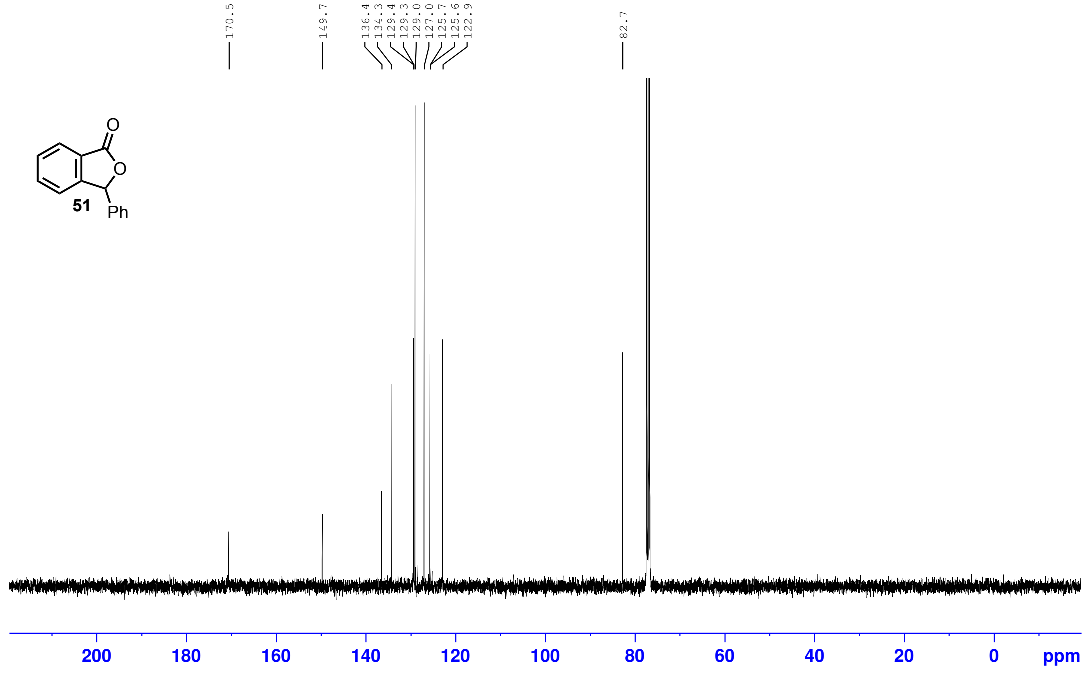

CN_2_123 - 13C NMR - 100.62 MHz - CDCl3

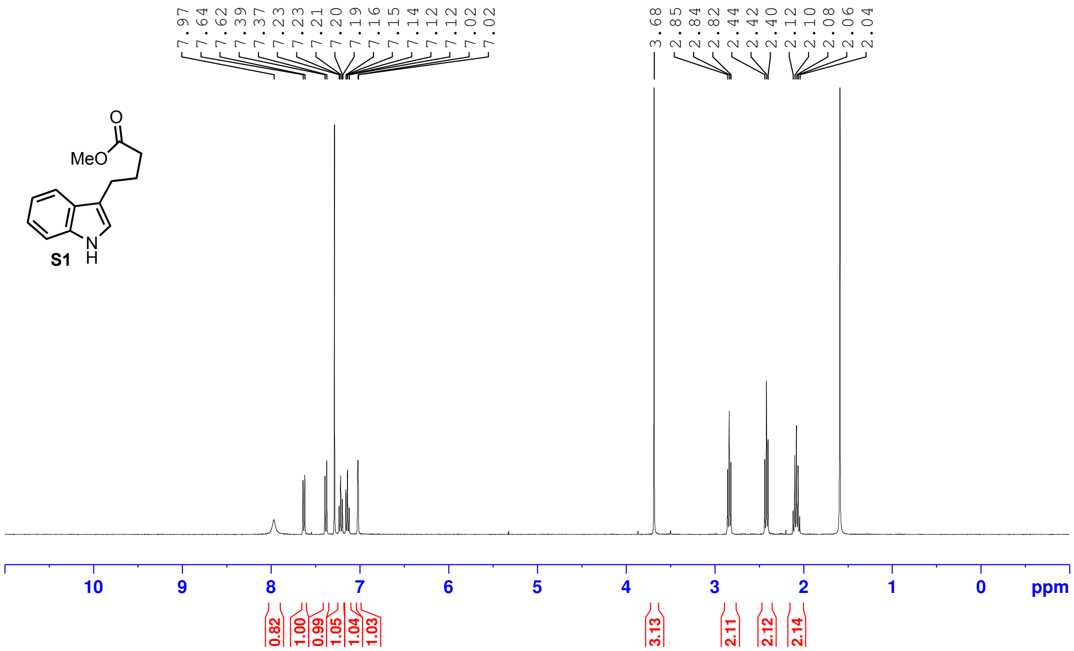

CN_2_29 - 1H NMR - 400.16 MHz - CDCl3

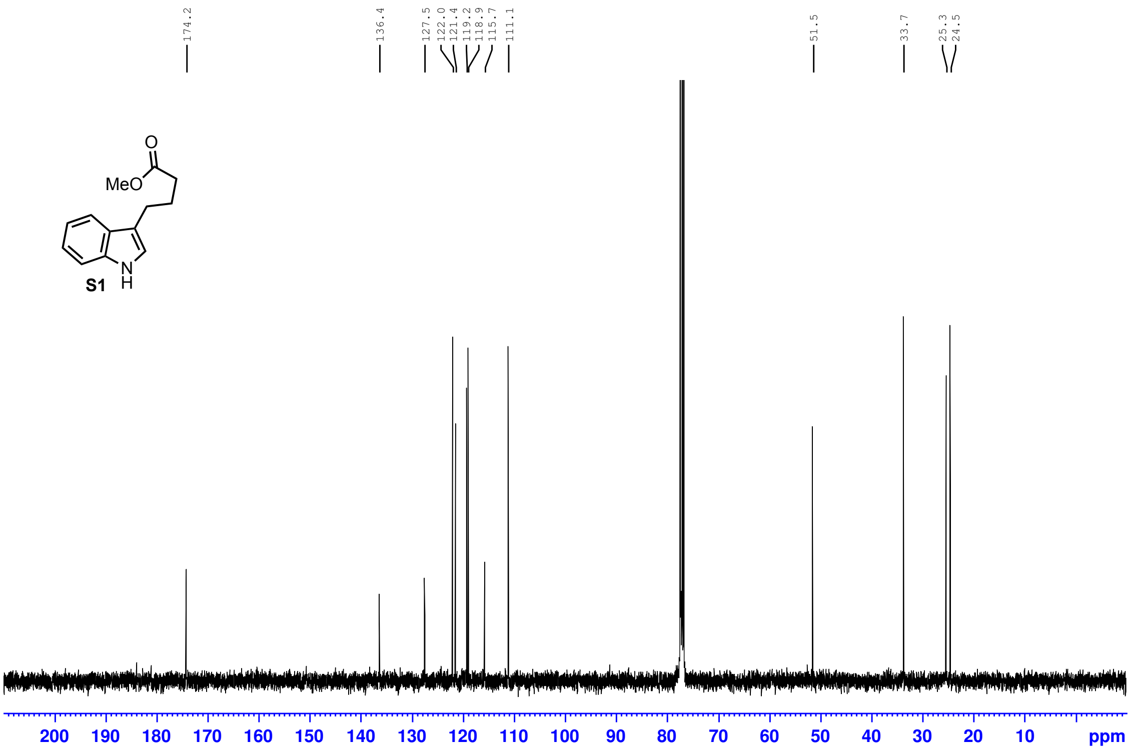

CN_2_29 - 13C NMR - 100.62 MHz - CDCl3

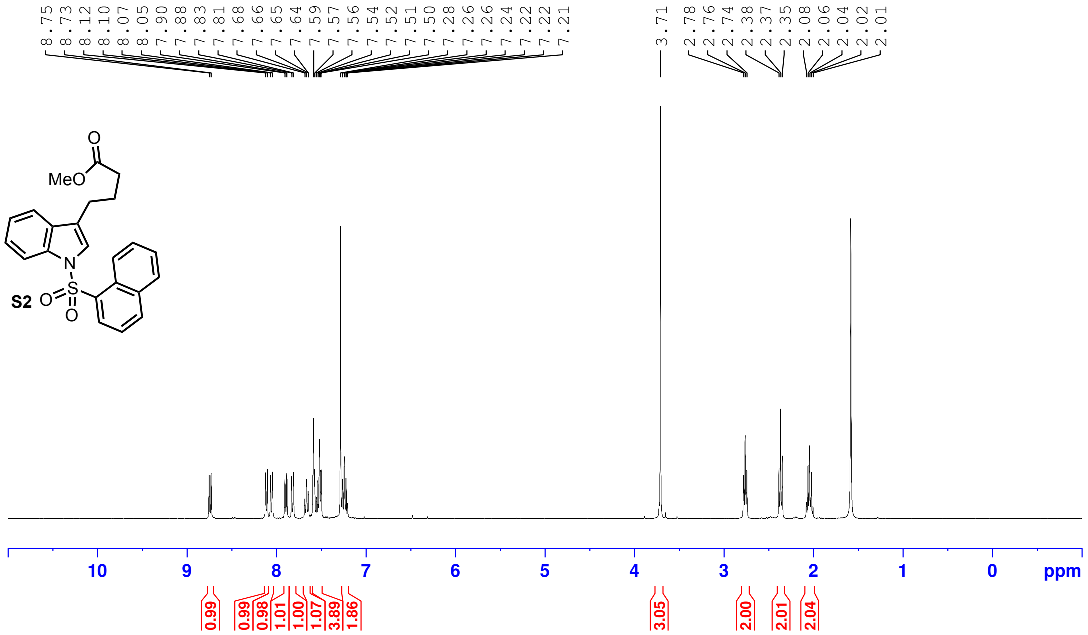

CN_3_95_1HNMR_400.16 MHz_CDCl3

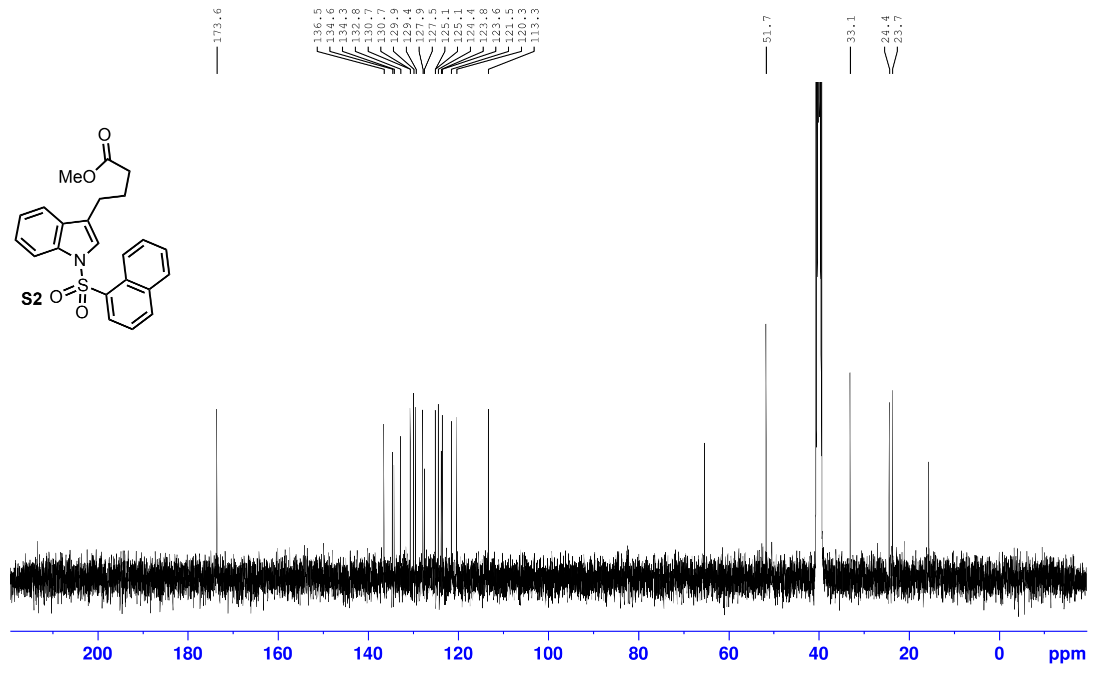

CN_3_95 - 13C NMR - 100.62 MHz - DMSO-d6

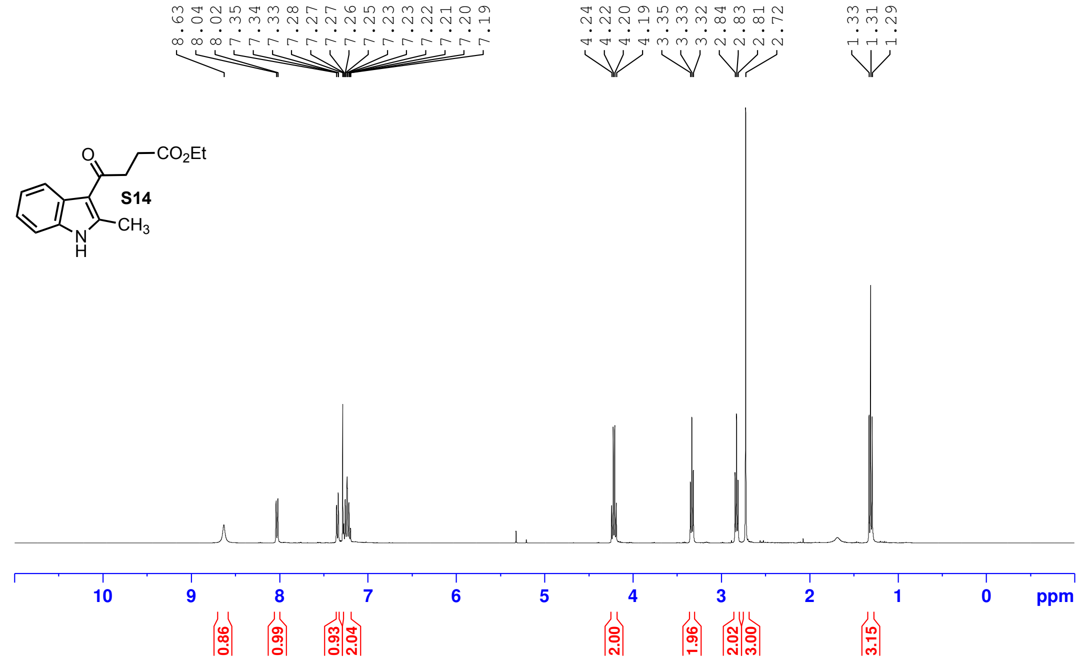

CN_3_5 - 1H NMR - 400.16 MHz - CDCl3

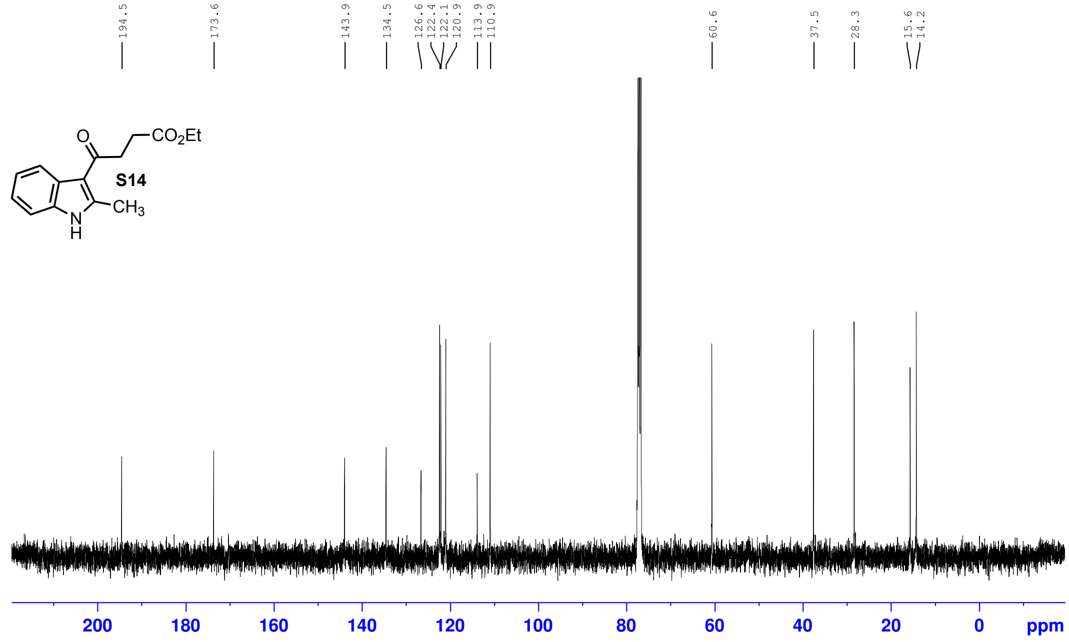

CN_3_5 -13C NMR - 100.62 MHz - CDCl3

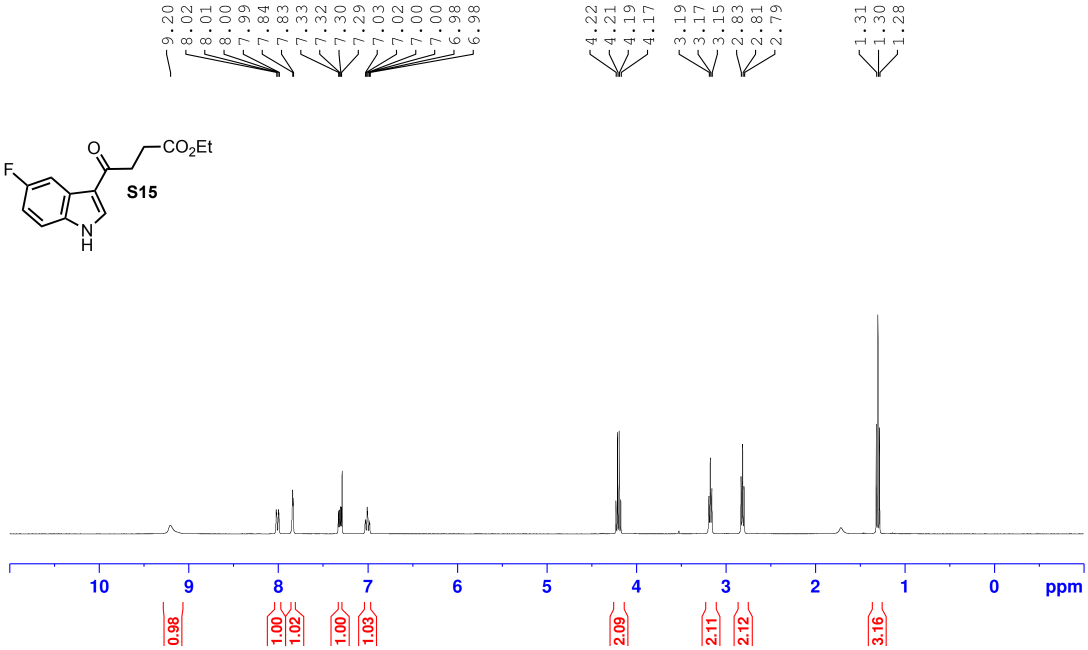

CN_3_44 - 1H NMR - 400.16 MHz - CDCl3

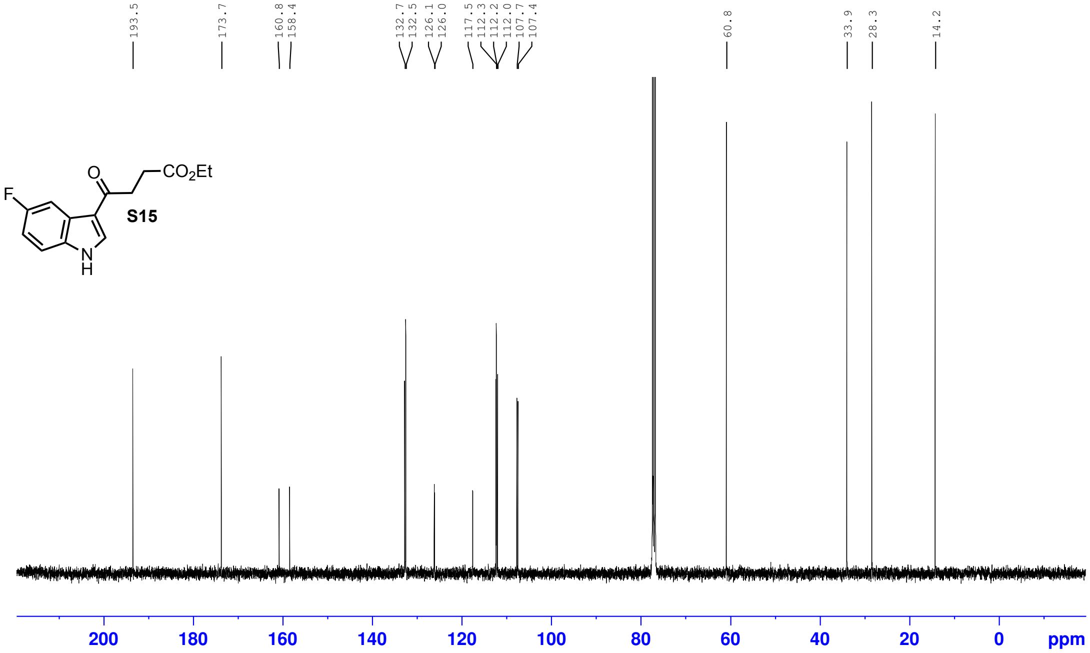

CN_3_44 - 13C NMR - 100.62 MHz - CDCl3

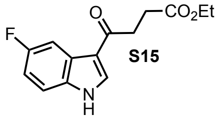

S15

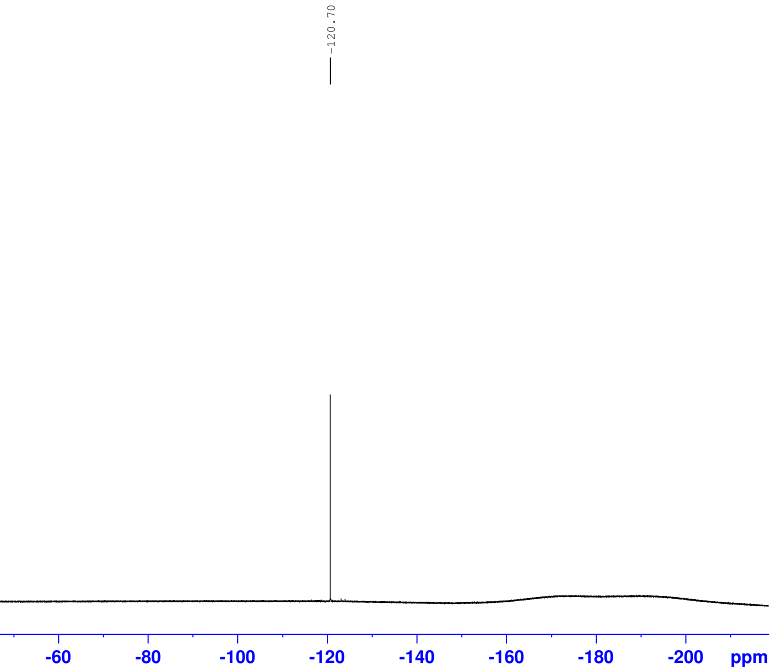

CN_3_44 - 19F NMR - 376.50 MHz - CDCl3

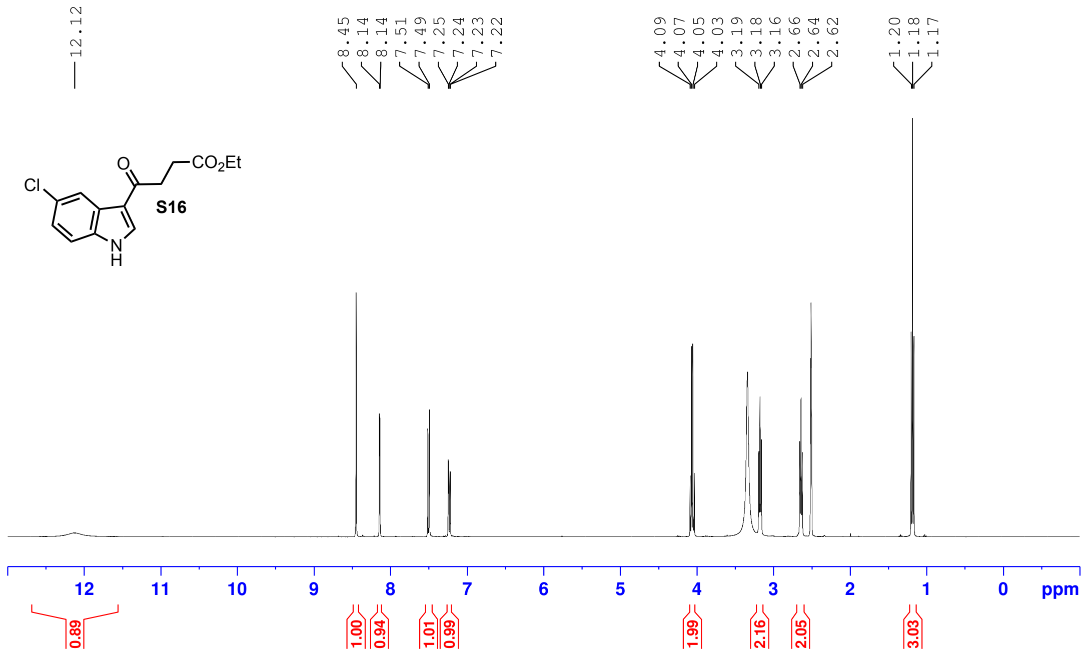

CN_3_13 - 1H NMR - 400.16 MHz - DMSO-d6

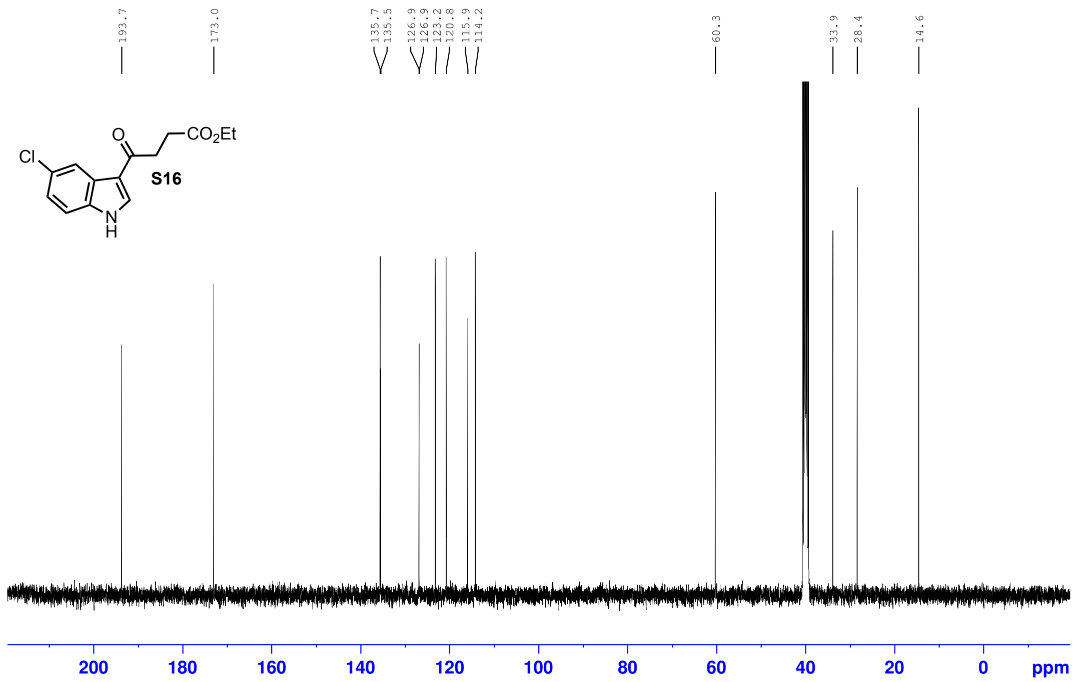

CN_3_13 - 13C NMR - 100.62 MHz - DMSO-d6

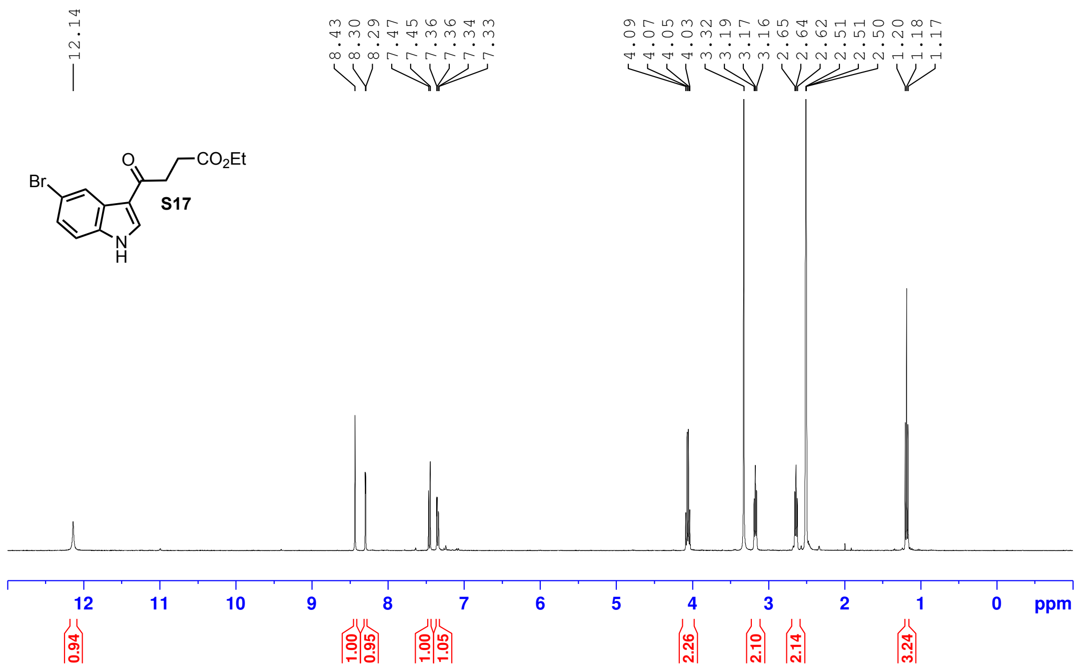

CN_2_84 - 1H NMR - 400.16 MHz - DMSO-d6

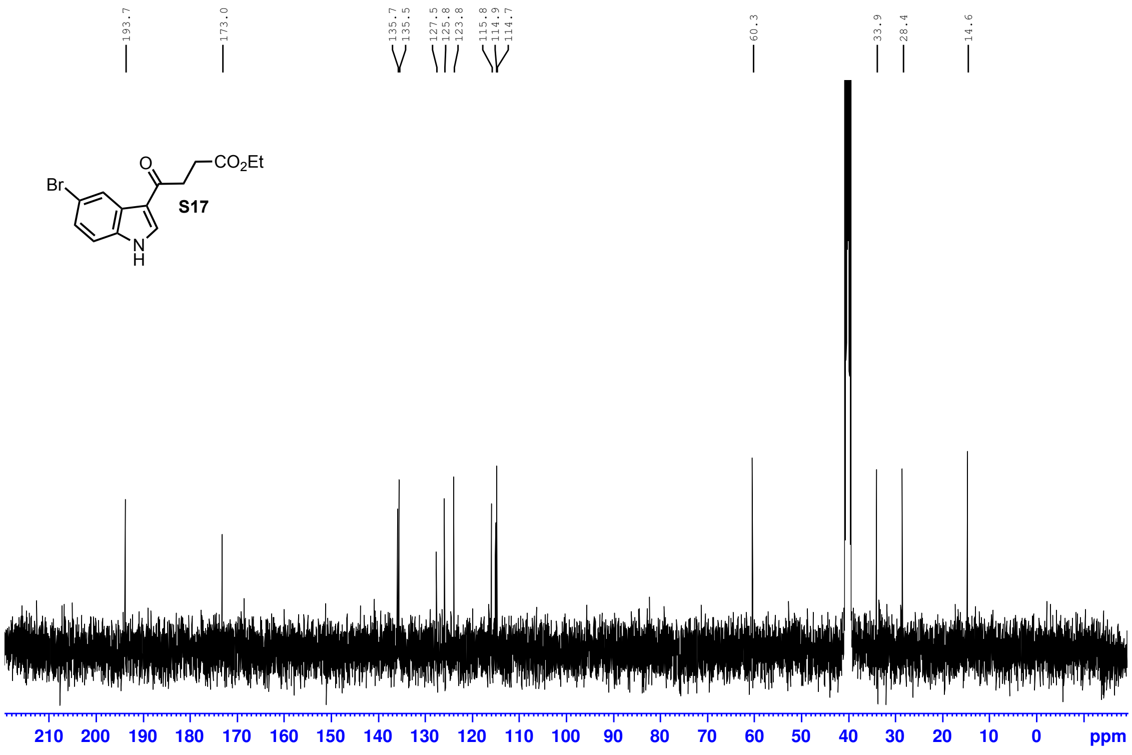

CN_2_84 - 13C NMR - 100.62 MHz - DMSO-d6

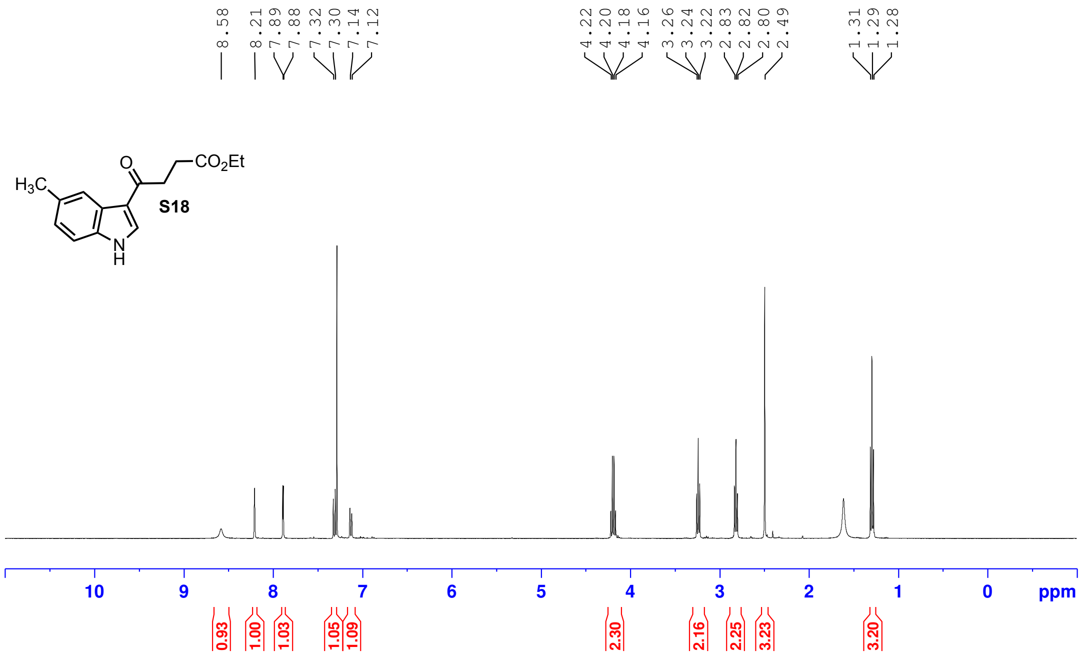

CN-3-64 - 1H NMR - CDCl3 - 400.16 MHz

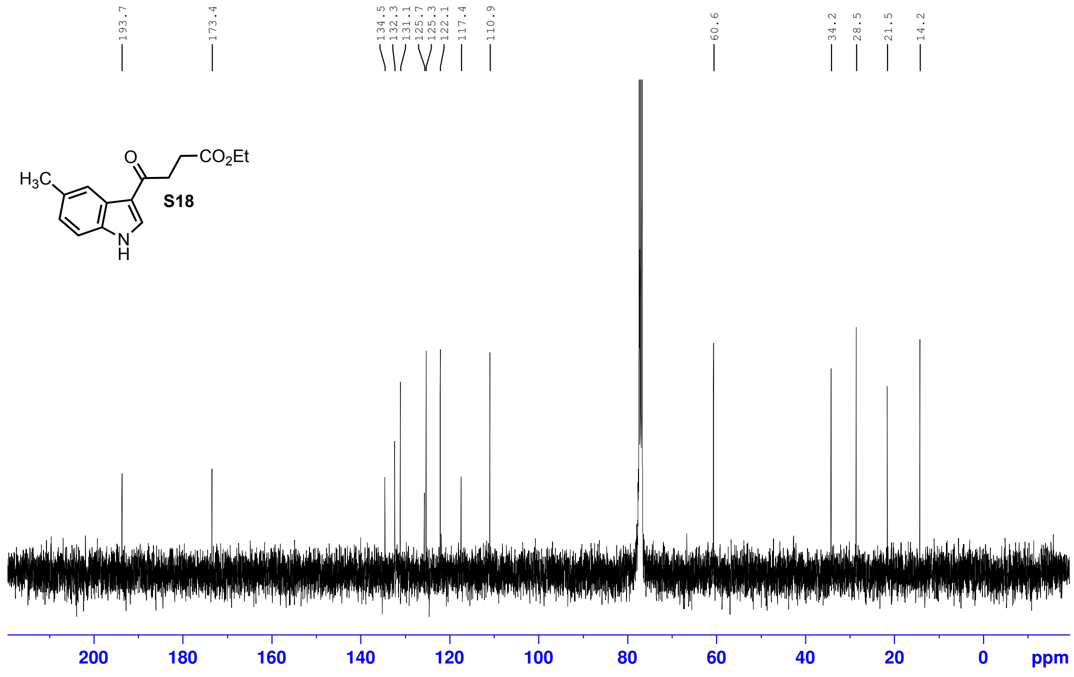

CN_3_64 - 13C NMR - CDCl3 - 100.62 MHz

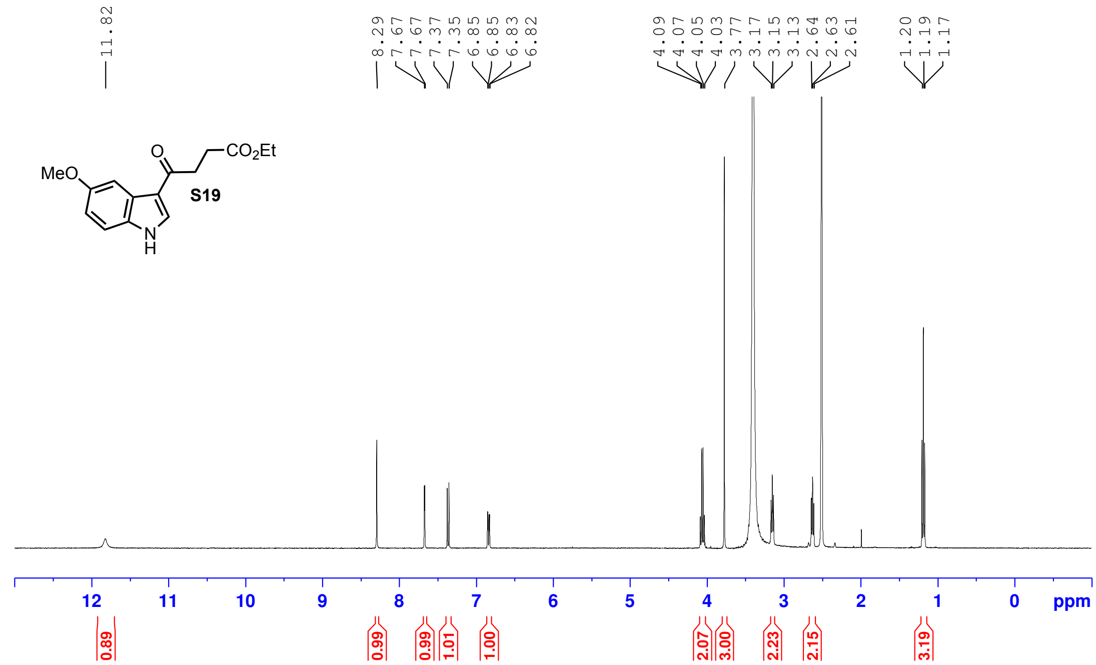

CN_3_49 - 1H NMR - 400.16 MHz - DMSO-d6

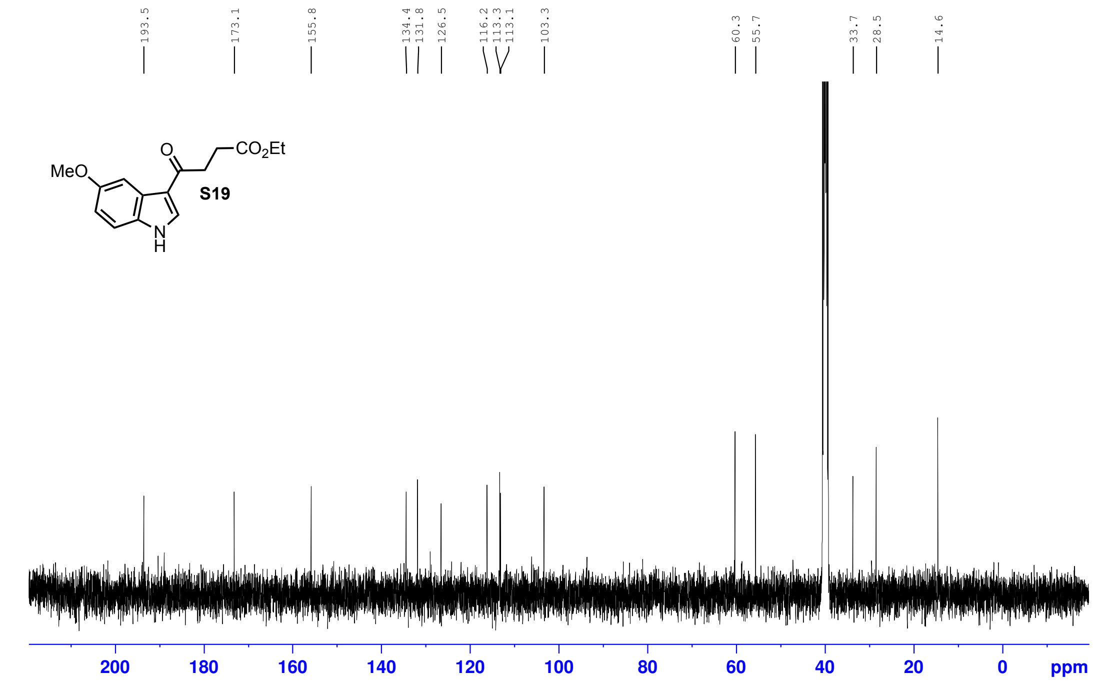

CN_3_49 - 13C NMR - 100.62 MHz - DMSO-d6

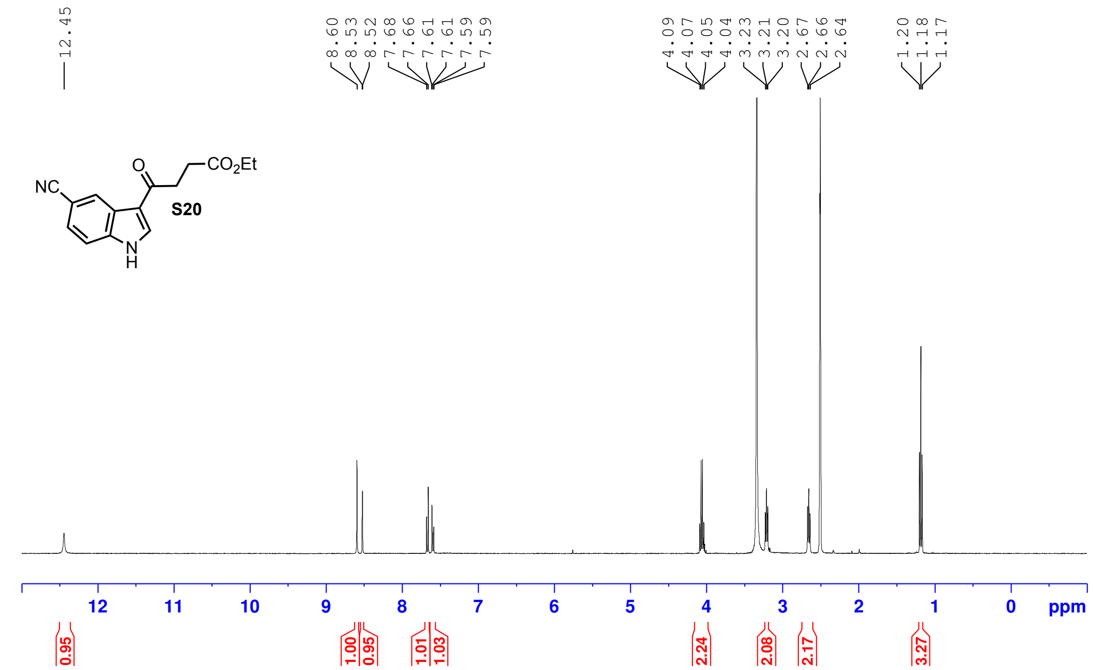

CN_3_36 - 1H NMR - 400.16 MHz - DMSO-d6

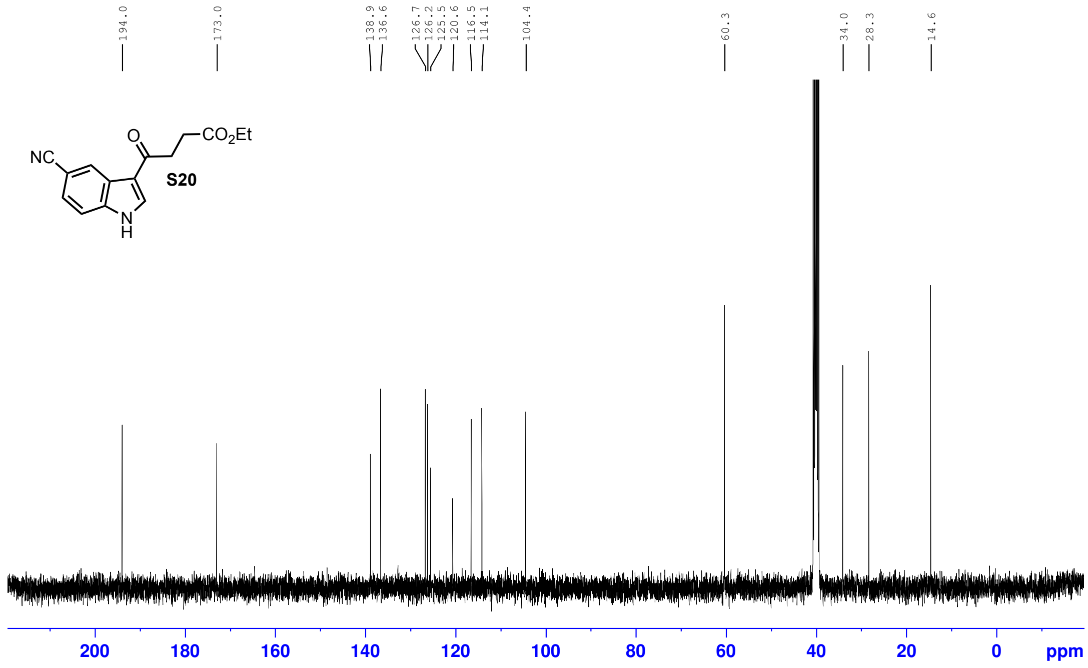

CN_3_36 - 13C NMR - 100.62 MHz - CDCl3

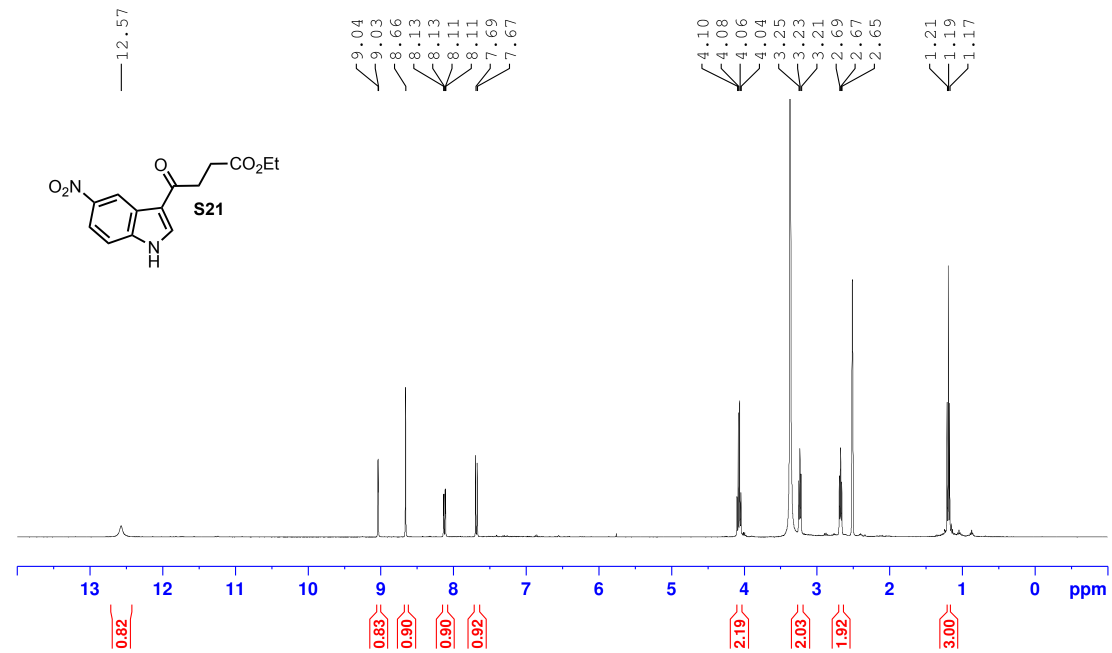

CN_3_65 - 1H NMR - 400.16 MHz - DMSO-d6

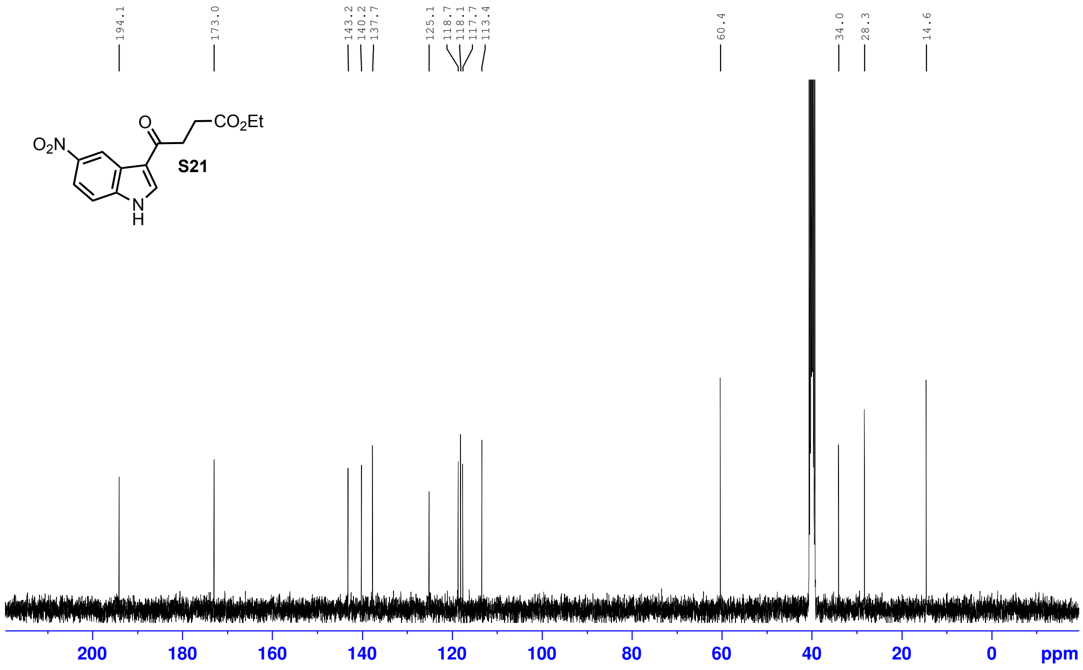

CN_3_65 - 13C NMR - 100.62 MHZ - DMSO-(

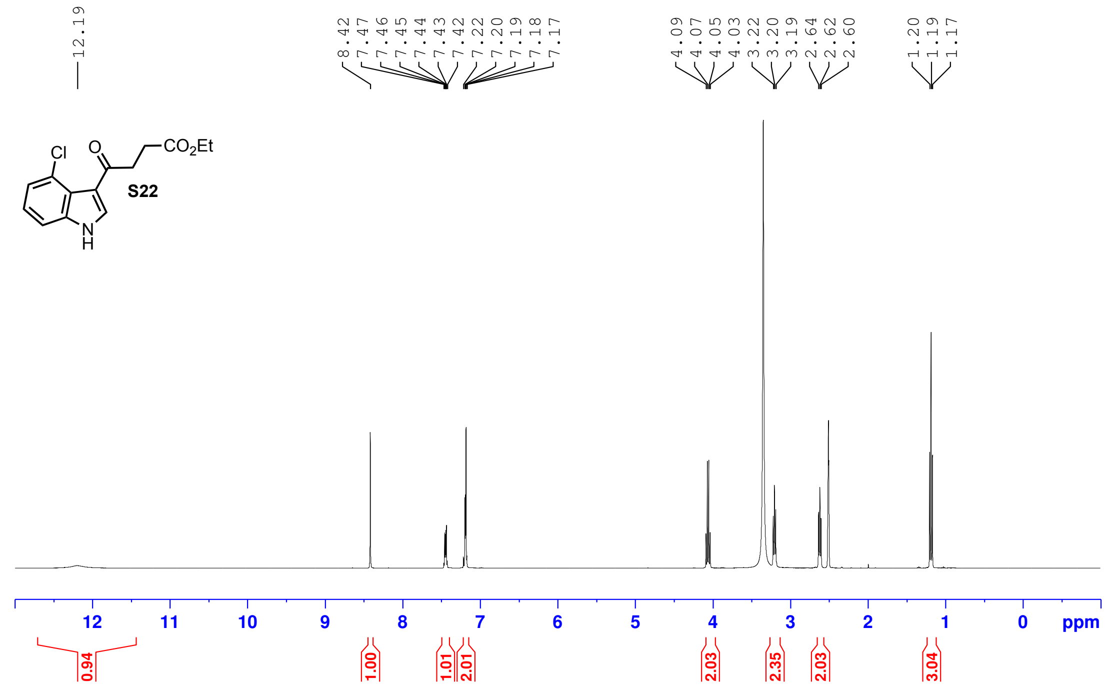

CN_3_31 - 1H NMR - 400.16 MHz - DMSO-d6

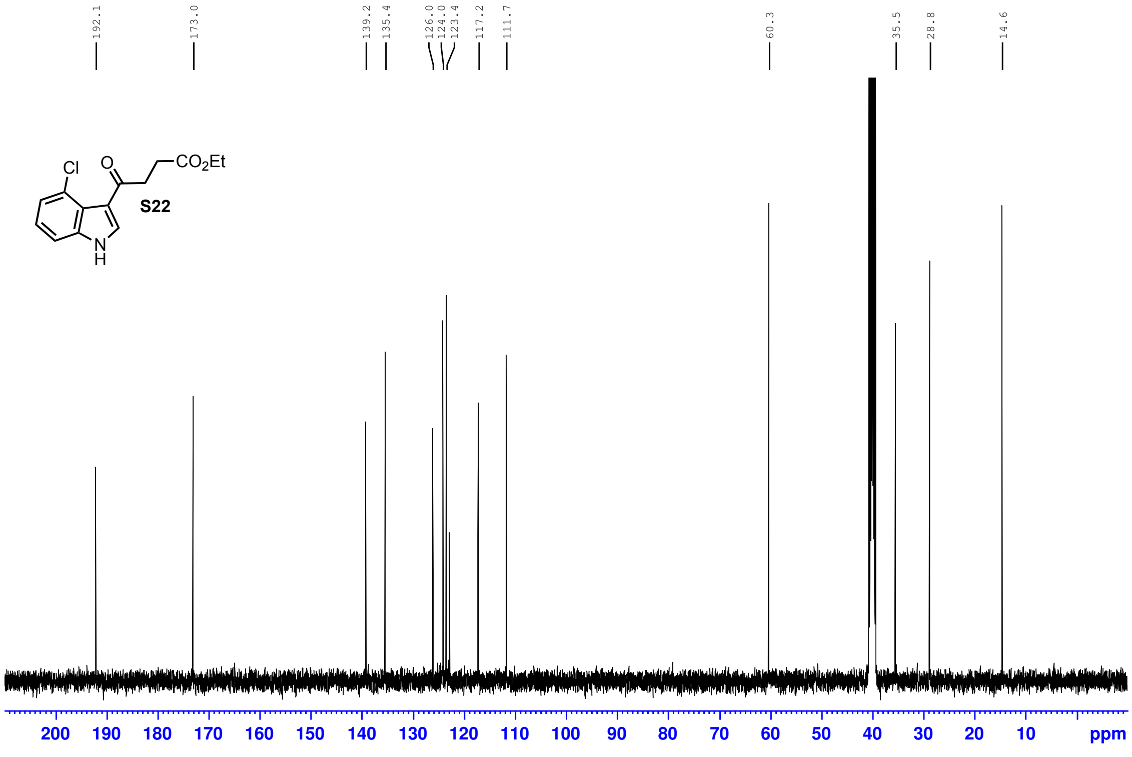

CN_3_31 - 13C NMR - 100.62 MHz - DMSO-d6

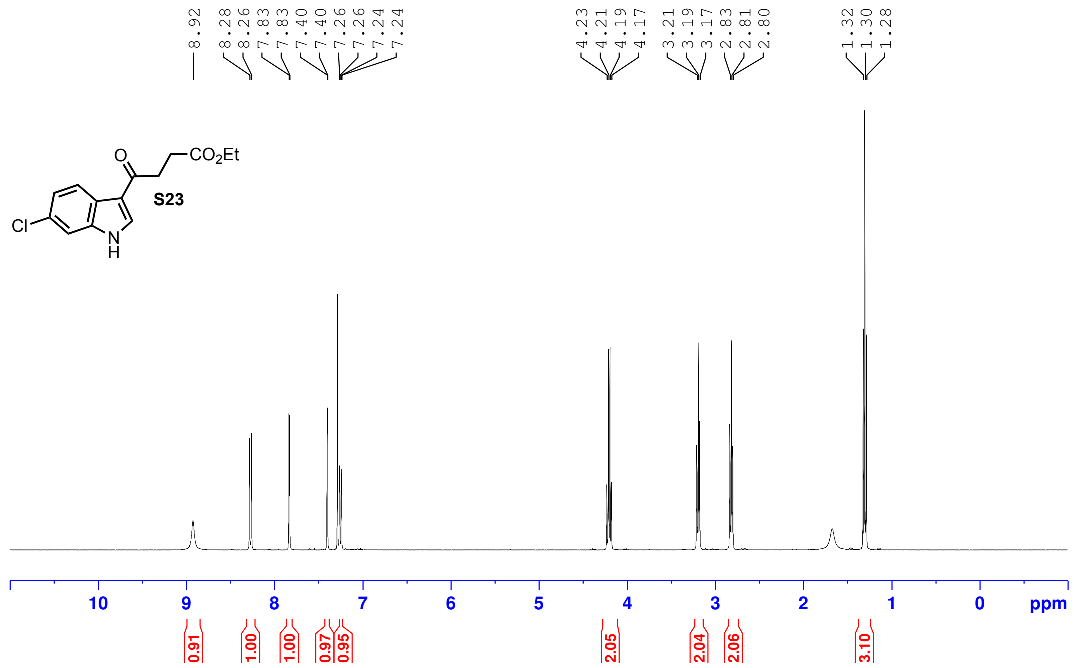

CN_3_22 - 1H NMR - 400.16 MHz - CDCl3

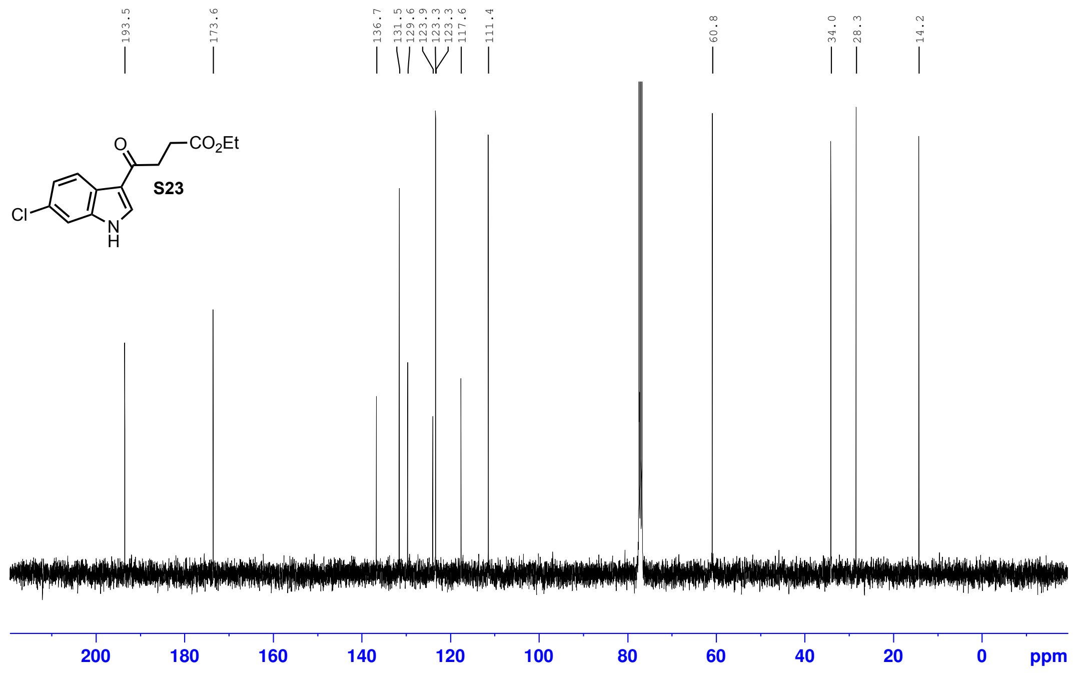

CN_3_22 - 13C NMR - 100.62 MHz - CDCl3

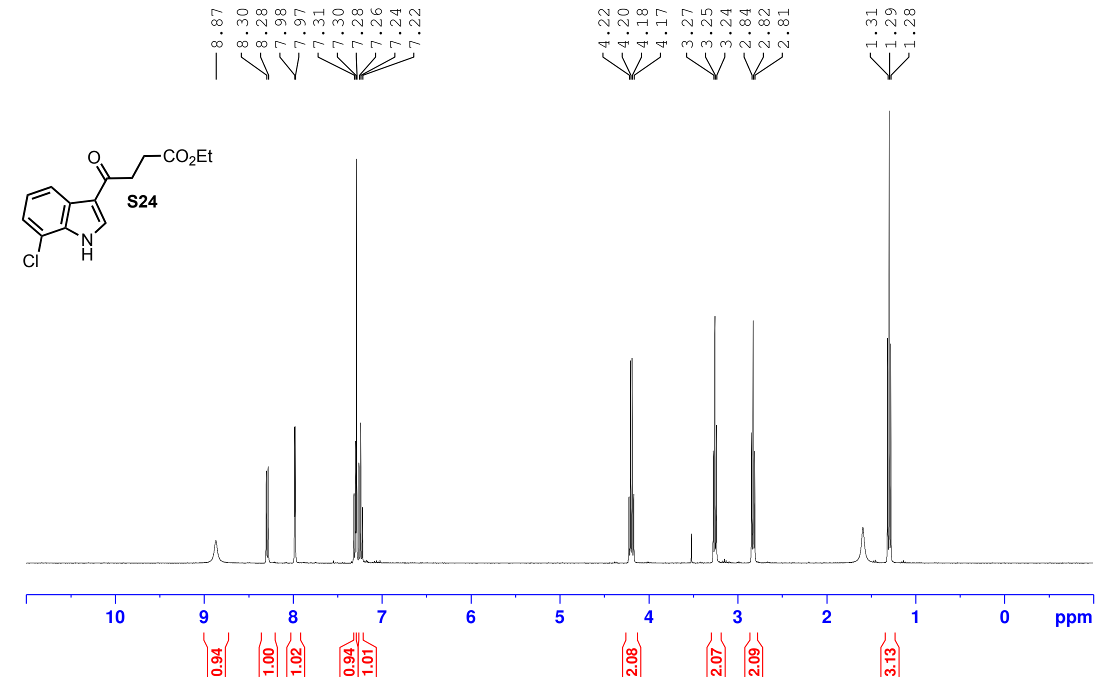

CN_3_59 - 1H NMR - 400.62 MHz - CDCl3

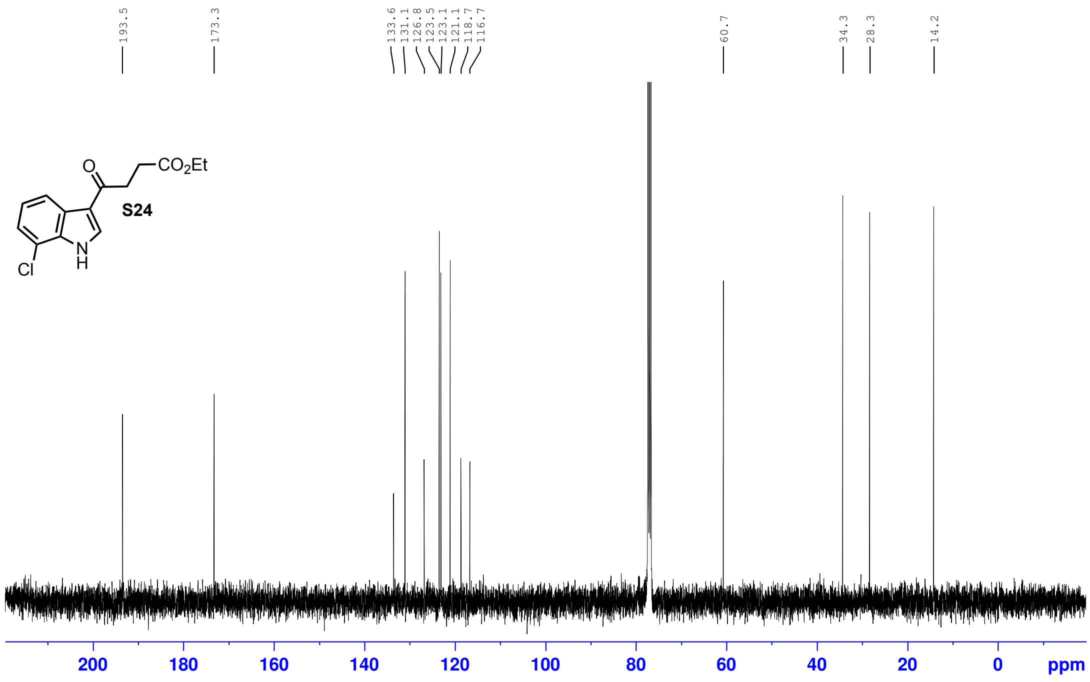

CN_3_59 - 13C NMR - 100.62 MHz - CDCl3

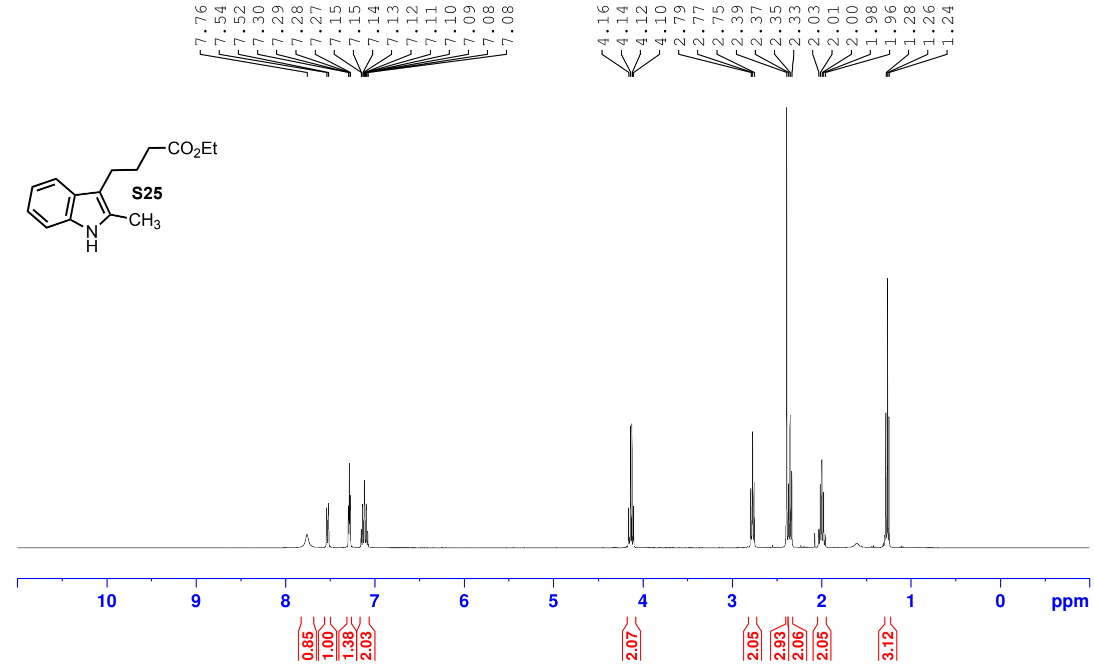

CN_3_6 - 1H NMR - 400.16 MHz - CDCl3

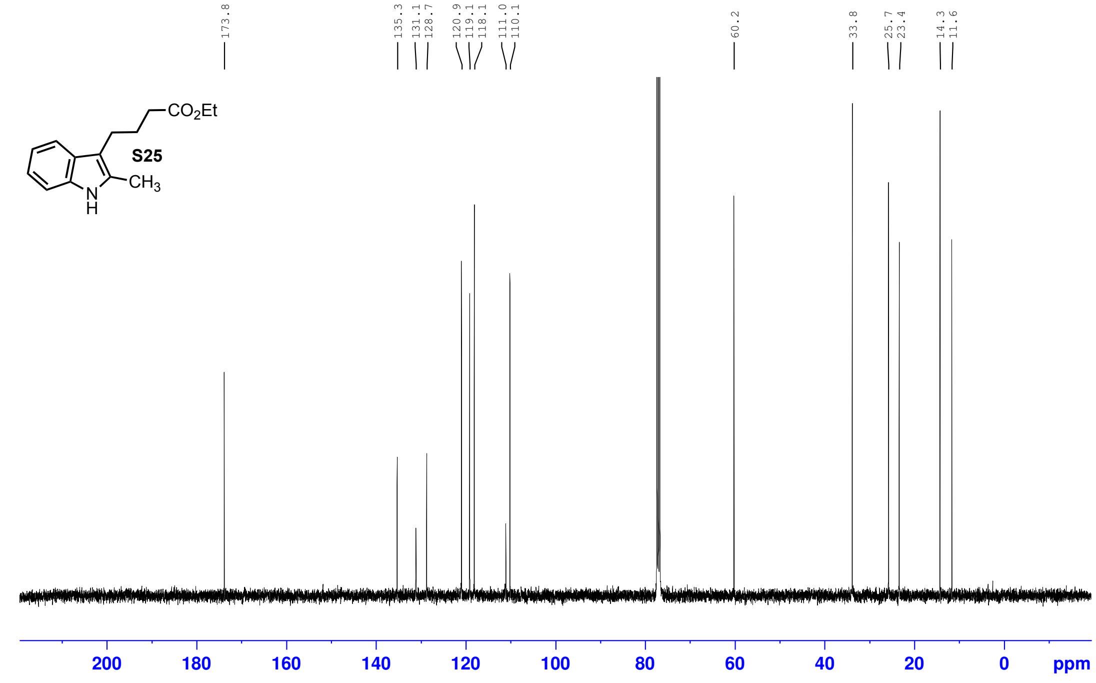

CN_3_6 - 13C NMR - 100.62 MHz - CDCl3

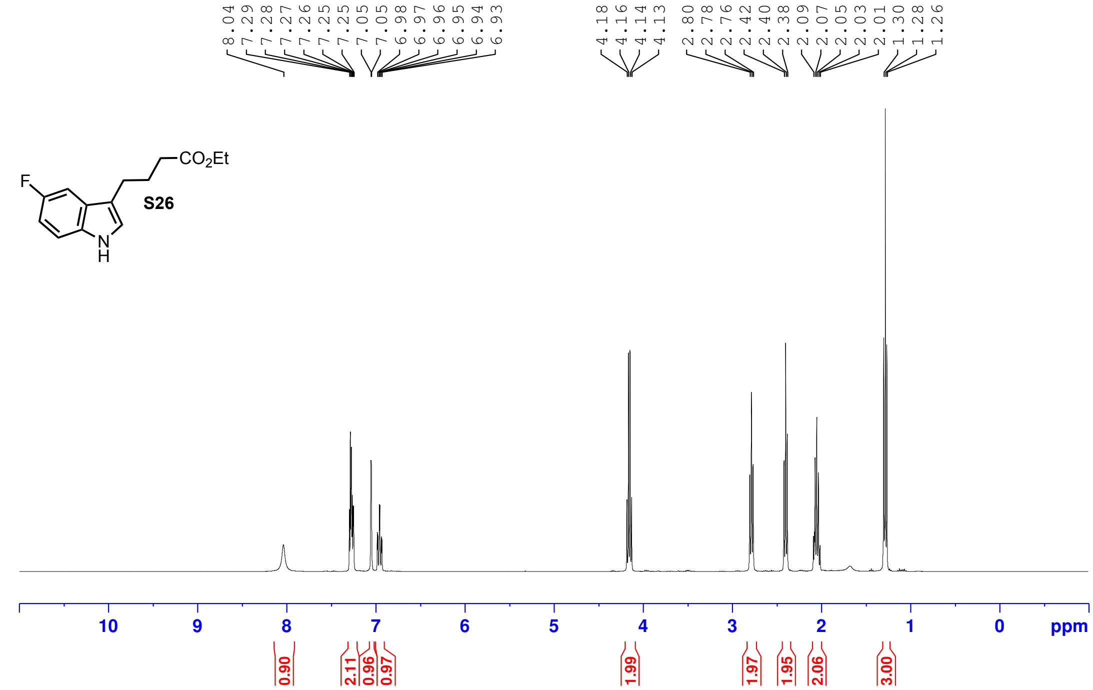

CN_3_53 - 1H NMR - 400.16 MHz - CDCl3

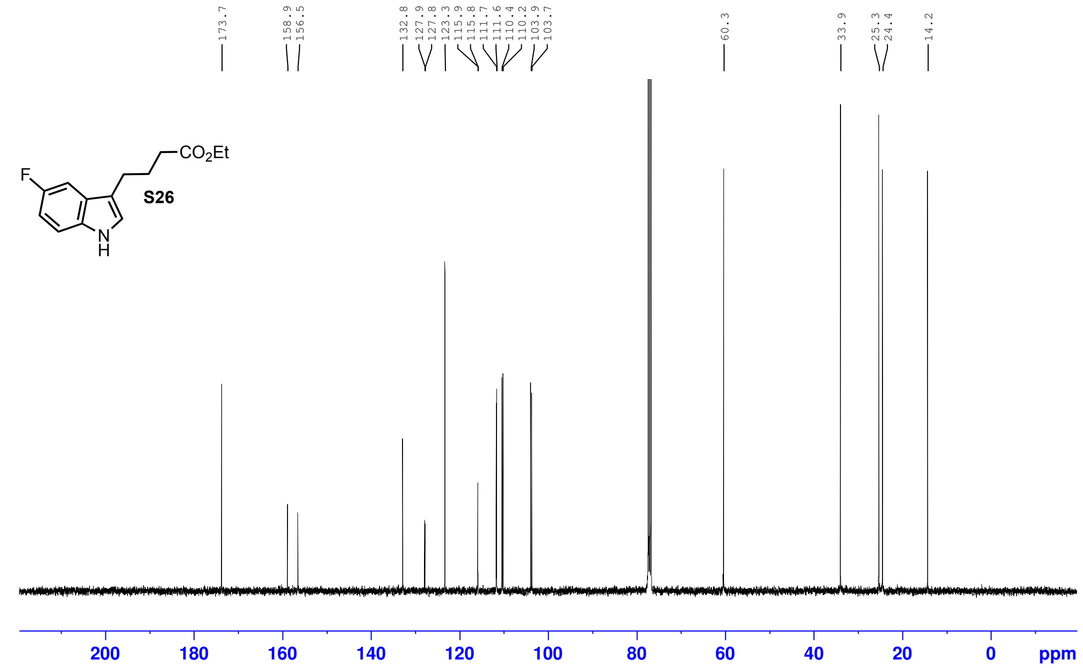

CN_3_53 - 13C NMR - 100.62 MHz - CDCl3

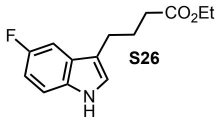

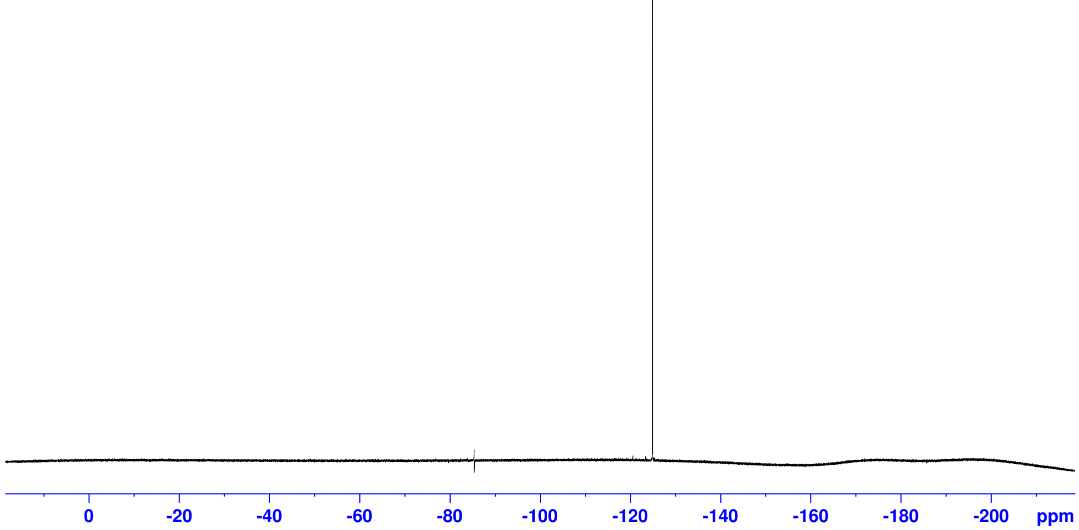

CN_3_53 - 19F NMR - 376.50 MHz - CDCl3

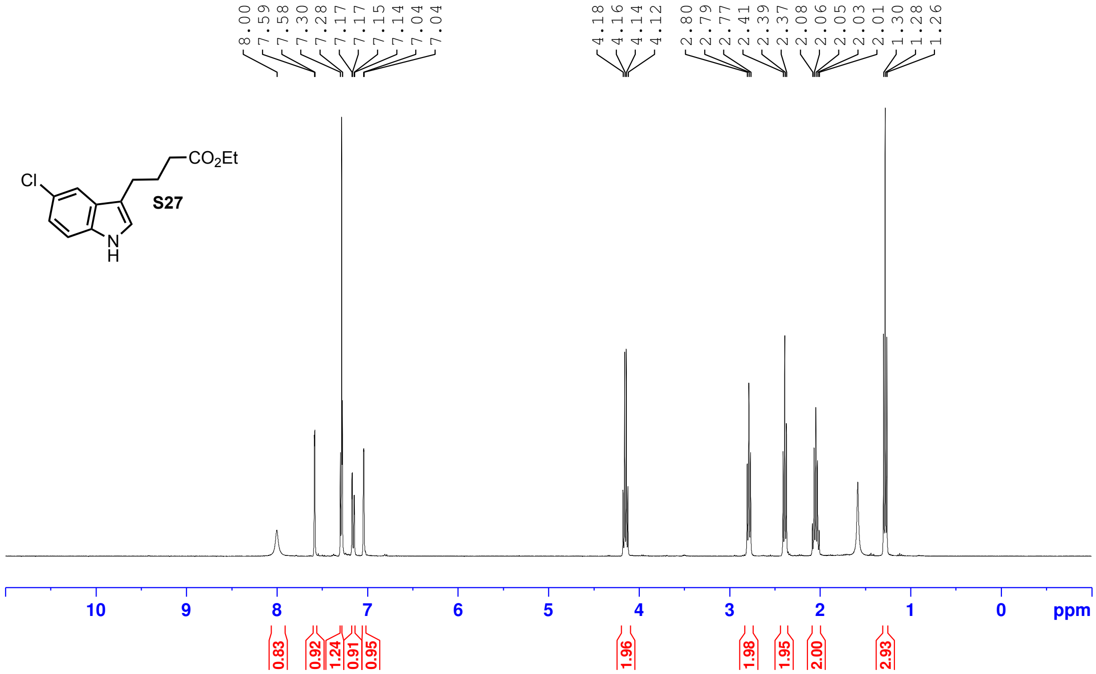

CN_3_17 - 1H NMR - 400.16MHz - CDCl3

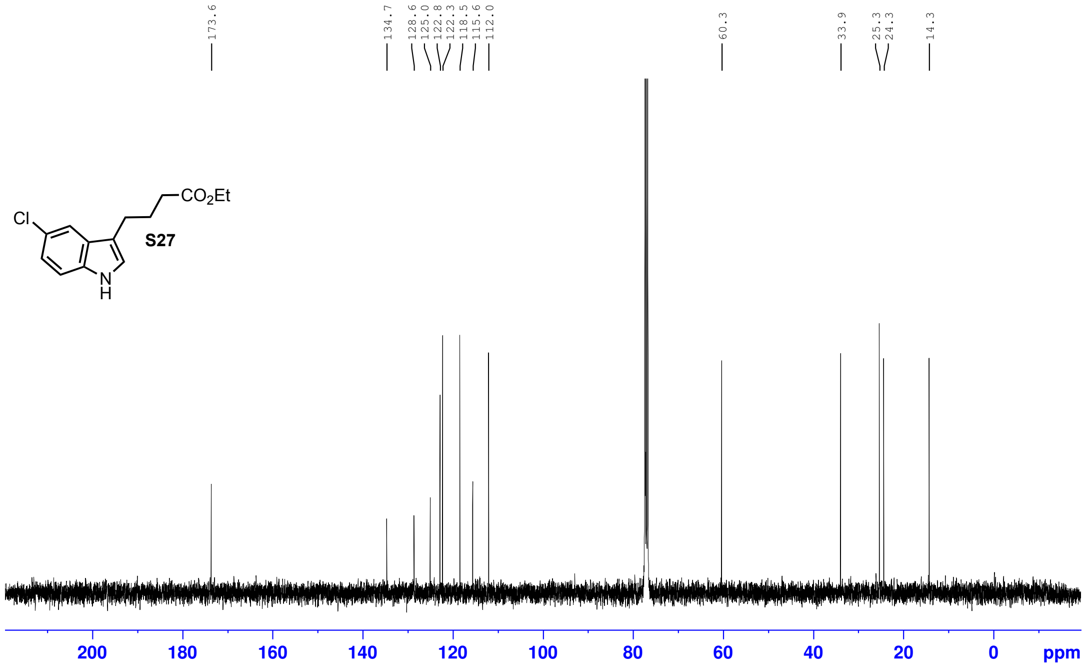

CN_3_17-13C NMR - 100.62 MHz - CDCl3

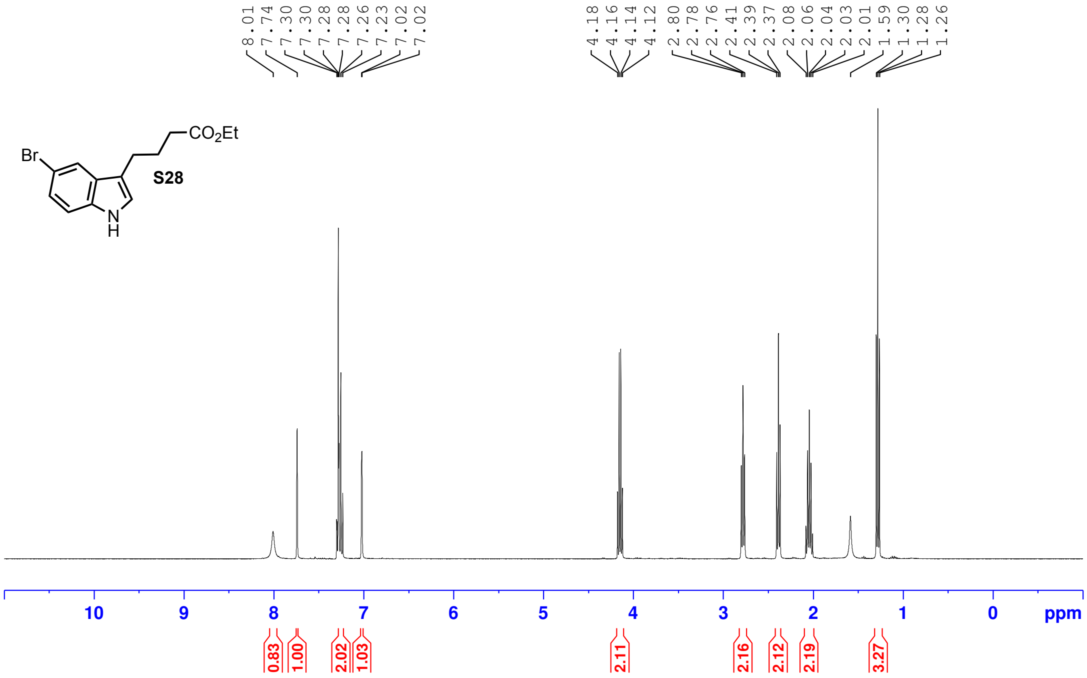

CN_2_86 - 1H NMR - 400.16 MHz - CDCl3

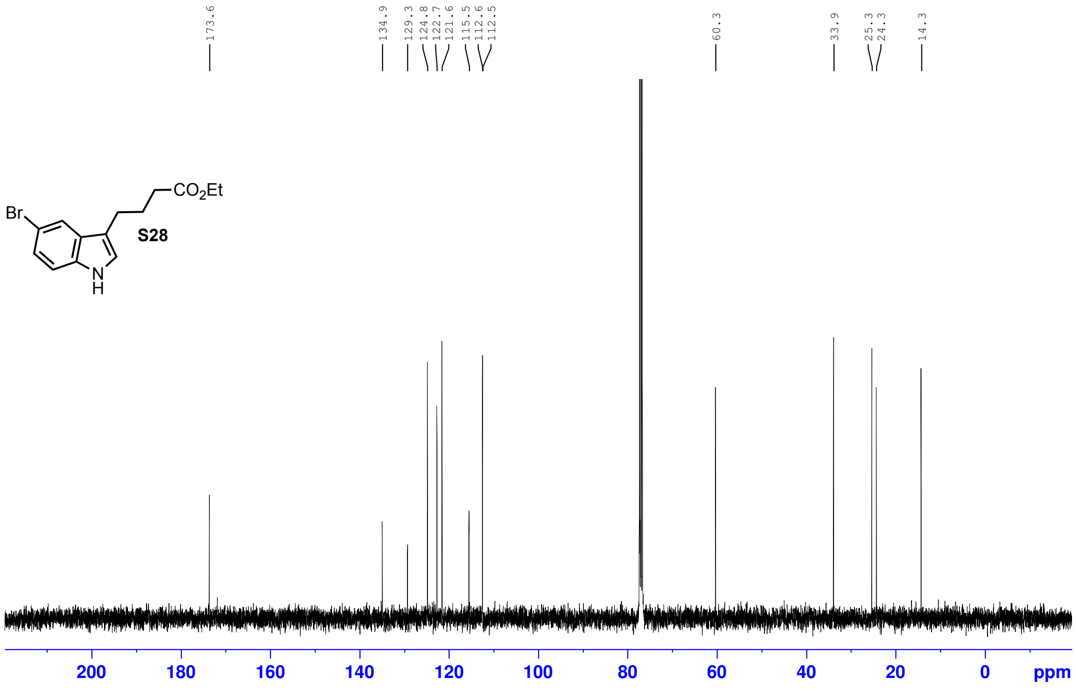

CN_2_86 - 13C NMR - 100.62 MHZ - CDCl3

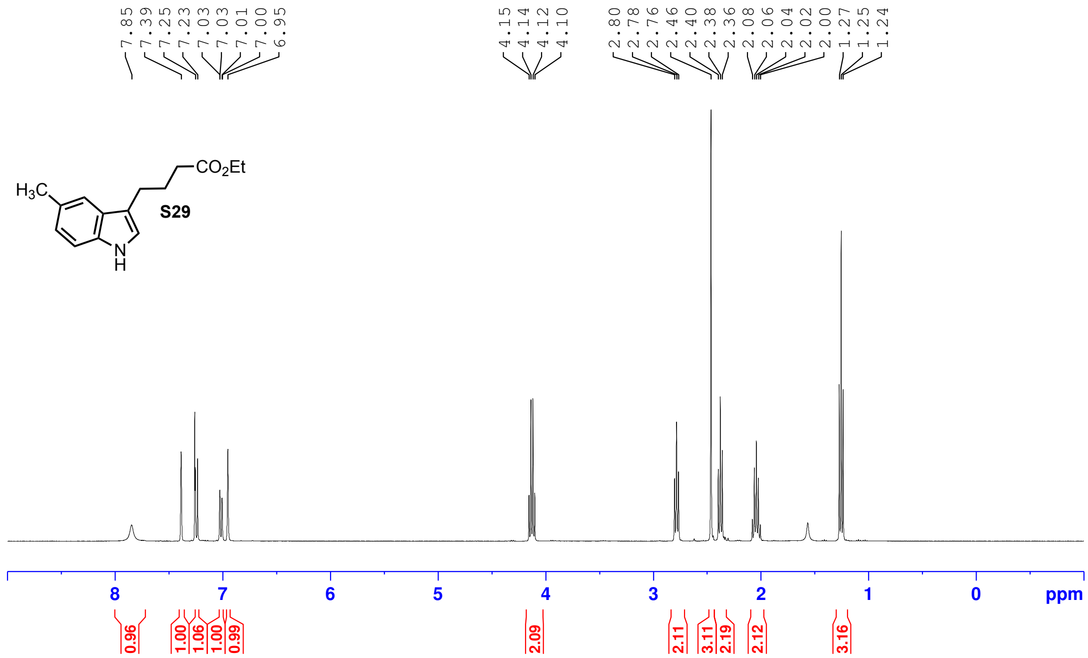

CN_3_66 - 1HNMR - 400.16 MHz - CDCl3

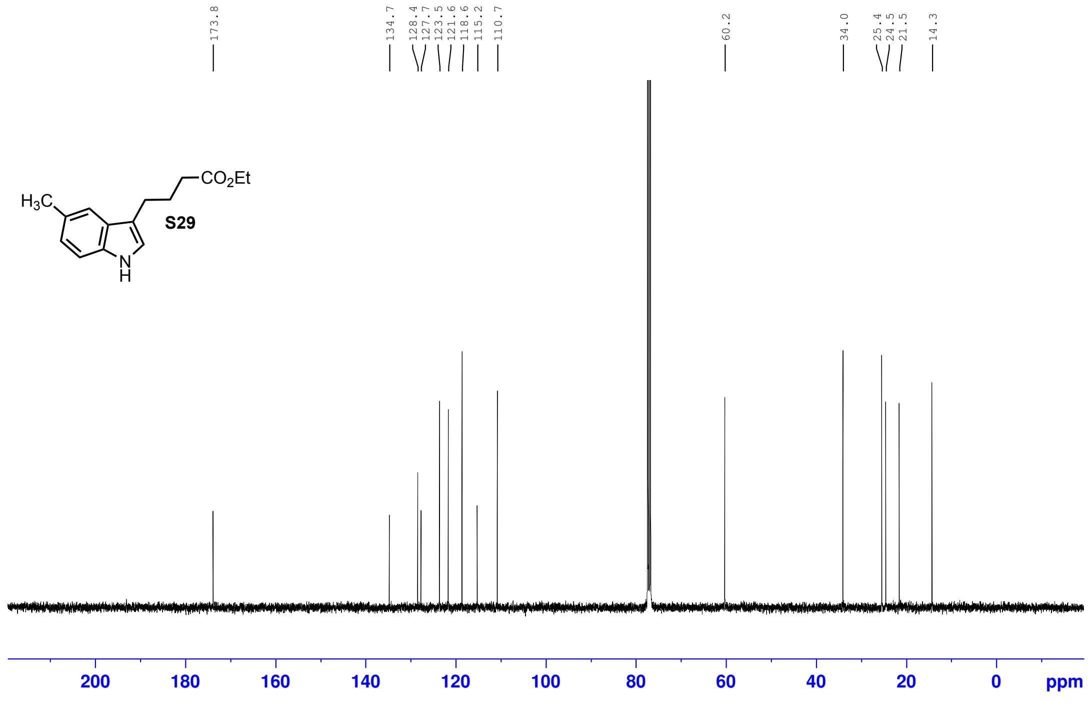

CN_3_66 - 13C NMR - 100.62 MHz - CDCl3

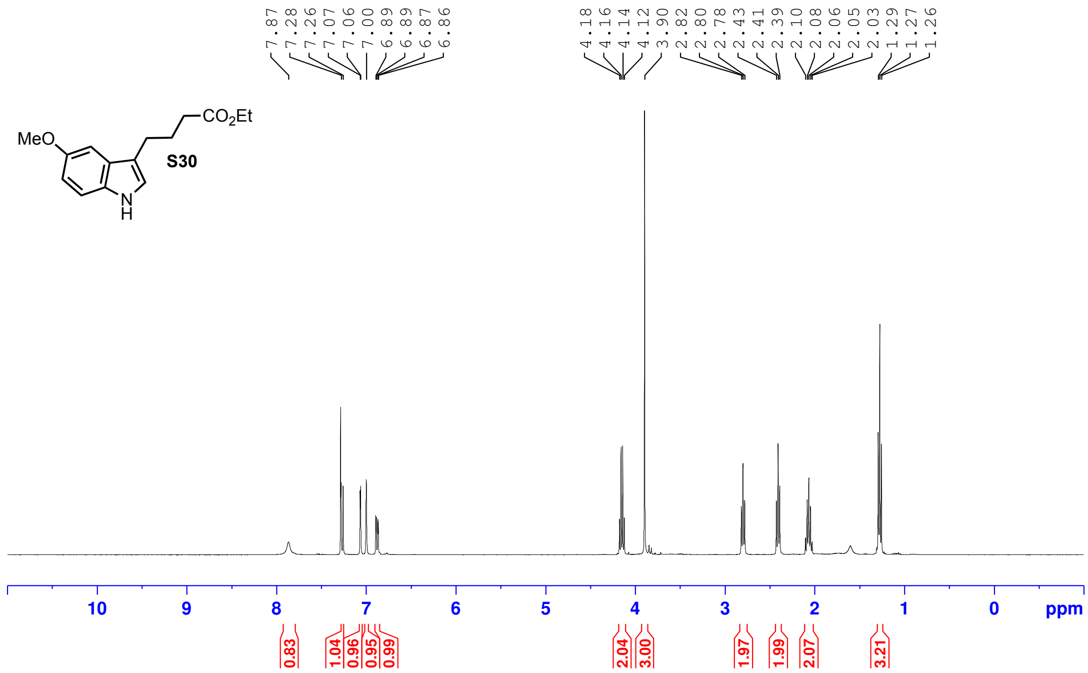

CN_3_52 - 1H NMR - 400.16 MHz - CDCl3

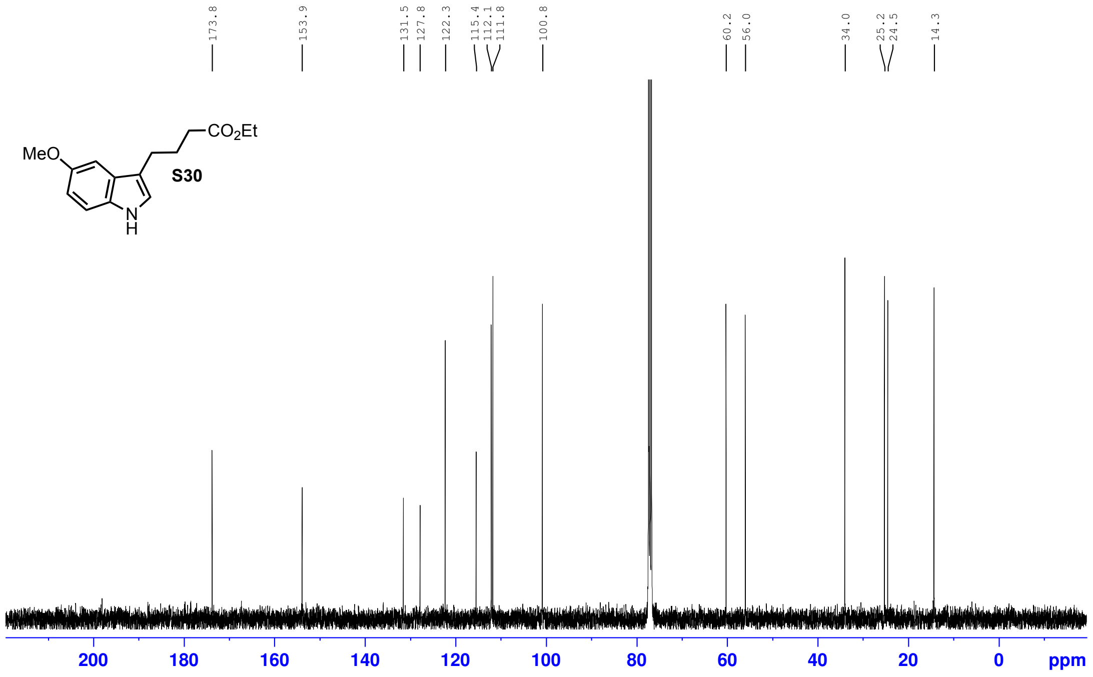

CN_3_52 - 13C NMR - 100.62 MHz - CDC

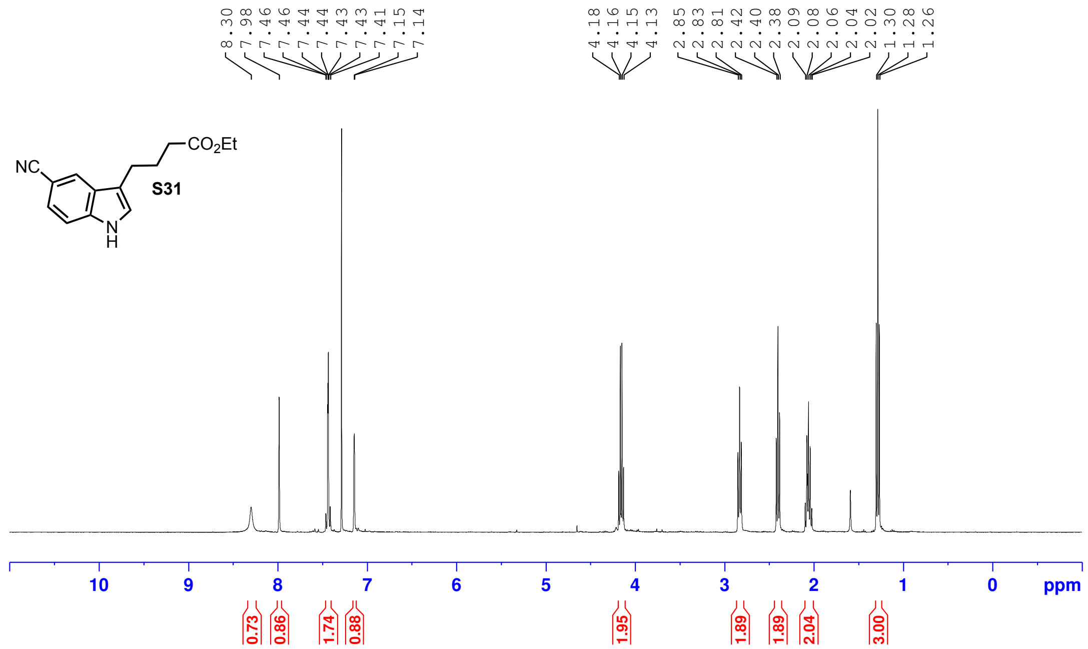

CN_3_43 - 1H NMR - 400.16 MHz - CDCl3

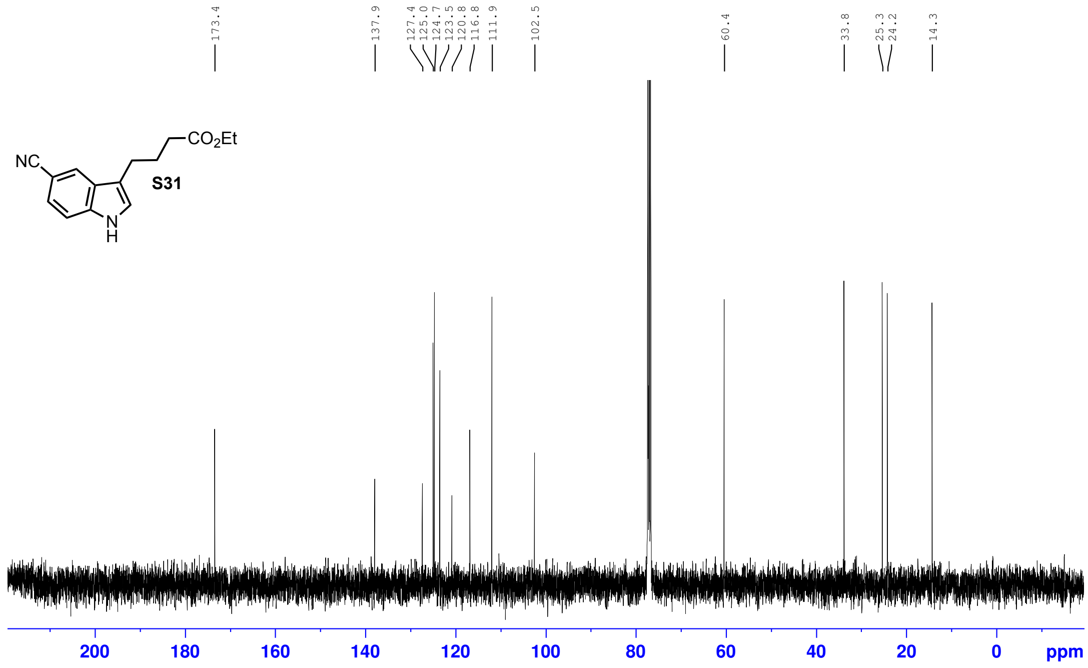

CN_3_43 - 13C NMR - 100.62 MHz - CDCl3

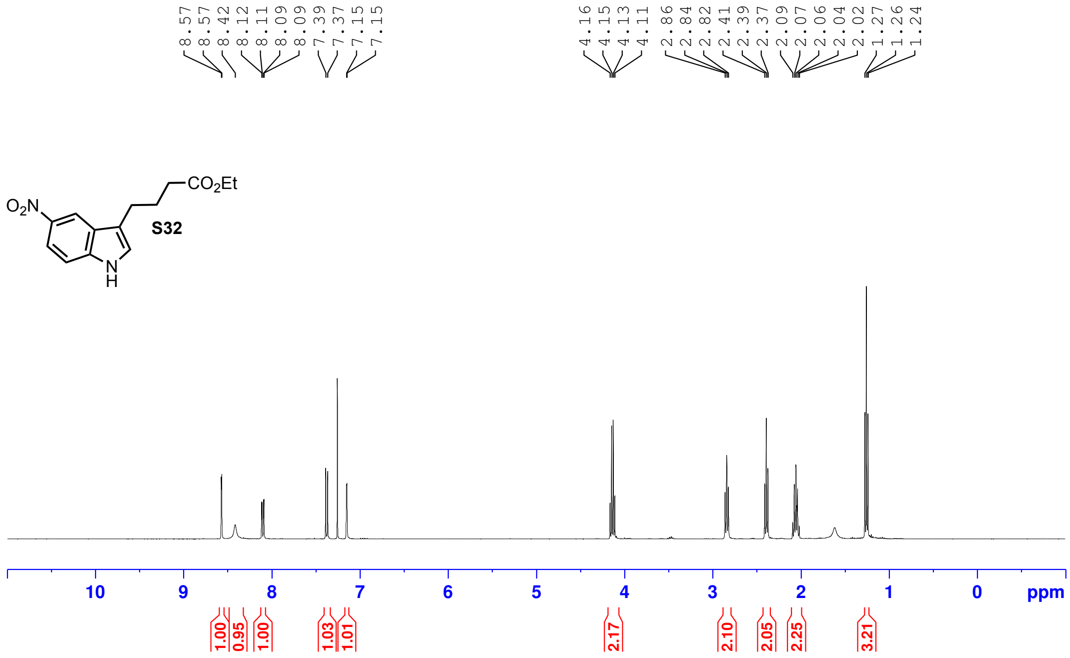

CN_3_72 - 1H NMR - 400.16 MHz - CDCl3

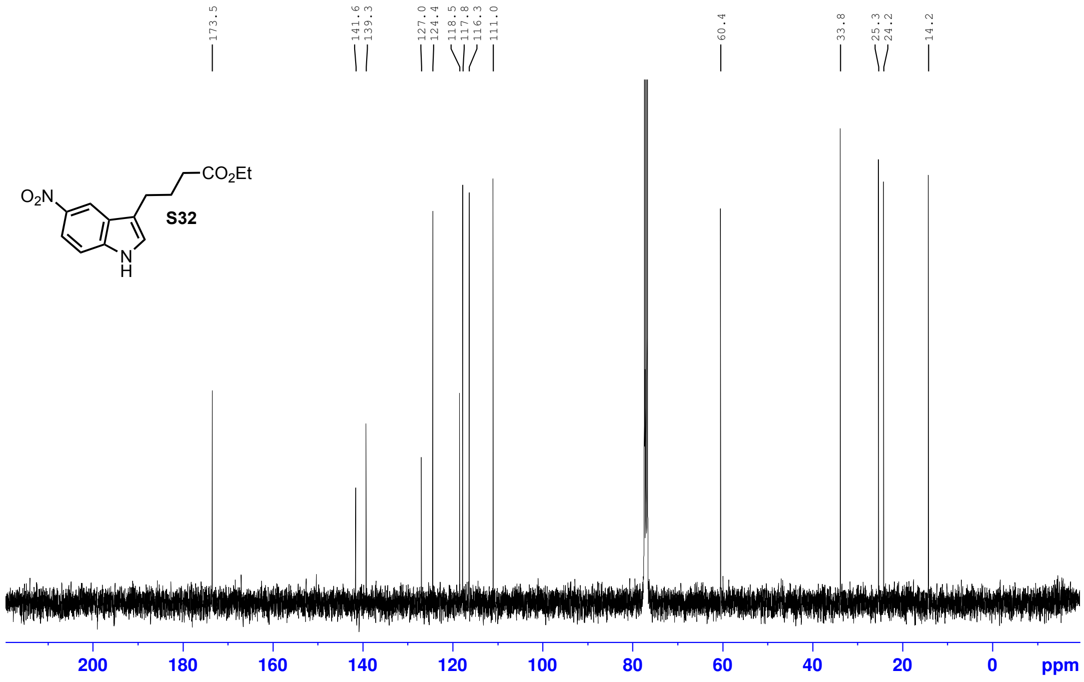

CN_3_72 - 13CNMR - 100.62 MHz - CDCl3

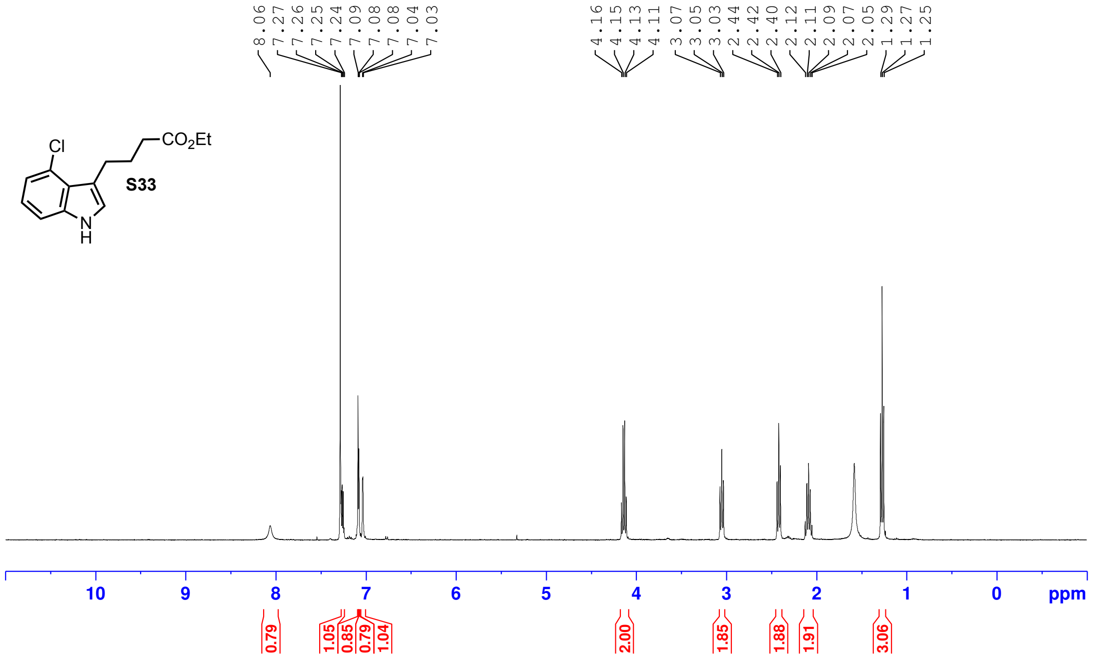

CN_3_34 - 1H NMR - 400.16 MHz - CDC

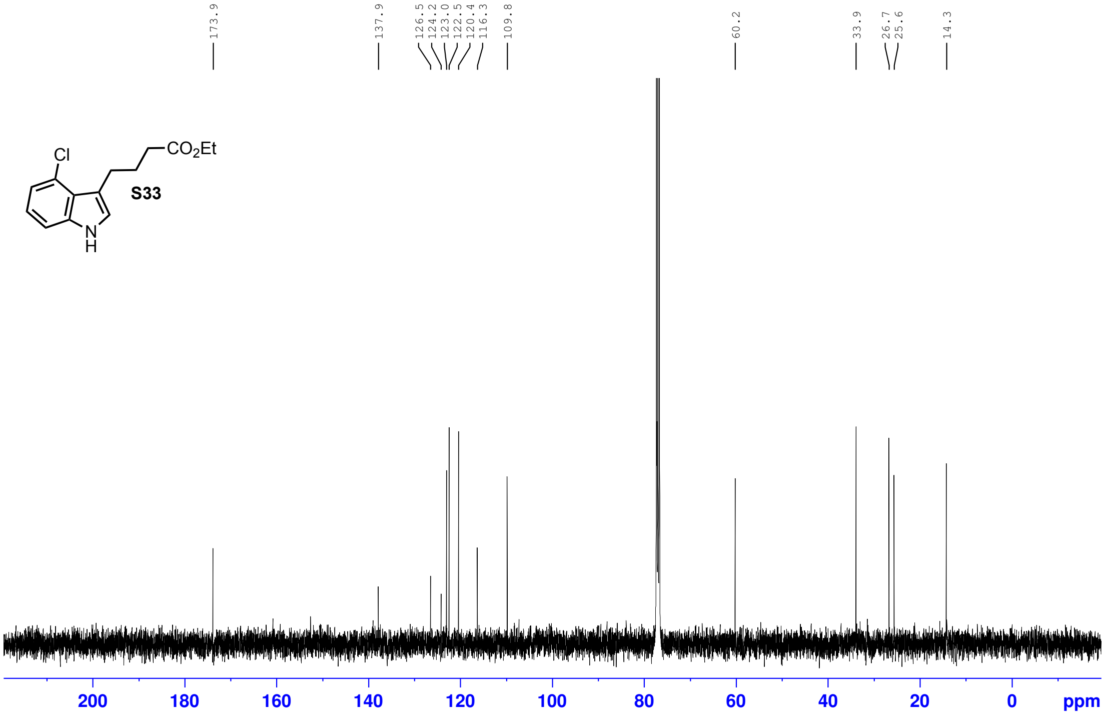

CN_3_34 - 13C NMR - 100.62 MHz - CDCl3

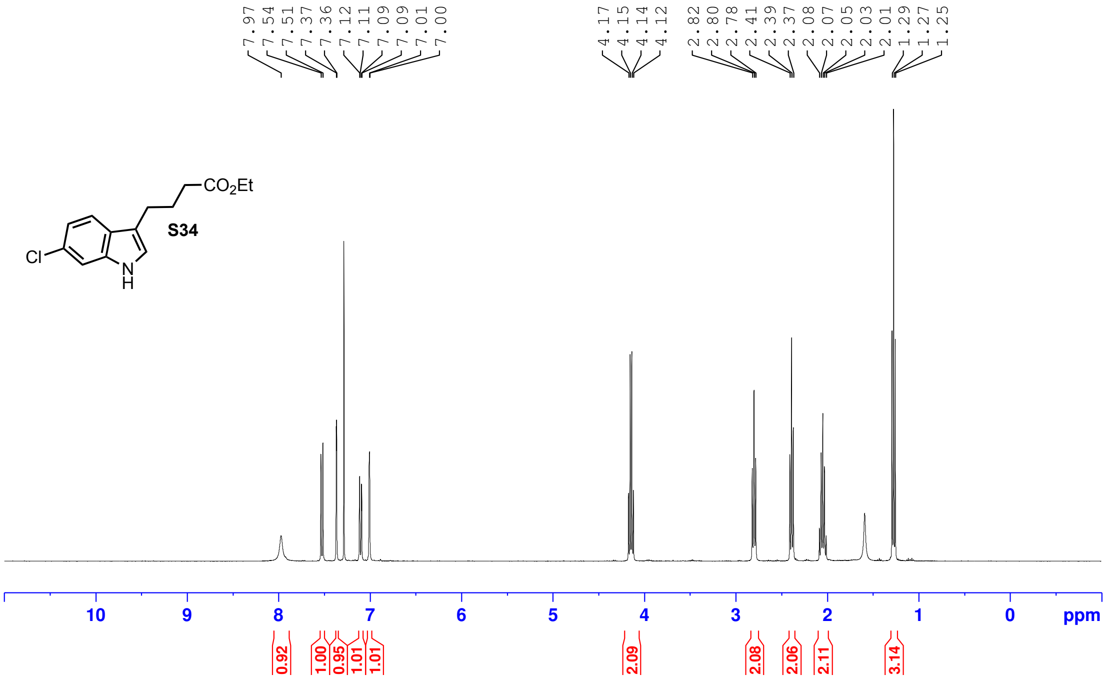

CN_3_25 - 1H NMR - 400.16 MHz - CDCl3

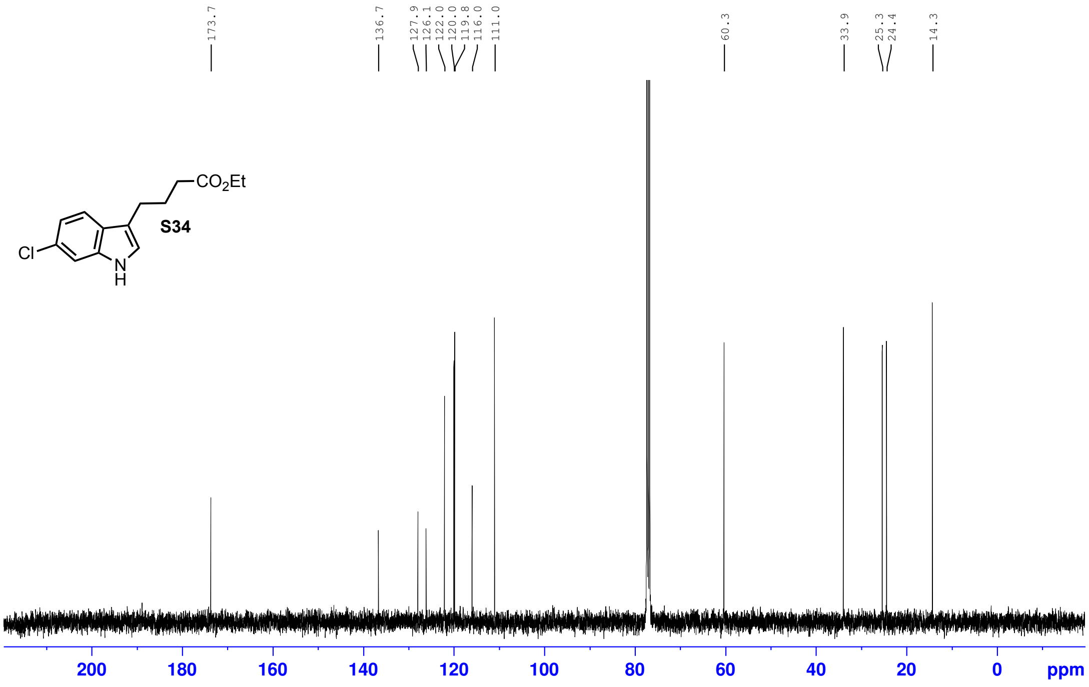

CN_3_25 - 13C NMR - 100.62 MHz - CDCl3

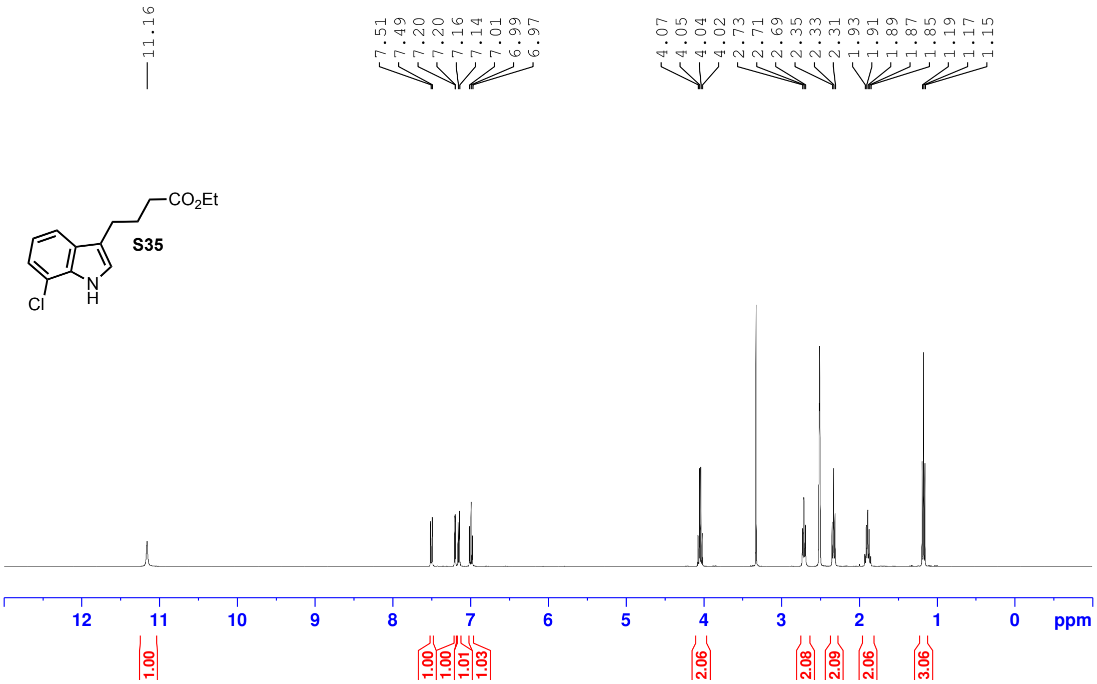

CN_3_60 - 1H NMR - 400.16 MHz - DMSO-d6

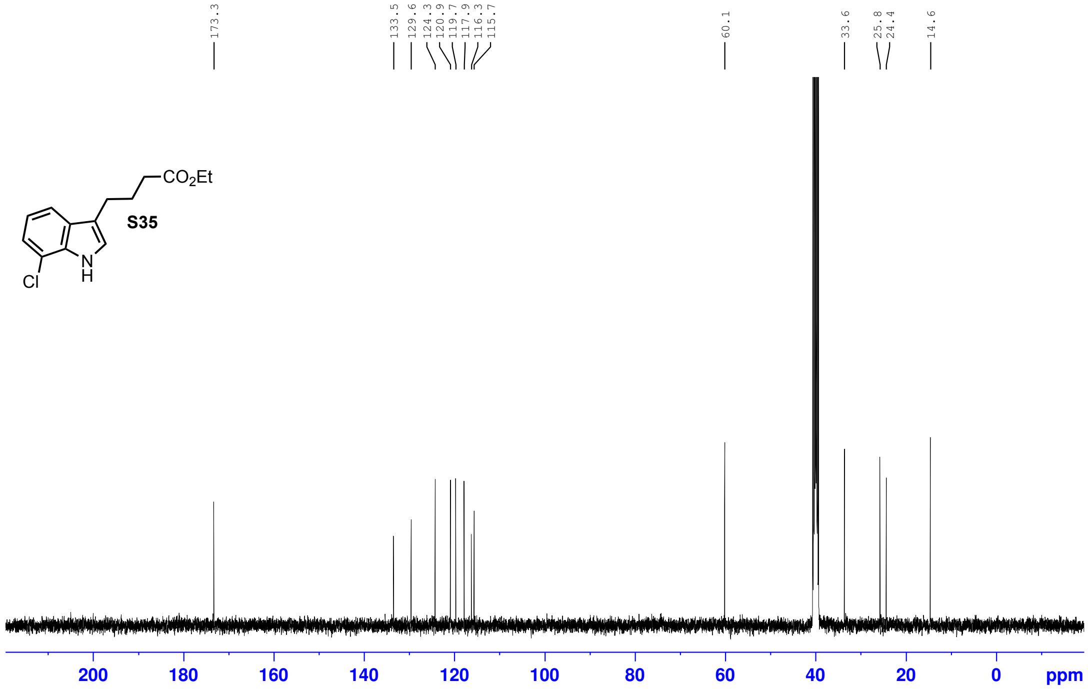

CN_3_60 - 13C NMR - 100.62 MHz - DMSO-d6

References

1) Judd, K. E.; Mahon, M. F.; Caggiano, L. Efficient synthesis of tetrahydro-β-carbolin-1-one and dihydroisoquinolin-1-one derivatives as versatile intermediates. *Synthesis* **2009**, 2809-2817.
2) Bremner, J. B.; Keller, P. A.; Pyne, S. G.; Boyle, T. P.; Brkic, Z.; David, D. M.; Robertson, M.; Somphol, K.; Baylis, D.; Coates, J. A.; Deadman, J.; Jeevarajah, D.; Rhodes, D. I. Synthesis and antibacterial studies of binaphthyl-based tripeptoids. Part 1. *Bioorg. Med. Chem.* **2010,** *18*, 2611-2620.
3) Chen, Y.; Zhang, L.; Zhang, L.; Jiang, Q.; Zhang, L. Discovery of indole-3-butyric acid derivatives as potent histone deacetylase inhibitors. *J. Enzyme Inhib. Med. Chem.* **2021,** *36*, 425-436.
4) Zeng, L.-Y.; Liu, Y.; Han, J.; Chen, J.; Liu, S.; Xi, B. O-Methylation of carboxylic acids with streptozotocin. *Org. Biomol. Chem.* **2022,** *20*, 5230-5233.
5) Okauchi, T.; Itonaga, M.; Minami, T.; Owa, T.; Kitoh, K.; Yoshino, H. A general method for acylation of indoles at the 3-position with acyl chlorides in the presence of dialkylaluminum chloride. *Org. Lett.* **2000,** *2*, 1485-1487.
6) Ketcha, D. M.; Lieurance, B. A.; Homan, D. F. J.; Gribble, G. W. Synthesis of alkyl-substituted N-protected indoles via acylation and reductive deoxygenation. *J. Org. Chem.* **1989,** *54*, 4350.
7) Guchhait, S. K.; Kashyap, M.; Kamble, H. ZrCl4-Mediated Regio- and Chemoselective Friedel-Crafts Acylation of Indole. *J. Org. Chem.* **2011,** *76*, 4753-4758.
8) McLaughlin, E. C.; Norman, M. W.; Ko Ko, T.; Stolt, I. Three-component synthesis of disubstituted 2H-pyrrol-2-ones: preparation of the violacein scaffold. *Tetrahedron Lett.* **2014,** *55*, 2609-2611.
9) Csende, F. A simple procedure for synthesis of roxindole, a dopamine D2-receptor agonist. *Arch. Pharm.* **2001,** *334*, 253-254.
10) Yamane, D.; Tanaka, H.; Hirata, A.; Tamura, Y.; Takahashi, D.; Takahashi, Y.; Nagamitsu, T.; Ohtawa, M. One-Pot γ-Lactonization of Homopropargyl Alcohols via Intramolecular Ketene Trapping. *Org. Lett.* **2021,** *23*, 2831-2835.
11) Hong, J. E.; Yoon, J.; Baek, W.; Kim, K.; Kwak, J.-H.; Park, Y. Electrochemical C(sp3)-H Lactonization of 2-Alkylbenzoic Acids toward Phthalides. *Org. Lett.* **2023,** *25*, 298-303.
